# Asymmetric Mannich/Cyclization Reaction of 2-Benzothiazolimines and 2-Isothiocyano-1-indanones to Construct Chiral Spirocyclic Compounds

**DOI:** 10.3390/molecules29132958

**Published:** 2024-06-21

**Authors:** Yao Zheng, Da-Ming Du

**Affiliations:** 1 School of Chemistry and Chemical Engineering, Beijing Institute of Technology, No. 5 Zhongguancun South Street, Beijing 100081, China; 3220211480@bit.edu.cn; 2 Key Laboratory of Medicinal Molecule Science & Pharmaceutical Engineering, Ministry of Industry and Information Technology, No. 5 Zhongguancun South Street, Beijing 100081, China

**Keywords:** benzothiazole, imine, Mannich reaction, 2-isothiocyanato-1-indanone

## Abstract

An efficient and practical organocatalyzed asymmetric Mannich/cyclization tandem reaction strategy of 2-benzothiazolimines and 2-isothiocyanato-1-indanones was developed, and novel spirocyclic compounds containing benzothiazolimine and indanone scaffolds were obtained. This chiral thiourea-catalyzed Mannich/cyclization tandem reaction offers chiral spirocyclic compounds with continuous tertiary and quaternary stereocenters in good to high yields (up to 90%) with excellent diastereoselectivities (up to >20:1 dr) and enantioselectivities (up to 98% ee) at −18 °C. Additionally, the scaled-up synthesis was also performed with retained yield and stereoselectivity, and a reaction mechanism was also proposed.

## 1. Introduction

**Figure 1:**
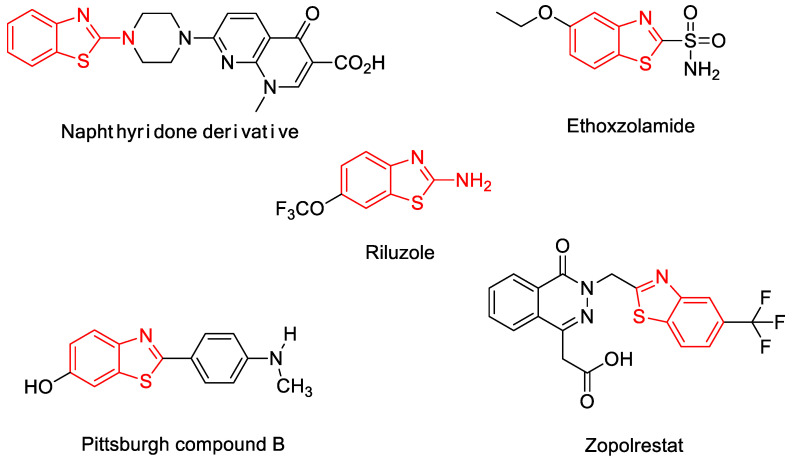
Representative examples of benzothiazole derivatives with biological relevance.

Benzothiazole compounds are an important class of heterocyclic compounds with a wide range of biological activities [1]. They are also the core structures that constitute many natural products and important drugs [2]. Benzothiazole derivatives are common heterocyclic skeletons in many natural or synthetic products and have recognized biological and pharmacological properties [3,4,5,6], such as antibacterial agents, bactericides, anticancer agents, antioxidants, anti-inflammatory agents, analgesics, antiviral agents, anticonvulsants, antituberculosis agents, antidiabetic agents, antileishmaniasis agents, antihistamines, antimalarial agents, antidepressants, and enzyme and receptor agonists/antagonists. In addition, some benzothiazole derivatives have been shown to have activity against neurodegenerative diseases [7]. For example, the naphthyridone derivative 7-[4-(1,3-benzothiazol-2-yl)piperazin-1-yl]-1-methyl-4-oxo-1,4-dihydro-1,8-naphthyridine-3-carboxylic acid is a promising anti-HIV agent due to its ability to inhibit the HIV-1 Tat-mediated transcription and the potent antiviral activity observed in acutely, chronically, and latently infected cells [8] (Figure 1), and ethoxzolamide is a type of sulfonamide drug, primarily used as a diuretic and carbonic anhydrase inhibitor [9]. Riluzole is currently on the market for the treatment of amyotrophic lateral sclerosis (ALS) [10,11], Pittsburgh compound B is used as a positron emission tomography (PET) imaging agent for Alzheimer’s disease (AD) [12], and zopolrestat is an effective oral aldose reductase inhibitor, and has research value in the complications of diabetes [13]. Given the value of benzothiazole derivatives, developing new synthesis methods to obtain novel benzothiazole derivatives remains of great significance [14]. For more than a decade, the value of benzothiazole imine in synthesizing bioactive and pharmacological substances, combining bioactive structural units, and discovering new bioactive substances has continuously emerged.

Currently, utilizing 2-benzothiazolimine as a synthetic unit to construct heterocyclic compounds through asymmetric catalytic reactions has emerged as a crucial and efficient method for synthesizing biologically active molecules. This approach holds significant importance in establishing diverse drug-related molecular libraries and discovering novel bioactive substances. As a class of benzothiazole-containing synthons, 2-benzothiazolimines are considered as universal precursors for the preparation of these heterocycles and has thus been extensively applied in numerous asymmetric transformations. According to the literature, 2-benzothiazolimines often serve as Mannich acceptors in asymmetric synthesis [15,16,17]. Due to the weak aromaticity of the thiazole system, 2-benzothiazolimines can be employed as conjugated imines and further utilized as C4-synthons in asymmetric organic synthesis, particularly in asymmetric cyclization processes catalyzed by *N*-heterocyclic carbenes [18], phosphates [19], squaramides [20], guanidines [21], and chiral amines [22].

**Scheme 1:**
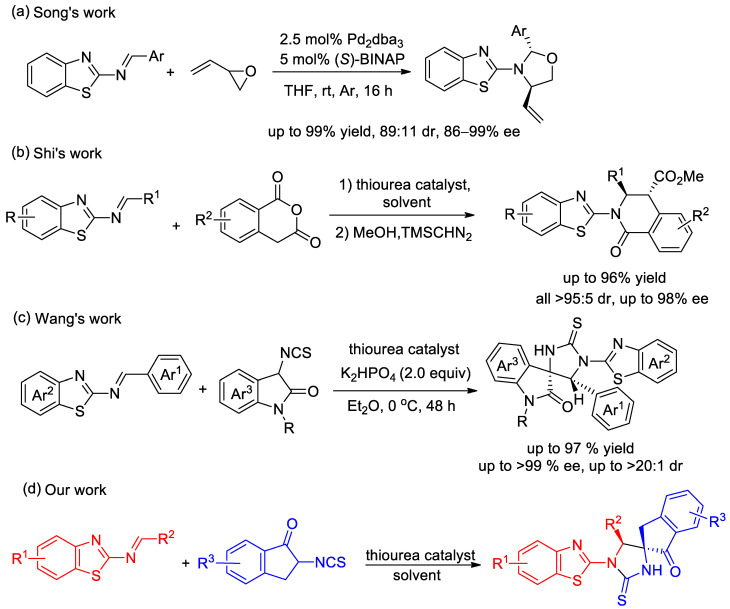
Previous reports and our work. (**a**) [2 + 3] cyclization reaction [23]; (**b**) [2 + 4] cyclization reaction [24]; (**c**) [2 + 3] cyclization reaction [25]; (**d**) our research plan.

However, during our literature review, we noted a limited number of studies focusing on the cycloaddition reactions involving C2-synthon transformations. For example, in 2019, Song’s group published an article reporting a palladium-catalyzed asymmetric [3 + 2] cycloaddition between vinyl epoxides and 2-benzothiazolimine [23] (Scheme 1a). In 2022, Shi’s group innovatively developed a novel class of chiral organic small-molecule catalysts derived from axial chiral styrene. These novel chiral catalysts have been effectively utilized in the asymmetric [2 + 4] cyclization of 2-benzothiazolines with homophthalic anhydrides. This approach enables control over the chemical, diastereoselectivity, and enantioselectivity of the reaction, thereby addressing the challenges associated with catalyzing asymmetric [2 + n] cyclization reactions involving 2-benzothiazolimines [24] (Scheme 1b). Wang’s group utilized 2-benzothiazoleimines as unusual C2-synthons and obtained highly functionalized and indole-fused cyclic thiourea scaffolds bearing benzothiazole cores through thiourea-based bifunctional phosphonium salt catalysis. The corresponding products were obtained in high yields with excellent diastereo- and enantioselectivities [25] (Scheme 1c). Inspired by their work, we will continue our project to obtain bioactive heterocycles using 2-benzothiazolimines as C2-synthons. Herein, the organocatalyzed asymmetric [2 + 3] cyclization reaction of 2-benzothiazolimines with 2-isothiocyanato-1-indanones to generate chiral spirocyclic compounds was developed, in order to consolidate and develop this research achievement (Scheme 1d).

## 2. Results and Discussion

### 2.1. Optimization of Reaction Conditions

**Figure 2:**
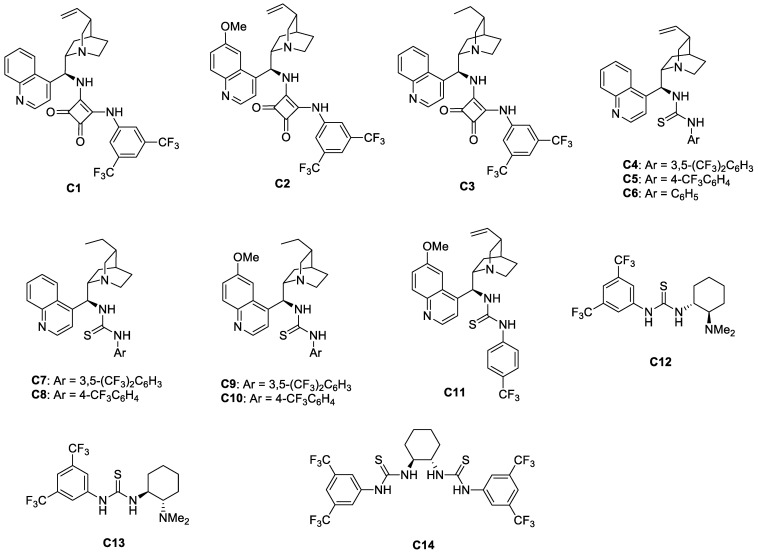
Organocatalysts selected.

In the initial study, 0.10 mmol of (*E*)-*N*-(benzo[*d*]thiazol-2-yl)-1-phenylcarbazone (**1a**) and 0.12 mmol of 2-isothiocyanato-1-indanone (**2a**) were chosen as the template reaction substrates for cyclization. Then, 10 mol% of cinchonidine-derived squaramide catalyst **C1** was added, and the reaction was conducted at room temperature in 1 mL of dichloromethane for 18 h. The target product **3aa** was finally obtained in 75% yield with good stereoselectivity (83% ee, >20:1 dr). Encouraged by this important result, we screened several organocatalysts (Figure 2), reaction solvents, and catalyst loadings to further improve the outcome and enantioselectivity. The results are outlined in Table 1.

The results indicated that catalysts **C4**, **C5**, **C9**, **C11**, and **C13** exhibited similar or slightly inferior catalytic performance compared to **C1** (Table 1, entries 4, 5, 9, 11, and 13). When the reaction was catalyzed by bifunctional thiourea catalyst **C6** or **C14**, the enantioselectivity decreased sharply, suggesting that these two types of catalysts were not suitable for this reaction (Table 1, entries 6 and 14). Encouragingly, catalysts **C2**, **C7**, **C8**, and **C12** outperformed catalyst **C1** in terms of enantioselectivity, and catalyst **C7** exhibited a higher yield and the optimal enantioselectivity among them (Table 1, entries 2, 7, 8, and 12). Through the data comparison, it can be determined that the optimal catalyst was **C7**.

With the best catalyst in hand, various solvents were screened, including 1,4-dioxane, tetrahydrofuran, ethyl acetate, 1,2-dichloroethane (DCE), acetonitrile, and methyl tert-butyl ether (MTBE) (Table 1, entries 15–20). The experimental results revealed that dichloromethane was the optimal solvent for this reaction under the same experimental conditions. Following this, the effects of catalyst loading and temperature were investigated (Table 1, entries 21–23). When the catalyst loading was reduced from 10 mol% to 5 mol% or increased to 15 mol%, the enantioselectivity of the reaction decreased significantly, while the yield changed marginally. Finally, when the temperature was lowered to −18 °C, the enantioselectivity of the reaction improved (to 98% ee). Therefore, the optimal reaction conditions for the Mannich/cyclization reaction between **1a** and **2a** were using 10 mol% **C7** as the catalyst, 1 mL dichloromethane as the solvent, and conducting the reaction at −18 °C.

### 2.2. Substrate Scope

**Scheme 2:**
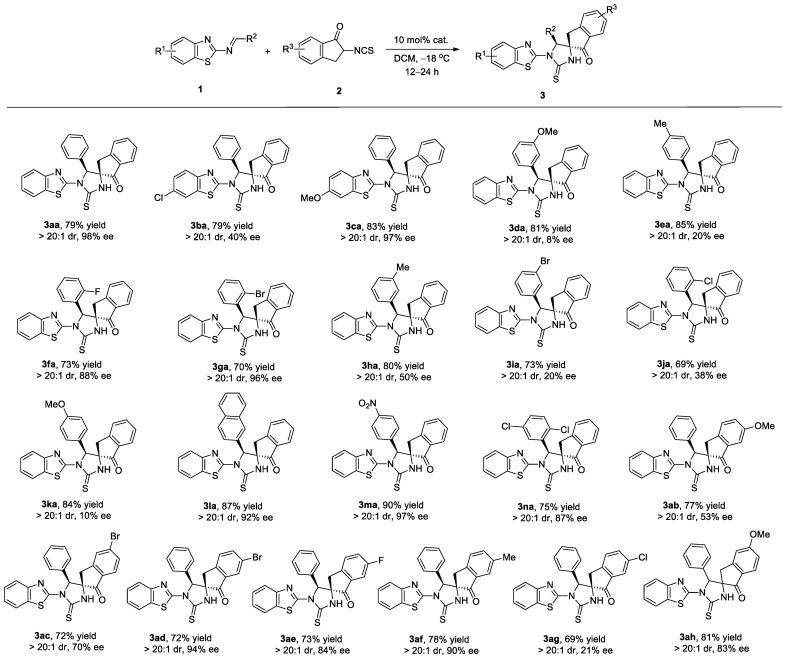
Substrate scope for spirocyclic compounds **3**. The reactions were carried out with **1** (0.10 mmol), **2** (0.12 mmol), and catalyst **C7** (10 mol%) in DCM (1.0 mL) at −18 °C for 12–24 h. The yields were isolated after column chromatography. The dr values were determined by ^1^H NMR and the ee values were determined by HPLC analysis.

With the optimized conditions in hand, we then began to investigate the substrate scope and limitation of this reaction, and the results are summarized in Scheme 2.

First, the substrate scope of 2-benzothiazolimine **1** was investigated. The influence of the substituent R^1^ on the benzene ring of the benzothiazole skeleton was initially evaluated. By introducing the electron-withdrawing group –Cl and the electron-donating group –OMe at the C-6 position of the benzene ring, two related products were obtained. By comparison, when the substituent on the benzene ring was the electron-withdrawing group –Cl, the related product **3ba** exhibited excellent diastereoselectivity (>20:1 dr) and good yield (79%) but had a relatively low enantioselectivity (40% ee). In contrast, when the substituent on the benzene ring was the electron-donating group –OMe, the related product **3ca** had excellent enantioselectivity (98% ee) and good diastereoselectivity (>20:1 dr), along with a high yield (83%). These results indicated that the electronic nature of the substituent on the benzene ring played a crucial role in the reaction outcome.

Simultaneously, the situation of the substituent R^2^ in 2-benzothiazolimine **1** was also examined. When the C-2 position of substituent R^2^ was substituted by halogen atoms F, Cl, and Br, the related products **3fa**, **3ga**, and **3ja** exhibited good yields (69–73%). Products **3fa** and **3ga** both had excellent enantioselectivity (88% ee and 96% ee, respectively), but the enantioselectivity of product **3ja** was lower (38% ee). The experiments revealed that when the substituent at the C-3 position of R^2^ was an electron-donating alkyl group (methyl) or methoxy group (products **3ha** and **3da**), the enantioselectivity of the products decreased significantly (50% ee and 8% ee, respectively). However, when the substituent at the C-3 position was an electron-withdrawing Br (product **3ia**), it still had a relatively low enantioselectivity (20% ee). This may have been due to the fact that the meta-substituted benzene ring still possessed a certain degree of steric hindrance, which, although not effectively hindering the reaction process, significantly affected the enantioselectivity of the reaction.

When the substituent at the C-4 position of R^2^ was an electron-donating alkyl group (methyl) or methoxy group (products **3ea** and **3ka**), they had high yields (85% and 84%, respectively), but unfortunately, both products had low enantioselectivities (20% ee and 10% ee, respectively). Conversely, when the substituent at the C-4 position was an electron-withdrawing nitro group, the product exhibited the optimal yield (90%) and excellent enantioselectivity (97% ee). When R^2^ was a naphthyl group, the reaction still proceeded with a high yield (87%) and excellent stereoselectivity (>20:1 dr, 92% ee) to produce the corresponding product **3la**.

Furthermore, this study also attempted to use a doubly substituted benzene ring at the position of substituent R^2^, resulting in product **3na**. Fortunately, it also exhibited high enantioselectivity (87% ee) and a good yield (75%).

The further expansion of the substrate scope focused on 2-isothiocyanato-1-indanone **2** by introducing substituents R^3^ on the benzene ring of 2-isothiocyanato-1-indanone **2**. When an electron-withdrawing Br group was introduced at the C-5 position C of the indanone (product **3ac**), compared to introducing an electron-donating methoxy group (product **3ah**), both the yield and enantioselectivity decreased. This may have been due to the strong electron-withdrawing ability of the Br atom, which reduced the reaction activity.

Subsequently, the introduction of halogen atoms at the C-6 position of the indanone was investigated. Among them, the two products substituted by Br (product **3ad**) and F (product **3ae**) both exhibited good yields and excellent stereoselectivities, but the yield and enantioselectivity of the product substituted by Cl (product **3ag**) showed significant declines. When a methoxy group (product **3ab**) and a methyl group (product **3af**) were introduced at the C-6 position of the indanone, both had good yields, but the enantioselectivity of product **3af** was significantly higher than that of **3ab**. The possible reaction mechanism involved the interaction between this type of substrate and the catalyst, and the strong electron-donating property of the methoxy group may have reduced the catalytic activity of the catalyst.

### 2.3. Scaled-Up Synthesis

**Scheme 3:**
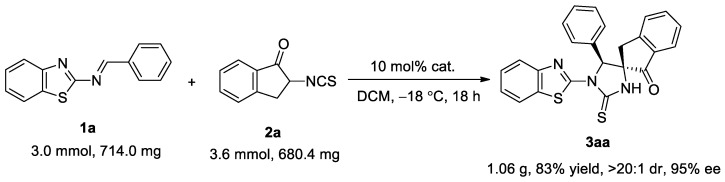
Scaled-up synthesis of **3aa**.

To demonstrate the synthetic applicability of this asymmetric Mannich/cyclization reaction strategy, a gram-scale preparation reaction was carried out under the optimized conditions selected. The experimental results indicated that the reaction could still proceed smoothly when scaled up to the gram level, while maintaining a high yield and excellent stereoselectivity (83% yield, >20:1 dr, 95% ee) (Scheme 3). This effectively validated the potential application value of this strategy in the large-scale asymmetric synthesis of such benzothiazolimine derivatives.

### 2.4. X-ray Diffraction Analysis

**Figure 3:**
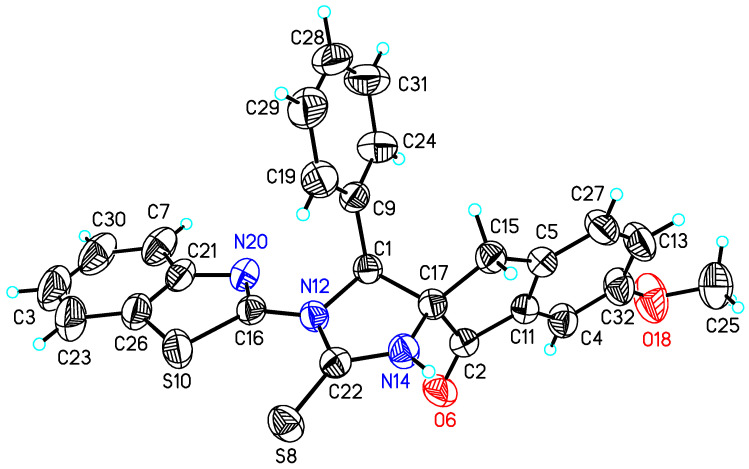
X-ray crystal structure of **3ab**.

To determine the absolute configuration of the Mannich/cyclization reaction product, single crystals of compound **3ab** were obtained through recrystallization from a mixture of ethyl acetate and petroleum ether (2:1). Utilizing single-crystal X-ray diffraction analysis, the crystal data and structural refinement were obtained, which confirmed the absolute configuration of **3ab** to be (2′*R*, 5*S*) (Figure 3) [26] (see Supplementary Materials). The absolute configurations of the other products were assigned by analogy.

### 2.5. Plausible Mechanism

**Scheme 4:**
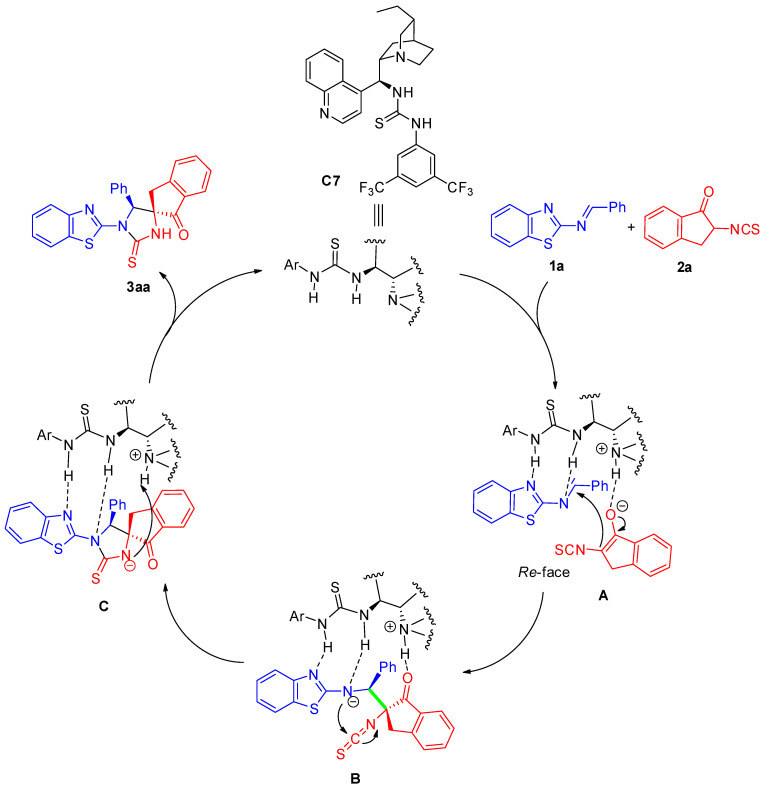
Proposed reaction mechanism.

Based on the absolute configuration of **3ab**, a reasonable asymmetric catalytic reaction mechanism was proposed to better understand this asymmetric Mannich/cyclization reaction (Scheme 4). Catalyst **C7** enhanced the electrophilicity of (*E*)-*N*-(benzo[*d*]thiazol-2-yl)-1-phenylimine (**1a**) and activated 2-isothiocyano-1-indenone (**2a**) through hydrogen bonding. The tertiary amine portion in the catalyst deprotonated **2a** and combined with thiourea amine salts through hydrogen bonding and ion pair interactions to form a transition state **A**. Subsequently, the C-2 of substrate **2a** attacked substrate **1a** through the Re surface and underwent Mannich reaction. Then, intramolecular cyclization addition was carried out through intermediate **B** to obtain intermediate **C**. Finally, the anionic intermediate **C** was protonated to produce the desired product **3aa**, while simultaneously releasing catalyst **C7** into the next catalytic cycle.

## 3. Conclusions

In summary, we have developed an efficient and practical Mannich/cyclization reaction method for the asymmetric synthesis of spirocyclic benzothiazolimine derivatives. This reaction afforded chiral spiro heterocycles with two consecutive stereocenters in high yields (up to 90%) with excellent stereoselectivities (up to >20:1 dr and 98% ee), while integrating two privileged scaffolds beneficial for drug discovery. Meanwhile, the gram-scale preparation was also carried out with retained yield and stereoselectivity.

## 4. Materials and Methods

### 4.1. General Information

Chemical reagents were purchased from commercial sources and used without further purification unless mentioned otherwise. Reactions were monitored by thin-layer chromatography (TLC). Column chromatography separation was performed using 200~300 mesh silica gel. Melting points of solids were determined with a WRX-4 melting-point apparatus (Shanghai YiCe Apparatus & Equipment Co., Ltd., Shanghai, China). Enantiomeric excesses were determined using chiral HPLC analysis on an Agilent 1200 LC instrument (Beijing, China) with a Daicel Chiralpak IA, ADH or IC column. ^1^H NMR spectra were measured with a Bruker Ascend 400 MHz spectrometer (Karlsurhe, Germany), and chemical shifts (δ, ppm) were reported relative to tetramethyl silane (TMS) as the internal standard. ^13^C NMR spectra were measured at 100 MHz with a 400 MHz spectrometer, and chemical shifts are reported as ppm referenced to solvent residue peak (CDCl_3_, δC = 77.00; DMSO-*d*_6_, δC = 39.43). High-resolution mass spectra (EESI) were measured using an Agilent 6520 Accurate-Mass Q-TOF MS system (Beijing, China). Optical rotations were measured with a Krüss P8000 polarimeter (Beijing, China), and data are reported as follows: ${\left[\alpha \right]}_{D}^{25}$ (*c* g/100 mL, solvent). 

### 4.2. Materials

Compounds **1a**–**1n** were prepared according to the literature, as reported by Ni et al. [18], and **2a**–**2h** were prepared according to [27]. The chiral organocatalysts were prepared following the procedures reported in [28,29].

### 4.3. Procedure for the Asymmetric Synthesis of Compound **3**

To a dried small glass bottle were added 2-benzothiazolimine **1** (0.10 mmol), 2-isothiocyanato-1-indanone **2** (0.12 mmol), and the chiral organocatalyst **C7** (5.65 mg, 10%mmol), and then dissolved in DCM (1.0 mL). The mixture was stirred at −18 °C for 12–24 h. After completion of the reaction, the reaction mixture was concentrated and directly purified by flash column chromatography on silica gel (petroleum ether/ethyl acetate = 2:1) to afford the pure product **3** as a white solid. Racemates were prepared following a similar procedure with Et_3_N (20 mol%).

*(2′R,5S)-1-(Benzo[d]thiazol-2-yl)-5-phenyl-2-thioxospiro[imidazolidine-4,2′-inden]-1′(3′H)-one* (**3aa**). Initially, **1a** (23.8 mg, 0.10 mmol) and **2a** (22.7 mg, 0.12 mmol) were purified by silica gel (200–300 mesh) column chromatography using petroleum ether/ethyl acetate (2/1) as eluent to obtain 31.2 mg (79% yield) of compound **3aa** as a white solid, m.p. 239–241 °C. HPLC (Daicel Chiralpak IA, *n*-hexane/isopropanol = 70:30, flow rate 1.0 mL/min, detection at 254 nm): major diastereomer: *t*_R_ = 17.1 min (major enantiomer), *t*_R_ = 8.8 min (minor enantiomer); 98% *ee*. ${\left[\alpha \right]}_{D}^{25}$ = −449.0 (*c* = 0.40, CH_2_Cl_2_). ^1^H NMR (400 MHz, CDCl_3_): δ 7.82 (d, *J* = 7.6 Hz, 1H, ArH), 7.70 (d, *J* = 7.6 Hz, 1H, ArH), 7.62 (t, *J* = 7.2 Hz, 1H, ArH), 7.54 (d, *J* = 8.0 Hz, 1H, ArH), 7.44 (t, *J* = 7.2 Hz, 1H, ArH), 7.38–7.29 (m, 4H, ArH), 7.26 (d, *J* = 8.0 Hz, 2H, ArH), 7.24–7.18 (m, 4H, ArH + NH), 6.10 (s, 1H, CH), 3.00 (dd, *J*_1_ = 17.8 Hz, *J*_2_ = 7.6 Hz, 1H, CH_2_), 2.85 (dd, *J* = 17.8 Hz, 1H, CH_2_) ppm. ^13^C NMR (100 MHz, CDCl_3_): δ 201.0, 179.7, 157.5, 149.9, 148.2, 136.4, 136.1, 132.7, 132.5, 129.0, 128.9, 128.7, 127.1, 126.3, 125.7, 125.6, 123.8, 121.2, 120.9, 70.6, 70.3, 34.9 ppm. HRMS (ESI): *m*/*z* calcd. for C_24_H_18_N_3_OS_2_ [M + H]^+^ 428.0886, found 428.0890.
*(2′R,5S)-1-(Benzo[d]thiazol-2-yl)-6′-methoxy-5-phenyl-2-thioxospiro[imidazolidine-4,2′-inden]-1′(3′H)-one* (**3ab**). Initially, **1a** (23.8 mg, 0.10 mmol) and **2b** (26.3mg, 0.12 mmol) were purified by silica gel (200–300 mesh) column chromatography using petroleum ether/ethyl acetate (2/1) as eluent to obtain 30.8 mg (77% yield) of compound **3ab** as a white solid, m.p. 246–248 °C. HPLC (Daicel Chiralpak ADH, *n*-hexane/isopropanol = 80:20, flow rate 1.0 mL/min, detection at 254 nm): major diastereomer: *t*_R_ = 11.6 min (major enantiomer), *t*_R_ = 10.2 min (minor enantiomer); 53% *ee*. ${\left[\alpha \right]}_{D}^{25}$ = −998.4 (*c* = 0.28, CH_2_Cl_2_). ^1^H NMR (400 MHz, CDCl_3_): δ 7.73 (d, *J* = 7.2 Hz, 1H, ArH), 7.56 (d, *J* = 8.0 Hz, 1H, ArH), 7.36–7.27 (m, 4H, ArH), 7.25–7.19 (m, 5H, ArH), 7.17 (d, *J* = 8.4 Hz, 1H, ArH), 6.84 (s, 1H, NH), 6.11 (s, 1H, CH), 3.84 (s, 3H, CH_3_), 2.87 (d, *J* = 17.6 Hz, 1H, CH_2_), 2.78 (d, *J* = 17.6 Hz, 1H, CH_2_) ppm. ^13^C NMR (100 MHz, CDCl_3_) δ 200.7, 169.3, 166.2, 160.1, 151.5, 143.6, 134.7, 133.2, 131.2, 130.2, 129.3, 129.0, 128.8, 127.1, 126.4, 125.9, 125.7, 122.2, 120.9, 118.7, 105.8, 89.4, 55.7, 35.3 ppm. HRMS (ESI): *m*/*z* calcd. for C_25_H_20_N_3_O_2_S_2_ [M + H]^+^ 458.0991, found 458.0953.
*(2′R,5S)-1-(Benzo[d]thiazol-2-yl)-5′-bromo-5-phenyl-2-thioxospiro[imidazolidine-4,2′-inden]-1′(3′H)-one* (**3ac**). Initially, **1a** (23.8 mg, 0.10 mmol) and **2c** (31.9 mg, 0.12 mmol) were purified by silica gel (200–300 mesh) column chromatography using petroleum ether/ethyl acetate (2/1) as eluent to obtain 27.9 mg (72% yield) of compound **3ac** as a white solid, m.p. 243–245 °C. HPLC (Daicel Chiralpak ADH, *n*-hexane/isopropanol = 80:20, flow rate 1.0 mL/min, detection at 254 nm): major diastereomer: *t*_R_ = 9.8 min (major enantiomer), *t*_R_ = 12.5 min (minor enantiomer); 70% *ee*. ${\left[\alpha \right]}_{D}^{25}$ = −238.7 (*c* = 0.15, CH_2_Cl_2_). ^1^H NMR (400 MHz, CDCl_3_): δ 7.86 (d, *J* = 7.6 Hz, 1H, ArH), 7.73 (d, = 7.6 Hz, 1H, ArH), 7.65 (td, *J* = 7.6, 1.2 Hz, 1H, ArH), 7.56 (d, *J* = 8.0 Hz, 1H, ArH), 7.47 (t, *J* = 7.6 Hz, 1H, ArH), 7.38–7.29 (m, 4H, ArH), 7.25–7.19 (m, 3H, ArH), 6.83 (s, 1H, NH), 6.12 (s, 1H, CH), 2.95 (d, *J* = 17.8 Hz, 1H, CH_2_), 2.87 (d, *J* = 17.8 Hz, 1H, CH_2_) ppm. ^13^C NMR (100 MHz, CDCl_3_): δ 201.0, 179.8, 157.5, 149.9, 148.3, 136.4, 136.1, 132.8, 132.6, 130.9, 129.0, 128.9, 128.7, 126.3, 125.7, 125.6, 123.8, 122.3, 121.2, 120.9, 70.6, 70.3, 34.9 ppm. HRMS (ESI): *m*/*z* calcd. for C_24_H_17_N_3_O_3_S_2_ [M-Br]^+^ 428.0886, found 428.0853.
*(2′R,5S)-1-(Benzo[d]thiazol-2-yl)-6′-bromo-5-phenyl-2-thioxospiro[imidazolidine-4,2′-inden]-1′(3′H)-one* (**3ad**)**.** Initially, **1a** (23.8 mg, 0.10 mmol) and **2d** (31.9mg, 0.12 mmol) were purified by silica gel (200–300 mesh) column chromatography using petroleum ether/ethyl acetate (2/1) as eluent to obtain 28.0mg (72% yield) of compound **3ad** as a white solid, m.p. 257–259 °C. HPLC (Daicel Chiralpak IA, *n*-hexane/isopropanol = 70:30, flow rate 1.0 mL/min, detection at 254 nm): major diastereomer: *t*_R_ = 15.6 min (major enantiomer), *t*_R_ = 8.3 min (minor enantiomer); 94% *ee*. ${\left[\alpha \right]}_{D}^{25}$ = −776.2 (*c* = 0.50, CH_2_Cl_2_). ^1^H NMR (400 MHz, CDCl_3_): δ 7.81 (d, *J* = 7.6 Hz, 1H, ArH), 7.70 (d, *J* = 8.0 Hz, 1H, ArH), 7.61 (t, *J* = 7.6 Hz, 1H, ArH), 7.54 (d, *J* = 8.0 Hz, 1H, ArH), 7.42 (t, *J* = 7.4 Hz, 1H, ArH), 7.35–7.29 (m, 4H, ArH), 7.27–7.18 (m, 4H, ArH + NH), 6.10 (s, 1H, CH), 2.98 (d, *J* = 17.8 Hz, 1H, CH_2_), 2.84 (d, *J* = 17.8 Hz, 1H, CH_2_) ppm. ^13^C NMR (176 MHz, CDCl_3_) δ 201.1, 179.6, 157.5, 149.9, 148.2, 136.4, 136.0, 132.7, 132.5, 129.0, 128.9, 128.6, 126.3, 125.64, 125.57, 123.7, 121.1, 120.8, 70.7, 70.2, 34.9 ppm. HRMS (ESI): *m*/*z* calcd. for C_24_H_17_BrN_3_O_3_S_2_ [M + H]^+^ 505.9991, found 505.9969.
*(2′R,5S)-1-(Benzo[d]thiazol-2-yl)-6′-fluoro-5-phenyl-2-thioxospiro[imidazolidine-4,2′-inden]-1′(3′H)-one* (**3ae**). Initially, **1a** (23.8 mg, 0.10 mmol) and **2e** (24.8 mg, 0.12 mmol) were purified by silica gel (200–300 mesh) column chromatography using petroleum ether/ethyl acetate (2/1) as eluent to obtain 29.5 mg (73% yield) of compound **3ae** as a white solid, m.p. 277–279 °C. HPLC (Daicel Chiralpak ADH, *n*-hexane/isopropanol = 70:30, flow rate 1.0 mL/min, detection at 254 nm): major diastereomer: *t*_R_ = 24.0 min (major enantiomer), *t*_R_ = 14.7 min (minor enantiomer); 84% *ee*. ${\left[\alpha \right]}_{D}^{25}$ = −418.6 (*c* = 0.55, CH_2_Cl_2_). ^1^H NMR (400 MHz, CDCl_3_): δ 7.73 (d, *J* = 8.0 Hz, 1H, ArH), 7.56 (d, *J* = 8.0 Hz, 1H, ArH), 7.47 (dd, *J* = 7.0, 2.6 Hz, 1H, ArH), 7.39–7.31 (m, 4H, ArH), 7.30–7.26 (m, 2H, ArH), 7.24–7.20 (m, 3H, ArH), 6.99 (s, 1H, NH), 6.11 (s, 1H, CH), 2.92 (d, *J* = 17.6 Hz, 1H, CH_2_), 2.83 (d, *J* = 18.0 Hz, 1H, CH_2_) ppm. ^13^C NMR (100 MHz, CDCl_3_) δ 200.4, 179.6, 162.7 (^1^*J*_C–F_ = 249.0 Hz), 157.4, 148.1, 145.4, 135.9, 134.2 (^3^*J*_C–F_ = 7.4 Hz), 132.6, 129.1 (^4^*J*_C–F_ = 5.5 Hz), 127.8 (^3^*J*_C–F_ = 7.9 Hz), 125.7, 124.2 (^2^*J*_C–F_ = 23.5 Hz), 123.8, 121.2, 120.9, 111.3 (^2^*J*_C–F_ = 22.0 Hz), 77.2, 71.3, 70.2, 34.4 ppm. HRMS (ESI): *m*/*z* calcd. for C_24_H_17_FN_3_OS_2_ [M + H]^+^ 446.0792, found 446.0763.
*(2′R,5S)-1-(Benzo[d]thiazol-2-yl)-6′-methyl-5-phenyl-2-thioxospiro[imidazolidine-4,2′-inden]-1′(3′H)-one* (**3af**). Initially, **1a** (23.8 mg, 0.10 mmol) and **2f** (24.4 mg, 0.12 mmol) were purified by silica gel (200–300 mesh) column chromatography using petroleum ether/ethyl acetate (2/1) as eluent to obtain 31.5 mg (78% yield) of compound **3af** as a white solid, m.p. 249–251 °C. HPLC (Daicel Chiralpak ADH, *n*-hexane/isopropanol = 70:30, flow rate 1.0 mL/min, detection at 254 nm): major diastereomer: *t*_R_ = 18.6 min (major enantiomer), *t*_R_ = 12.8 min (minor enantiomer); 90% *ee*. ${\left[\alpha \right]}_{D}^{25}$ = −330.0 (*c* = 0.33, CH_2_Cl_2_). ^1^H NMR (400 MHz, CDCl_3_): δ 7.73 (d, *J* = 8.0 Hz, 1H, ArH), 7.64 (s, 1H, ArH), 7.55 (d, *J* = 8.0 Hz, 1H, ArH), 7.46 (d, *J* = 7.6 Hz, 1H, ArH), 7.36–7.27 (m, 4H, ArH), 7.23–7.15 (m, 4H, ArH), 6.69 (s, 1H, NH), 6.11 (s, 1H, CH), 2.88 (d, *J* = 17.6 Hz, 1H, CH_2_), 2.80 (d, *J* = 17.6 Hz, 1H, CH_2_), 2.43 (s, 3H, CH_3_) ppm. ^13^C NMR (100 MHz, CDCl_3_): δ 201.0, 179.7, 157.5, 148.3, 147.2, 138.9, 137.7, 136.2, 132.7, 132.6, 129.0, 128.9, 126.0, 125.6, 125.4, 123.7, 121.2, 120.9, 71.0, 70.3, 34.6, 21.1 ppm. HRMS (ESI): *m*/*z* calcd. for C_25_H_20_N_3_OS_2_ [M + H]^+^ 442.1042, found 442.1008.
*(2′R,5S)-1-(Benzo[d]thiazol-2-yl)-6′-chloro-5-phenyl-2-thioxospiro[imidazolidine-4,2′-inden]-1′(3′H)-one* (**3ag**). Initially, **1a** (23.8 mg, 0.10 mmol) and **2g** (26.8 mg, 0.12 mmol) were purified by silica gel (200–300 mesh) column chromatography using petroleum ether/ethyl acetate (2/1) as eluent to obtain 26.9 mg (69% yield) of compound **3ag** as a white solid, m.p. 239–241 °C. HPLC (Daicel Chiralpak IA, *n*-hexane/isopropanol = 70:30, flow rate 1.0 mL/min, detection at 254 nm): major diastereomer: *t*_R_ = 15.7 min (major enantiomer), *t*_R_ = 8.3 min (minor enantiomer); 21% *ee*. ${\left[\alpha \right]}_{D}^{25}$ = −576.9 (*c* = 0.35, CH_2_Cl_2_). ^1^H NMR (400 MHz, CDCl_3_) δ 7.85 (d, *J* = 7.6 Hz, 1H, ArH), 7.72 (d, *J* = 7.6 Hz, 1H, ArH), 7.65 (t, *J* = 7.6 Hz, 1H, ArH), 7.55 (d, *J* = 8.0 Hz, 1H, ArH), 7.46 (t, *J* = 7.6 Hz, 1H, ArH), 7.37–7.29 (m, 4H, ArH), 7.23–7.19 (m, 3H, ArH), 7.07 (s, 1H, NH), 6.11 (s, 1H, CH), 2.97 (d, *J* = 17.6 Hz, 1H, CH_2_), 2.86 (d, *J* = 17.6 Hz, 1H, CH_2_) ppm. ^13^C NMR (100 MHz, CDCl_3_): δ 200.9, 179.7, 157.4, 149.8, 148.2, 136.4, 136.1, 132.7, 132.6, 129.0, 128.9, 128.8, 126.4, 125.7, 125.6, 123.8, 121.2, 120.9, 70.6, 70.3, 34.9 ppm. HRMS (ESI): *m*/*z* calcd. for C_24_H_17_ClN_3_OS_2_ [M + H]^+^ 462.0497, found 462.0494.
*(2′R,5S)-1-(Benzo[d]thiazol-2-yl)-5′-methoxy-5-phenyl-2-thioxospiro[imidazolidine-4,2′-inden]-1′(3′H)-one* (**3ah**). Initially, **1a** (23.8 mg, 0.10 mmol) and **2h** (26.3mg, 0.12 mmol) were purified by silica gel (200–300 mesh) column chromatography using petroleum ether/ethyl acetate (2/1) as eluent to obtain 30.8 mg (77% yield) of compound **3ah** as a white solid, m.p. 236–238 °C. HPLC (Daicel Chiralpak IC, *n*-hexane/isopropanol = 80:20, flow rate 1.0 mL/min, detection at 254 nm): major diastereomer: *t*_R_ = 9.04 min (major enantiomer), *t*_R_ = 12.26 min (minor enantiomer); 83% *ee*. ${\left[\alpha \right]}_{D}^{25}$ = −94.2 (*c* = 0.55, CH_2_Cl_2_). ^1^H NMR (400 MHz, CDCl_3_): δ 7.72 (d, *J* = 8.0 Hz, 1H, ArH), 7.55 (d, *J* = 8.0 Hz, 1H, ArH), 7.36–7.27 (m, 4H, ArH), 7.22 (d, *J* = 7.8 Hz, 5H, ArH), 7.17 (d, *J* = 7.8 Hz, 1H, ArH), 7.06 (s, 1H, NH), 6.10 (s, 1H, CH), 3.83 (s, 3H, CH_3_), 2.88 (d, *J* = 17.4 Hz, 1H, CH_2_), 2.77 (d, *J* = 17.4 Hz, 1H, CH_2_) ppm. ^13^C NMR (100 MHz, CDCl_3_): δ 200.9, 179.8, 160.3, 157.5, 148.3, 142.7, 136.2, 133.7, 132.8, 129.0, 128.4, 127.1, 125.9, 125.7, 123.8, 121.2, 120.9, 106.5, 71.3, 70.4, 55.8, 34.4 ppm. HRMS (ESI): *m*/*z* calcd. for C_25_H_20_N_3_O_2_S_2_ [M + H]^+^ 458.0992, found 458.0959.
*(2′R,5S)-1-(6-Chlorobenzo[d]thiazol-2-yl)-5-phenyl-2-thioxospiro[imidazolidine-4,2′-inden]-1′(3′H)-one* (**3ba**). Initially, **1b** (27.2 mg, 0.10 mmol) and **2a** (22.7mg, 0.12 mmol) were purified by silica gel (200–300 mesh) column chromatography using petroleum ether/ethyl acetate (2/1) as eluent to obtain 30.2 mg (79% yield) of compound **3ba** as a white solid, m.p. 243–245 °C. HPLC (Daicel Chiralpak IC, *n*-hexane/isopropanol = 90:10, flow rate 1.0 mL/min, detection at 254 nm): major diastereomer: *t*_R_ = 25.3 min (major enantiomer), *t*_R_ = 48.9 min (minor enantiomer); 40% *ee*. ${\left[\alpha \right]}_{D}^{25}$ = −94.2 (*c* = 0.55, CH_2_Cl_2_). ^1^H NMR (400 MHz, CDCl_3_): δ 7.85 (d, *J* = 8.0 Hz, 1H, ArH), 7.68–7.63 (m, 2H, ArH), 7.49–7.44 m, 2H, ArH), 7.38–7.32 (m, 3H, ArH), 7.29 (d, *J* = 7.6 Hz, 1H, ArH), 7.25–7.20 (m, 3H, ArH), 6.90 (s, 1H, NH), 6.07 (s, 1H, CH), 2.96 (d, *J* = 18.0 Hz, 1H, CH_2_), 2.87 (d, *J* = 18.0 Hz, 1H, CH_2_) ppm. ^13^C NMR (100 MHz, CDCl_3_): δ 200.9, 179.7, 157.8, 149.9, 146.9, 136.6, 135.9, 133.9, 132.5, 129.14, 129.11, 129.0, 128.8, 126.4, 126.3, 125.7, 122.0, 120.4, 70.6, 70.3, 34.9 ppm. HRMS (ESI): *m*/*z* calcd. for C_24_H_17_ClN_3_OS_2_ [M + H]^+^ 462.0497, found 462.0459.
*(2′R,5S)-1-(6-Methoxybenzo[d]thiazol-2-yl)-5-phenyl-2-thioxospiro[imidazolidine-4,2′-inden]-1′(3′H)-one* (**3ca**). Initially, **1c** (26.8 mg, 0.10 mmol) and **2a** (22.7 mg, 0.12 mmol) were purified by silica gel (200–300 mesh) column chromatography using petroleum ether/ethyl acetate (2/1) as eluent to obtain 37.9 mg (83% yield) of compound **3ca** as a white solid, m.p. 257–259 °C. HPLC (Daicel Chiralpak IC, *n*-hexane/isopropanol = 80:20, flow rate 1.0 mL/min, detection at 254 nm): major diastereomer: *t*_R_ = 12.2 min (major enantiomer), *t*_R_ = 20.1 min (minor enantiomer); 97% *ee*. ${\left[\alpha \right]}_{D}^{25}$ = −94.7 (*c* = 0.48, CH_2_Cl_2_). ^1^H NMR (400 MHz, CDCl_3_): δ 7.82 (d, *J* = 7.6 Hz, 1H, ArH), 7.74–7.45 (m, 2H, ArH), 7.46–7.41 (m, 2H, ArH), 7.33–7.27 (m, 3H, ArH + NH), 7.22–7.13 (m, 4H, ArH), 6.87 (dd, *J* = 8.8, 2.8 Hz, 1H, ArH), 6.06 (s, 1H, CH), 3.81 (s, 3H, CH_3_), 2.96 (d, *J* = 18.0 Hz, 1H, CH_2_), 2.83 (d, *J* = 18.0 Hz, 1H, CH_2_) ppm. ^13^C NMR (100 MHz, CDCl_3_): δ 200.9, 179.6, 156.7, 155.5, 149.8, 142.6, 136.4, 136.1, 134.0, 132.6, 129.0, 128.9, 128.7, 126.3, 125.6, 121.8, 114.6, 103.8, 70.6, 70.3, 55.8, 35.0 ppm. HRMS (ESI): *m*/*z* calcd. for C_25_H_20_N_3_O_2_S_2_ [M + H]^+^ 458.0991, found 458.0961.
*(2′R,5S)-1-(Benzo[d]thiazol-2-yl)-5-(3-methoxyphenyl)-2-thioxospiro[imidazolidine-4,2′-inden]-1′(3′H)-one* (**3da**). Initially, **1d** (26.8 mg, 0.10 mmol) and **2a** (22.7 mg, 0.12 mmol) were purified by silica gel (200–300 mesh) column chromatography using petroleum ether/ethyl acetate (2/1) as eluent to obtain 30.5 mg (81% yield) of compound **3da** as a white solid, m.p. 197–199 °C. HPLC (Daicel Chiralpak IIC, *n*-hexane/isopropanol = 80:20, flow rate 1.0 mL/min, detection at 254 nm): major diastereomer: *t*_R_ = 7.5 min (major enantiomer), *t*_R_ = 9.4 min (minor enantiomer); 8% *ee*. ${\left[\alpha \right]}_{D}^{25}$ = −27.1 (*c* = 0.70, CH_2_Cl_2_). ^1^H NMR (400 MHz, CDCl_3_): δ 7.86 (d, *J* = 8.0 Hz, 1H), 7.74 (d, *J* = 7.6 Hz, 1H, ArH), 7.68–7.63 (m, 1H, ArH), 7.58 (d, *J* = 8.0 Hz, 1H, ArH), 7.47 (t, *J* = 7.6 Hz, 1H, ArH), 7.32–7.27 (m, 2H, ArH), 7.25–7.20 (m, 2H, ArH), 6.85–6.80 (m, 2H, ArH), 6.74 (t, *J* = 2.0 Hz, 1H, ArH), 6.66 (s, 1H, NH), 6.08 (s, 1H, CH), 3.68 (s, 3H, CH_3_), 2.96 (d, *J* = 18.0 Hz, 1H, CH_2_), 2.96 (d, *J* = 18.0 Hz, 1H, CH_2_), 2.91 (d, *J* = 18.0 Hz, 1H, CH_2_) ppm. ^13^C NMR (100 MHz, CDCl_3_): δ 201.0, 179.6, 160.0, 157.5, 150.0, 148.3, 137.6, 136.4, 132.8, 132.5, 130.2, 128.7, 126.4, 125.64, 125.60, 123.8, 121.2, 120.9, 114.2, 70.6, 70.2, 55.2, 34.9 ppm. HRMS (ESI): *m*/*z* calcd. for C_25_H_20_N_3_O_2_S_2_ [M + H]^+^ 458.0991, found 458.0956.
*(2′R,5S)-1-(Benzo[d]thiazol-2-yl)-5-(4-methylphenyl)-2-thioxospiro[imidazolidine-4,2′-inden]-1′(3′H)-one* (**3ea**). Initially, **1e** (25.2 mg, 0.10 mmol) and **2a** (22.7 mg, 0.12 mmol) were purified by silica gel (200–300 mesh) column chromatography using petroleum ether/ethyl acetate (2/1) as eluent to obtain 32.0 mg (85% yield) of compound **3ea** as a white solid, m.p. 234–236 °C. HPLC (Daicel Chiralpak IC, *n*-hexane/isopropanol = 80:20, flow rate 1.0 mL/min, detection at 254 nm): major diastereomer: *t*_R_ = 7.0 min (major enantiomer), *t*_R_ = 9.3 min (minor enantiomer); 20% *ee*. ${\left[\alpha \right]}_{D}^{25}$ = −52.8 (*c* = 0.30, CH_2_Cl_2_). ^1^H NMR (400 MHz, CDCl_3_): δ 7.85 (d, *J* = 7.6 Hz, 1H, ArH), 7.73 (d, *J* = 8.0 Hz, 1H, ArH), 7.65 (t, *J* = 7.6 Hz, 1H, ArH), 7.57 (d, *J* = 8.0 Hz, 1H, ArH), 7.47 (t, *J* = 7.6 Hz, 1H, ArH), 7.31–7.26 (m, 3H, ArH), 7.23–7.19 (m, 1H, ArH), 7.14–7.10 (m, 3H, ArH), 6.67 (s, 1H, NH), 6.09 (s, 1H, CH), 2.95 (d, *J* = 18.0 Hz, 1H, CH_2_), 2.90 (d, *J* = 17.6 Hz, 1H, CH_2_), 2.31 (s, 3H, CH_3_) ppm. ^13^C NMR (100 MHz, CDCl_3_): δ 201.0, 179.8, 157.5, 149.9, 148.3, 138.8, 136.4, 133.1, 132.8, 132.6, 129.7, 128.7, 126.4, 125.6, 123.7, 121.2, 120.9, 70.7, 70.2, 34.9, 21.2 ppm. HRMS (ESI): *m*/*z* calcd. for C_25_H_20_N_3_OS_2_ [M + H]^+^ 442.1042, found 442.1007.
*(2′R,5S)-1-(Benzo[d]thiazol-2-yl)-5-(2-fluorophenyl)-2-thioxospiro[imidazolidine-4,2′-inden]-1′(3′H)-one* (**3fa**). Initially, **1f** (25.6 mg, 0.10 mmol) and **2a** (22.7 mg, 0.12 mmol) were purified by silica gel (200–300 mesh) column chromatography using petroleum ether/ethyl acetate (2/1) as eluent to obtain 27.9mg (73% yield) of compound **3fa** as a white solid, m.p. 228–220 °C. HPLC (Daicel Chiralpak IC, *n*-hexane/isopropanol = 80:20, flow rate 1.0 mL/min, detection at 254 nm): major diastereomer: *t*_R_ = 6.9 min (major enantiomer), *t*_R_ = 7.8 min (minor enantiomer); 88% *ee*. ${\left[\alpha \right]}_{D}^{25}$ = −321.5 (*c* = 0.80, CH_2_Cl_2_). ^1^H NMR (400 MHz, CDCl_3_): δ 7.82 (d, *J* = 7.6 Hz, 1H, ArH), 7.73 (d, *J* = 8.0 Hz, 1H, ArH), 7.63 (t, *J* = 7.6 Hz, 1H, ArH), 7.57 (d, *J* = 8.0 Hz, 1H, ArH), 7.43 (d, *J* = 7.6 Hz, 1H, ArH), 7.32–7.27 (m, 3H, ArH), 7.24–7.15 (m, 3H, ArH + NH), 7.11 (d, *J* = 7.2 Hz, 1H, ArH), 7.07 (d, *J* = 8.8 Hz, 1H, ArH), 6.53 (s, 1H, CH), 3.03 (d, *J* = 18.0 Hz, 1H, CH_2_), 2.96 (d, *J* = 18.0 Hz, 1H, CH_2_) ppm. ^13^C NMR (100 MHz, CDCl_3_): δ 200.7, 179.5, 160.4 (^1^*J*_C–F_ = 246.3 Hz), 157.0, 150.0, 148.2, 136.5, 132.7, 132.3, 130.4 (^3^*J*_C–F_ = 8.0 Hz), 128.6, 126.8, 126.2, 125.7, 125.6, 125.1 (^4^*J*_C–F_ = 3.2 Hz), 123.8, 123.4 (^2^*J*_C–F_ = 13.4 Hz), 121.3, 120.8, 115.5 (^2^*J*_C–F_ = 21.1 Hz), 70.2, 63.3, 34.8 ppm. HRMS (ESI): *m*/*z* calcd. for C_24_H_17_FN_3_OS_2_ [M + H]^+^ 446.0792, found 446.0759.
*(2′R,5S)-1-(Benzo[d]thiazol-2-yl)-5-(2-bromophenyl)-2-thioxospiro[imidazolidine-4,2′-inden]-1′(3′H)-one* (**3ga**). Initially, **1g** (31.6 mg, 0.10 mmol) and **2a** (22.7 mg, 0.12 mmol) were purified by silica gel (200–300 mesh) column chromatography using petroleum ether/ethyl acetate (2/1) as eluent to obtain 25.8 mg (70% yield) of compound **3ga** as a white solid, m.p. 210–212 °C. HPLC (Daicel Chiralpak IC, *n*-hexane/isopropanol = 95:5, flow rate 1.0 mL/min, detection at 254 nm): major diastereomer: *t*_R_ = 24.6 min (major enantiomer), *t*_R_ = 27.2 min (minor enantiomer); 96% *ee*. ${\left[\alpha \right]}_{D}^{25}$ = −371.4 (*c* = 0.48, CH_2_Cl_2_). ^1^H NMR (400 MHz, CDCl_3_): δ 7.84 (d, *J* = 7.6 Hz, 1H, ArH), 7.73 (d, *J* = 7.6 Hz, 1H, ArH), 7.67–7.62 (m, 1H, ArH), 7.59–7.56 (m, 2H, ArH), 7.45 (t, *J* = 7.6 Hz, 1H, ArH), 7.34–7.27 (m, 3H, ArH), 7.25–7.15 (m, 3H, ArH), 6.91 (s, 1H, NH), 6.66 (s, 1H, CH), 2.96 (ABq, *J* = 18.4 Hz, 2H, CH_2_) ppm. ^13^C NMR (100 MHz, CDCl_3_): δ 200.7, 179.3, 156.9, 150.6, 148.3, 136.5, 135.6, 133.0, 132.7, 132.4, 130.1, 128.7, 128.4, 127.0, 126.3, 125.7, 125.5, 124.0, 123.8, 121.5, 120.8, 70.1, 69.0, 35.2 ppm. HRMS (ESI): *m*/*z* calcd. for C_24_H_17_^79^BrN_3_OS_2_ [M + H]^+^ 505.9991, found 505.9948; calcd. for C_24_H_17_^81^BrN_3_OS_2_ [M + H]^+^ 507.9971, found 507.9930.
*(2′R,5S)-1-(Benzo[d]thiazol-2-yl)-5-(3-methylphenyl)-2-thioxospiro[imidazolidine-4,2′-inden]-1′(3′H)-one* (**3ha**). Initially, **1h** (25.2 mg, 0.10 mmol) and **2a** (22.7 mg, 0.12 mmol) were purified by silica gel (200–300 mesh) column chromatography using petroleum ether/ethyl acetate (2/1) as eluent to obtain 29.8 mg (80% yield) of compound **3ha** as a white solid, m.p. 167–169 °C. HPLC (Daicel Chiralpak IC, *n*-hexane/isopropanol = 80:20, flow rate 1.0 mL/min, detection at 254 nm): major diastereomer: *t*_R_ = 7.6 min (major enantiomer), *t*_R_ = 10.3 min (minor enantiomer); 50% *ee*. ${\left[\alpha \right]}_{D}^{25}$ = −128.5 (*c* = 1.25, CH_2_Cl_2_). ^1^H NMR (400 MHz, CDCl_3_) δ 7.81 (t, *J* = 8.2 Hz, 1H, ArH), 7.70 (d, *J* = 8.0 Hz, 1H, ArH), 7.63–7.54 (m, 2H, ArH), 7.45–7.39 (m, 2H, ArH), 7.29–7.26 (m, 2H, ArH), 7.22–7.17 (m, 2H, ArH), 7.10 (d, *J* = 7.6 Hz, 1H, ArH), 7.04 (d, *J* = 6.8 Hz, 2H, ArH + NH), 6.09 (s, 1H, CH), 2.98 (dd, *J* = 17.8, 7.2 Hz, 1H, CH_2_), 2.84 (d, *J* = 17.6 Hz, 1H, CH_2_), 2.30 (s, 3H, CH_3_) ppm. ^13^C NMR (100 MHz, CDCl_3_) δ 201.3, 179.5, 157.5, 150.1, 148.2, 138.8, 136.3, 135.9, 132.7, 132.4, 129.7, 128.8, 128.6, 128.5, 126.3, 126.2, 125.6, 125.5, 123.6, 121.14, 121.12, 120.8, 70.7, 70.3, 34.8, 21.4 ppm. HRMS (ESI): *m*/*z* calcd. for C_25_H_20_N_3_OS_2_ [M + H]^+^ 442.1043, found 442.1010.
*(2′R,5S)-1-(Benzo[d]thiazol-2-yl)-5-(3-bromophenyl)-2-thioxospiro[imidazolidine-4,2′-inden]-1′(3′H)-one* (**3ia**). Initially, **1i** (31.6 mg, 0.10 mmol) and **2a** (22.7 mg, 0.12 mmol) were purified by silica gel (200–300 mesh) column chromatography using petroleum ether/ethyl acetate (2/1) as eluent to obtain 29.6 mg (73% yield) of compound **3ia** as a white solid, m.p. 134–136 °C. HPLC (Daicel Chiralpak IC, *n*-hexane/isopropanol = 80:20, flow rate 1.0 mL/min, detection at 254 nm): major diastereomer: *t*_R_ = 6.9 min (major enantiomer), *t*_R_ = 8.8 min (minor enantiomer); 20% *ee*. ${\left[\alpha \right]}_{D}^{25}$ = −8.1 (*c* = 0.15, CH_2_Cl_2_). ^1^H NMR (400 MHz, CDCl_3_): δ 7.86–7.83 (m, 1H, ArH), 7.73 (d, *J* = 8.0 Hz, 1H, ArH), 7.86–7.64 (m, 1H, ArH), 7.58 (d, *J* = 8.0 Hz, 1H, ArH), 7.47 (t, *J* = 7.8 Hz, 1H, ArH), 7.39 (s, 1H, ArH), 7.31 (t, *J* = 7.4 Hz, 2H, ArH), 7.24–7.18 (m, 2H, ArH), 7.00 (s, 1H, NH), 6.06 (s, 1H, CH), 3.01 (d, *J* = 17.6 Hz, 1H, CH_2_) 2.88 (d, *J* = 18.0 Hz, 1H, CH_2_) ppm. ^13^C NMR (100 MHz, CDCl_3_): δ 200.7, 179.4, 157.2, 149.8, 148.1, 138.4, 136.6, 136.3, 132.7, 132.3, 132.1, 131.8, 130.6, 128.8, 127.0, 126.4, 125.7, 125.6, 123.9, 121.2, 120.9, 70.5, 69.5, 34.9 ppm. HRMS (ESI): *m*/*z* calcd. for C_24_H_17_^79^BrN_3_OS_2_ [M + H]^+^ 505.9991, found 505.9943; calcd. for C_24_H_17_^81^BrN_3_OS_2_ [M + H]^+^ 507.9971, found 507.9927.
*(2′R,5S)-1-(Benzo[d]thiazol-2-yl)-5-(2-chlorophenyl)-2-thioxospiro[imidazolidine-4,2′-inden]-1′(3′H)-one* (**3ja**). Initially, **1j** (27.2 mg, 0.10 mmol) and **2a** (22.7 mg, 0.12 mmol) were purified by silica gel (200–300 mesh) column chromatography using petroleum ether/ethyl acetate (2/1) as eluent to obtain 26.9 mg (69% yield) of compound **3ja** as a white solid, m.p. 227–229 °C. HPLC (Daicel Chiralpak ADH, *n*-hexane/isopropanol = 80:20, flow rate 1.0 mL/min, detection at 254 nm): major diastereomer: *t*_R_ = 20.1 min (major enantiomer), *t*_R_ = 17.6 min (minor enantiomer); 38% *ee*. ${\left[\alpha \right]}_{D}^{25}$ = −39.4 (*c* = 1.60, CH_2_Cl_2_). ^1^H NMR (400 MHz, CDCl_3_): δ 7.85 (d, *J* = 7.6 Hz, 1H, ArH), 7.75–7.64 (m, 4H, ArH), 7.55–7.46 (m, 2H, ArH), 7.38 (d, *J* = 8.0 Hz, 2H, ArH), 7.32–7.28 (m, 2H, ArH), 7.24 (t, *J* = 7.6 Hz, 1H, ArH), 6.93 (s, 1H, NH), 6.15 (s, 1H, CH), 3.05–2.73 (m, 2H, CH_2_) ppm. ^13^C NMR (100 MHz, CDCl_3_): δ 200.6, 179.5, 157.0, 150.5, 148.3, 136.5, 134.0, 133.8, 132.8, 132.4, 129.8, 129.7, 128.7, 127.9, 126.8, 126.3, 125.7, 125.5, 123.8, 121.5, 120.9, 70.1, 66.7, 35.1 ppm. HRMS (ESI): *m*/*z* calcd. for C_24_H_17_ClN_3_OS_2_ [M + H]^+^ 462.0497, found 462.0461.
*(2′R,5S)-1-(Benzo[d]thiazol-2-yl)-5-(4-methoxyphenyl)-2-thioxospiro[imidazolidine-4,2′-inden]-1′(3′H)-one* (**3ka**). Initially, **1k** (26.8 mg, 0.10 mmol) and **2a** (22.7 mg, 0.12 mmol) were purified by silica gel (200–300 mesh) column chromatography using petroleum ether/ethyl acetate (2/1) as eluent to obtain 32.4 mg (84% yield) of compound **3ka** as a white solid, m.p. 242–245 °C. HPLC (Daicel Chiralpak ADH, *n*-hexane/isopropanol = 80:20, flow rate 1.0 mL/min, detection at 254 nm): major diastereomer: *t*_R_ = 54.9 min (major enantiomer), *t*_R_ = 18.1 min (minor enantiomer); 10% *ee*. ${\left[\alpha \right]}_{D}^{25}$ = −50.4 (*c* = 1.25, CH_2_Cl_2_). ^1^H NMR (400 MHz, CDCl_3_): δ 7.78 (d, *J* = 7.6 Hz, 1H, ArH), 7.69 (d, *J* = 7.6 Hz, 1H, ArH), 7.63–7.54 (m, 3H, ArH), 7.40 (t, *J* = 7.2 Hz, 1H, ArH), 7.30–7.25 (m, 2H, ArH), 7.21–7.14 (m, 3H, ArH), 6.84 (s, 1H, ArH), 6.82 (s, 1H, NH), 6.04 (s, 1H, CH), 3.74 (s, 3H, CH_3_), 3.02 (d, *J* = 17.6 Hz, 1H, CH_2_), 2.89 (d, *J* = 17.6 Hz, 1H, CH_2_) ppm. ^13^C NMR (100 MHz, CDCl_3_): δ 201.3, 179.5, 159.8, 157.5, 150.0, 148.2, 136.3, 132.7, 132.5, 131.1, 128.5, 128.0, 126.3, 125.6, 125.5, 123.7, 121.1, 120.8, 114.3, 70.8, 70.0, 55.2, 34.7 ppm. HRMS (ESI): *m*/*z* calcd. for C_25_H_20_N_3_O_2_S_2_ [M + H]^+^ 458.0991, found 458.0957.
*(2′R,5S)-1-(Benzo[d]thiazol-2-yl)-5-(naphthalen-2-yl)-2-thioxospiro[imidazolidine-4,2′-inden]-1′(3′H)-one* (**3la**). Initially, **1l** (28.8 mg, 0.10 mmol) and **2a** (22.7 mg, 0.12 mmol) were purified by silica gel (200–300 mesh) column chromatography using petroleum ether/ethyl acetate (2/1) as eluent to obtain 33.8 mg (87% yield) of compound **3la** as a white solid, m.p. 162–164 °C. HPLC (Daicel Chiralpak IC, *n*-hexane/isopropanol = 80:20, flow rate 1.0 mL/min, detection at 254 nm): major diastereomer: *t*_R_ = 8.1 min (major enantiomer), *t*_R_ = 11.3 min (minor enantiomer); 92% *ee*. ${\left[\alpha \right]}_{D}^{25}$ = −371.0 (*c* = 0.40, CH_2_Cl_2_). ^1^H NMR (400 MHz, CDCl_3_): δ 7.85–7.78 (m, 4H, ArH), 7.74 (s, 1H, ArH), 7.69 (d, *J* = 7.6 Hz 1H, ArH), 7.62–7.58 (m, 1H, ArH), 7.52–7.42 (m, 4H), 7.35–7.27 (m, 2H, ArH), 7.24–7.14 (m, 3H, ArH), 7.10 (s, 1H, NH), 6.29 (s, 1H, CH), 2.98 (d, *J* = 17.8 Hz, 1H, CH_2_), 2.87 (d, *J* = 17.8 Hz, 1H, CH_2_) ppm. ^13^C NMR (100 MHz, CDCl_3_): δ 201.1, 179.7, 157.5, 149.9, 148.2, 136.4, 133.5, 133.4, 133.1, 132.7, 132.5, 129.2, 128.7, 128.2, 127.7, 126.62, 126.59, 126.3, 125.6, 123.8, 121.1, 120.8, 70.7, 70.5, 35.1 ppm. HRMS (ESI): *m*/*z* calcd. for C_28_H_20_N_3_OS_2_ [M + H]^+^ 478.1043, found 478.1006.
*(2′R,5S)-1-(Benzo[d]thiazol-2-yl)-5-(4-nitrophenyl)-2-thioxospiro[imidazolidine-4,2′-inden]-1′(3′H)-one* (**3ma**). Initially, **1m** (28.3 mg, 0.10 mmol) and **2a** (22.7 mg, 0.12 mmol) were purified by silica gel (200–300 mesh) column chromatography using petroleum ether/ethyl acetate (2/1) as eluent to obtain 34.5 mg (90% yield) of compound **3ma** as a white solid, m.p. 182–184 °C. HPLC (Daicel Chiralpak IC, *n*-hexane/isopropanol = 80:20, flow rate 1.0 mL/min, detection at 254 nm): major diastereomer: *t*_R_ = 12.0 min (major enantiomer), *t*_R_ = 16.9 min (minor enantiomer); 97% *ee*. ${\left[\alpha \right]}_{D}^{25}$ = −633.4 (*c* = 0.43, CH_2_Cl_2_). ^1^H NMR (400 MHz, DMSO-*d*_6_): δ 10.45 (s, 1H, NH), 8.29 (d, *J* = 9.2 Hz, 2H, ArH), 7.95 (d, *J* = 7.2 Hz, 1H, ArH), 7.80 (d, *J* = 7.6 Hz, 1H, ArH), 7.76–7.72 (m, 1H, ArH), 7.68–7.61 (m, 2H, ArH), 7.54–7.48 (m, 3H, ArH), 7.36–7.32 (m, 1H, ArH), 7.29–7.25 (m, 1H, ArH), 6.42 (s, 1H, CH), 3.01 (d, *J* = 17.8 Hz, 1H, CH_2_), 2.81 (d, *J* = 17.8 Hz, 1H, CH_2_) ppm. ^13^C NMR (100 MHz, DMSO-*d*_6_ + CD_3_OD): δ 201.5, 178.3, 157.5, 151.2, 147.8, 147.7, 144.5, 136.4, 132.9, 132.0, 128.5, 126.8, 126.4, 124.8, 124.2, 124.0, 121.6, 120.6, 69.5, 67.9, 34.5 ppm. HRMS (ESI): *m*/*z* calcd. for C_24_H_17_N_4_O_3_S_2_ [M + H]^+^ 473.0737, found 473.0707.
*(2′R,5S)-1-(Benzo[d]thiazol-2-yl)-5-(2,5-dichlorophenyl)-2-thioxospiro[imidazolidine-4,2′-inden]-1′(3′H)-one* (**3na**). Initially, **1n** (30.6 mg, 0.10 mmol) and **2a** (22.7 mg, 0.12 mmol) were purified by silica gel (200–300 mesh) column chromatography using petroleum ether/ethyl acetate (2/1) as eluent to obtain 30.9 mg (75% yield) of compound **3na** as a white solid, m.p. 268–270 °C. HPLC (Daicel Chiralpak ADH, *n*-hexane/isopropanol = 80:20, flow rate 1.0 mL/min, detection at 254 nm): major diastereomer: *t*_R_ = 23.99min (major enantiomer), *t*_R_ = 38.01 min (minor enantiomer); 87% *ee*. ${\left[\alpha \right]}_{D}^{25}$ = −275.1 (*c* = 0.64, CH_2_Cl_2_). ^1^H NMR (400 MHz, CDCl_3_) δ 7.80 (d, *J* = 7.6 Hz, 1H, ArH), 7.73 (d, *J* = 8.0 Hz, 1H, ArH), 7.63 (t, *J* = 7.2 Hz, 1H, ArH), 7.57 (d, *J* = 8.0 Hz, 1H, ArH), 7.44–7.41 (m, 2H, ArH), 7.35–7.28 (m, 3H, ArH + NH), 7.25–7.18 (m, 2H, ArH), 7.11 (d, *J* = 8.4 Hz, 1H, ArH), 6.58 (s, 1H, CH), 3.02 (d, *J* = 18.0 Hz, 1H, CH_2_), 2.91 (d, *J* = 18.0 Hz, 1H, CH_2_) ppm. ^13^C NMR (176 MHz, CDCl_3_): δ 200.4, 179.2, 156.8, 150.3, 148.2, 136.6, 135.1, 134.5, 132.72, 132.68, 132.2, 129.6, 128.8, 128.3, 127.8, 126.3, 125.8, 125.6, 124.0, 121.5, 120.9, 70.0, 66.2, 35.0 ppm. HRMS (ESI): *m*/*z* calcd. for C_24_H_16_Cl_2_N_3_OS_2_ [M + H]^+^ 496.0107, found 496.0091.

### 4.4. Procedure for the Scaled-Up Synthesis of Compound **3aa**

Initially, 2-benzothiazolimine **1a** (714 mg, 3.0 mmol), 2-isothiocyanate-1-indanone **2a** (680.4 mg, 3.6 mmol), and the chiral organocatalyst **C7** (159.5 mg, 0.3 mmol, 0.1 equiv) were added to a 100 mL dry round-bottom flask and dissolved in 40 mL of dichloromethane. After stirring at −18 °C for 14 h, the reaction mixture was concentrated and directly purified by silica gel column chromatography (petroleum ether/ethyl acetate = 2:1) to afford the desired product **3aa** as a white solid (1.06 g, 83% yield) with >20:1 dr and 95% ee.

## Figures and Tables

**Table 1:** Optimization of the reaction conditions ^a^.

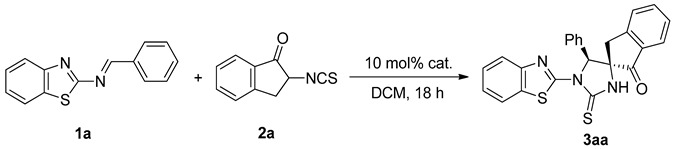					
Entry	Solvent	Catalyst	Yield ^b^ (%)	dr ^c^	ee ^d^ (%)
1	CH_2_Cl_2_	**C1**	75	>20:1	83
2	CH_2_Cl_2_	**C2**	78	>20:1	92
3	CH_2_Cl_2_	**C3**	84	>20:1	82
4	CH_2_Cl_2_	**C4**	80	>20:1	79
5	CH_2_Cl_2_	**C5**	76	>20:1	52
6	CH_2_Cl_2_	**C6**	71	>20:1	7
7	CH_2_Cl_2_	**C7**	83	>20:1	95
8	CH_2_Cl_2_	**C8**	80	>20:1	89
9	CH_2_Cl_2_	**C9**	81	>20:1	52
10	CH_2_Cl_2_	**C10**	86	>20:1	87
11	CH_2_Cl_2_	**C11**	78	>20:1	73
12	CH_2_Cl_2_	**C12**	77	>20:1	95
13	CH_2_Cl_2_	**C13**	73	>20:1	75
14	CH_2_Cl_2_	**C14**	71	>20:1	4
15	dioxane	**C7**	61	>20:1	74
16	EtOAc	**C7**	76	>20:1	65
17	DCE	**C7**	80	>20:1	71
18	MeCN	**C7**	81	>20:1	43
19	THF	**C7**	83	>20:1	54
20	MTBE	**C7**	77	>20:1	84
21 ^e^	CH_2_Cl_2_	**C7**	79	>20:1	67
22 ^f^	CH_2_Cl_2_	**C7**	83	>20:1	79
23 ^g^	CH_2_Cl_2_	**C7**	85	>20:1	98

^a^ Unless otherwise specified, the reactions were carried out with **1a** (0.10 mmol), **2a** (0.12 mmol), and catalyst (10 mol%) in solvent (1.0 mL) for 18 h. ^b^ Isolated yields after column chromatography purification. ^c^ Determined by ^1^H NMR analysis. ^d^ The enantiomeric excess (ee) was determined by HPLC analysis. ^e^ 5 mol% catalyst was used. ^f^ 15 mol% catalyst was used. ^g^ Reaction at −18 °C for 18 h.

## Data Availability

Data are contained within the article and Supplementary Materials.

## Supplementary Materials

The following supporting information can be downloaded at: https://www.mdpi.com/article/10.3390/molecules29132958/s1, Spectroscopic data (^1^H and ^13^C NMR), X-ray single-crystal data and chiral HPLC chromatograms for all new compounds **3**.
