# Supplementary material for: Asymmetric Mannich/Cyclization Reaction of 2-Benzothiazolimines and 2-Isothiocyano-1-indanones to Construct Chiral Spirocyclic Compounds
Source: Molecules. 2024 Jun 21;29(13):2958. doi: 10.3390/molecules29132958 (PMC11242980; doi:10.3390/molecules29132958)
Supplement: Supplementary file 1 [file molecules-29-02958-s001.zip › molecules-3048853-supplementary.pdf]

Asymmetric Mannich/Cyclization Reaction of 2-Benzothiazolimines and 2-Isothiocyano-1-indanones to Construct Chiral Spirocyclic Compounds

Yao Zheng, Da-Ming Du*

School of Chemistry and Chemical Engineering, Beijing Institute of Technology, Beijing 100081, China

Key Laboratory of Medicinal Molecule Science & Pharmaceutical Engineering, Ministry of Industry and Information Technology, Beijing 100081, China

E-mail: dudm@bit.edu.cn

Supporting Information

Contents

1. Copies of ^{1}H and ^{13}C NMR spectra of new compounds…………….............................S1
2. X-ray single-crystal data for product **3ab**…………………..…..……………………..S43
3. Copies of HPLC chromatograms…………………..…..……………………………..S44

1. **Copies of ^{1}H and ^{13}C NMR spectra of new compounds**

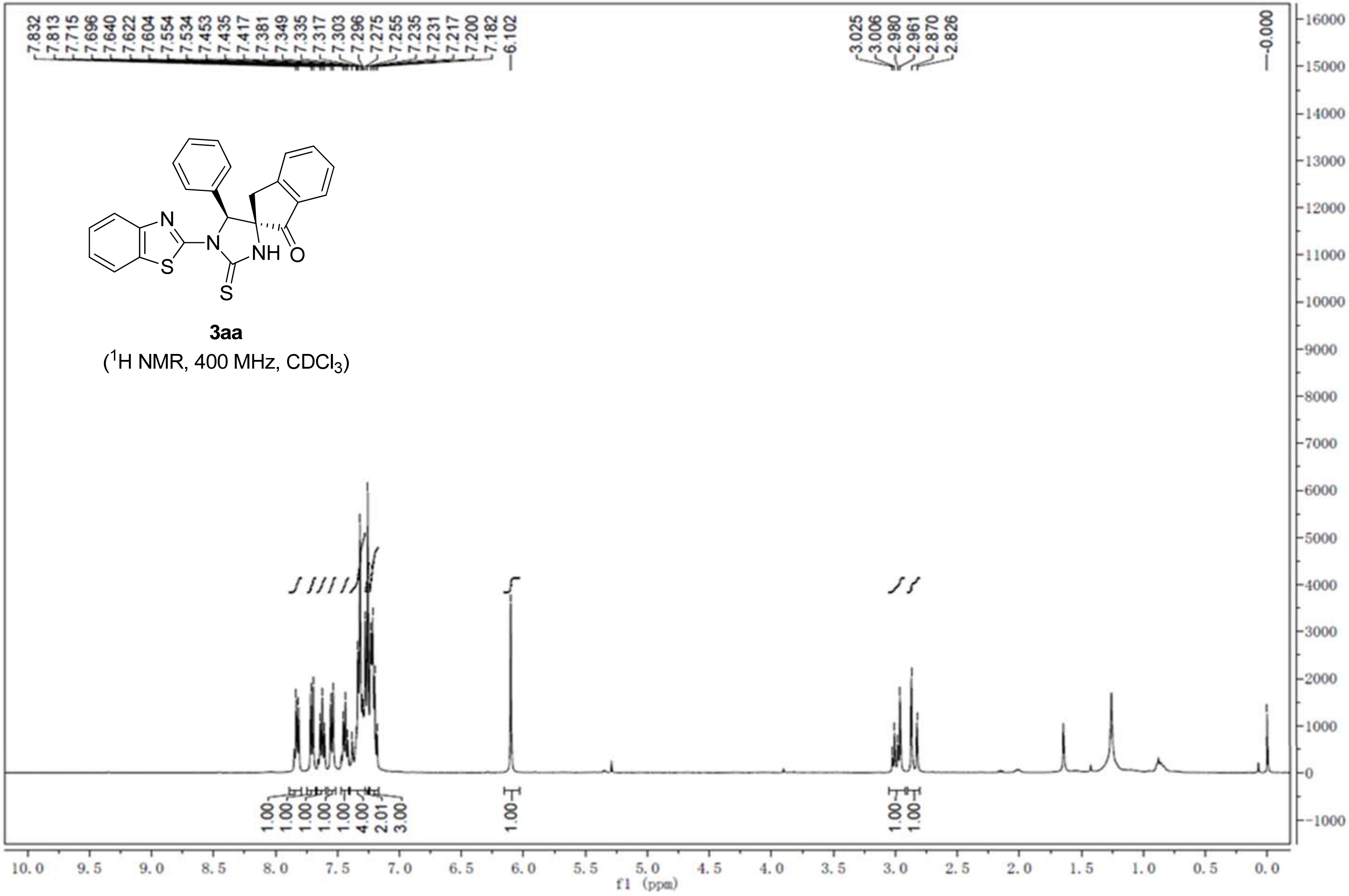

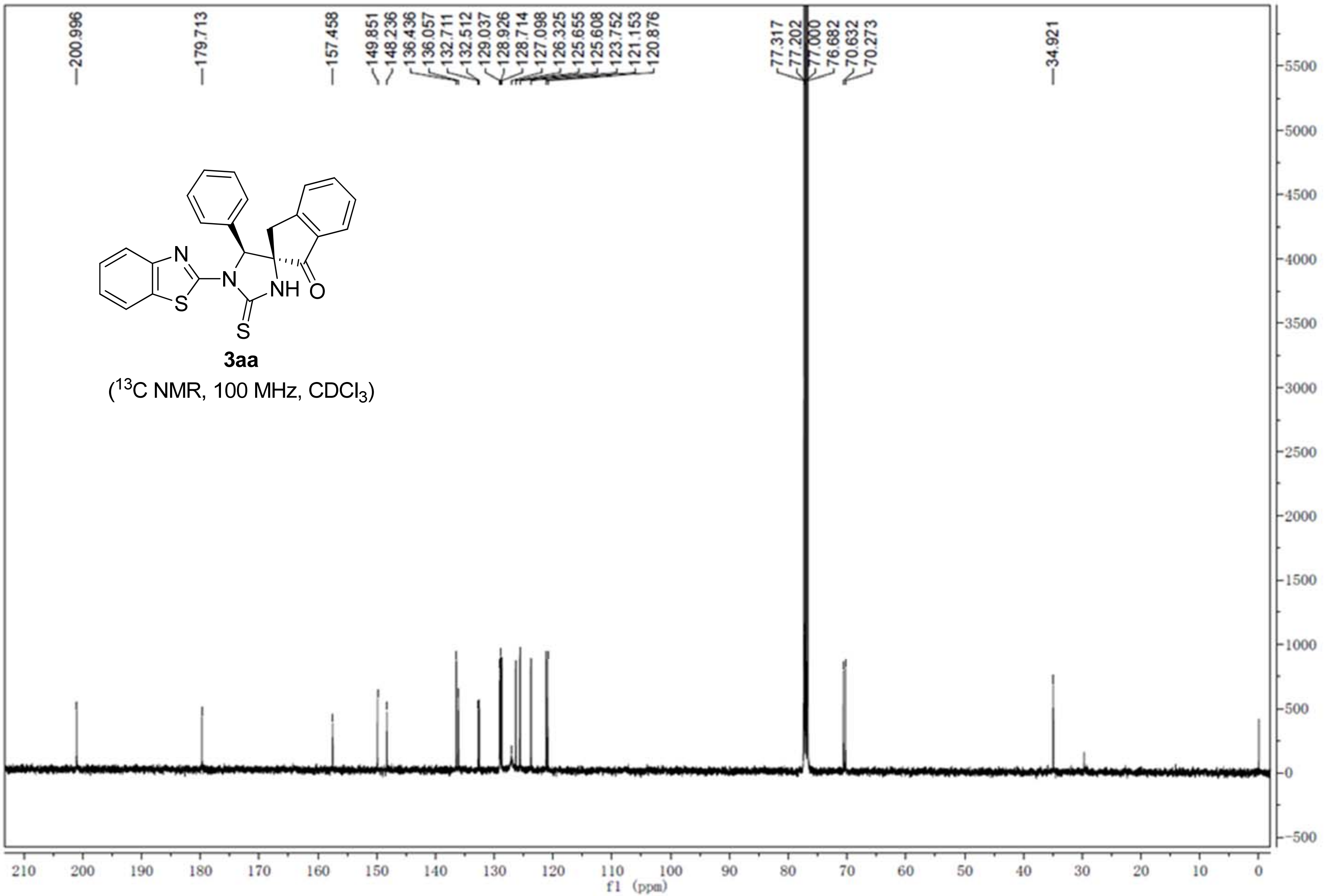
200.996
179.713
157.458
149.851
148.236
136.436
136.057
132.711
132.512
129.037
128.926
128.714
127.098
126.325
125.655
125.608
123.752
121.153
120.876
77.317
77.202
77.000
76.682
70.632
70.273
34.921
N
N
S
NH
O
S
3aa
(^{13}C NMR, 100 MHz, $CDCl_3$)
f1 (ppm)

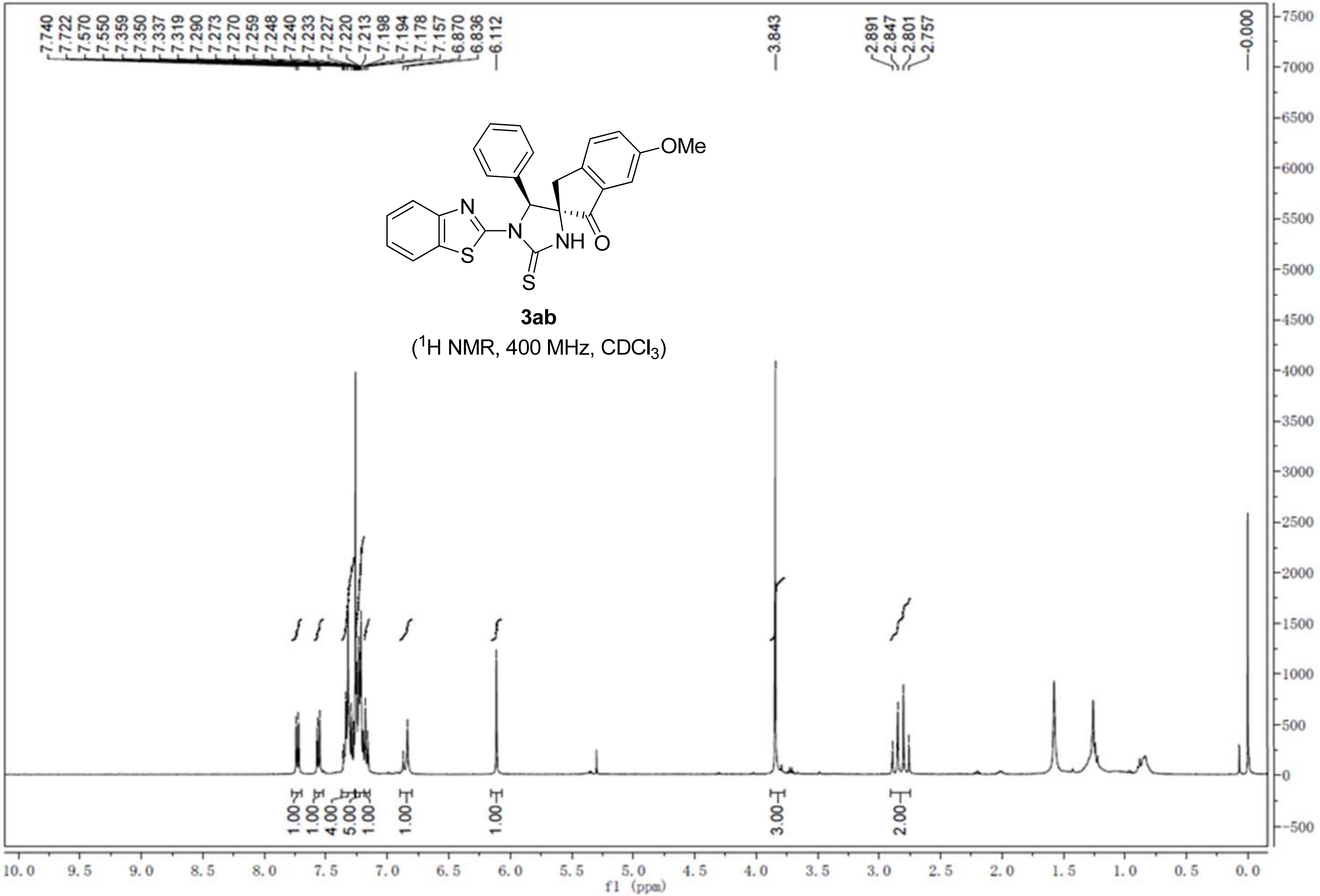
7.740
7.722
7.570
7.550
7.359
7.350
7.337
7.319
7.290
7.273
7.270
7.259
7.248
7.240
7.233
7.227
7.220
7.213
7.198
7.194
7.178
7.157
6.870
6.836
6.112
3.843
2.891
2.847
2.801
2.757
0.000
OMe
N
S
NH
O
S
3ab
(^{1}H NMR, 400 MHz, $CDCl_3$)
1.00
1.00
4.00
5.00
1.00
1.00
1.00
3.00
2.00
f1 (ppm)

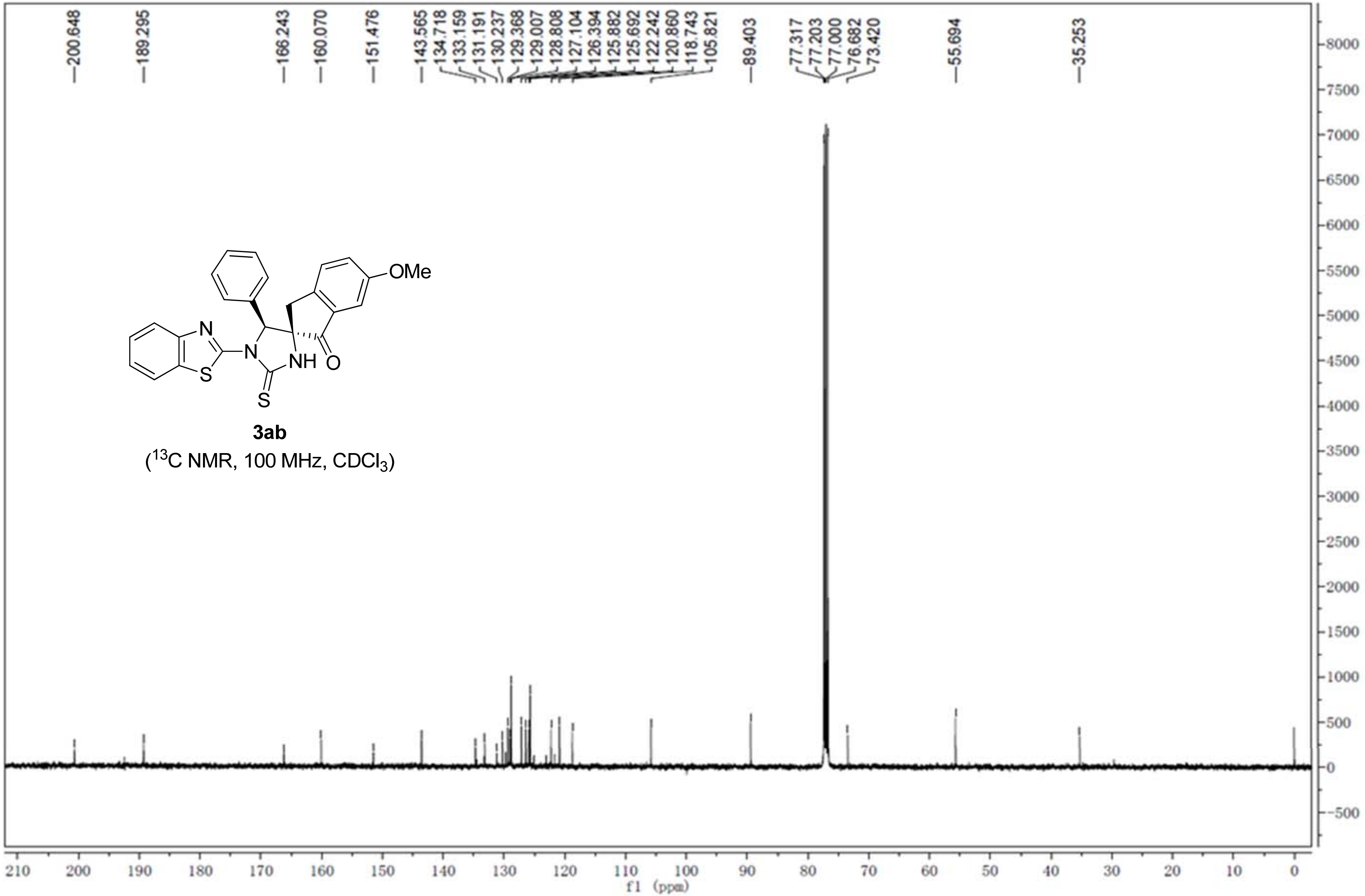

3ab

(^{13}C NMR, 100 MHz, $CDCl_3$)

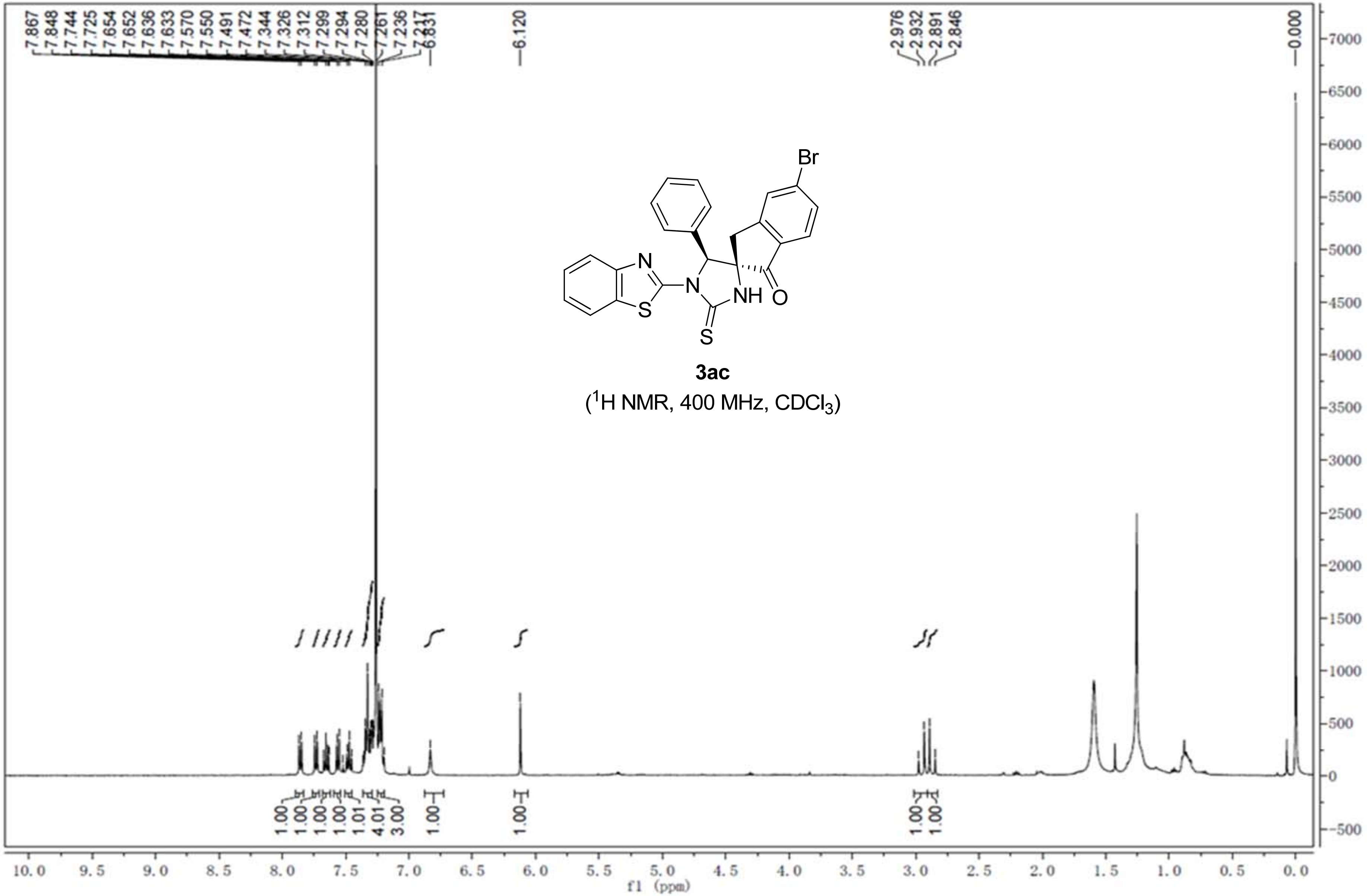
7.867 7.848 7.744 7.725 7.654 7.652 7.636 7.633 7.570 7.550 7.491 7.472 7.344 7.326 7.312 7.299 7.294 7.280 7.261 7.236 7.217 6.831
6.120
2.976 2.932 2.891 2.846
0.000
Br
N
S
N
NH
O
S
3ac
(^{1}H NMR, 400 MHz, CDCl$_3$)
1.00 1.00 1.00 1.00 1.01 4.01 3.00
1.00
1.00
1.00 1.00
f1 (ppm)

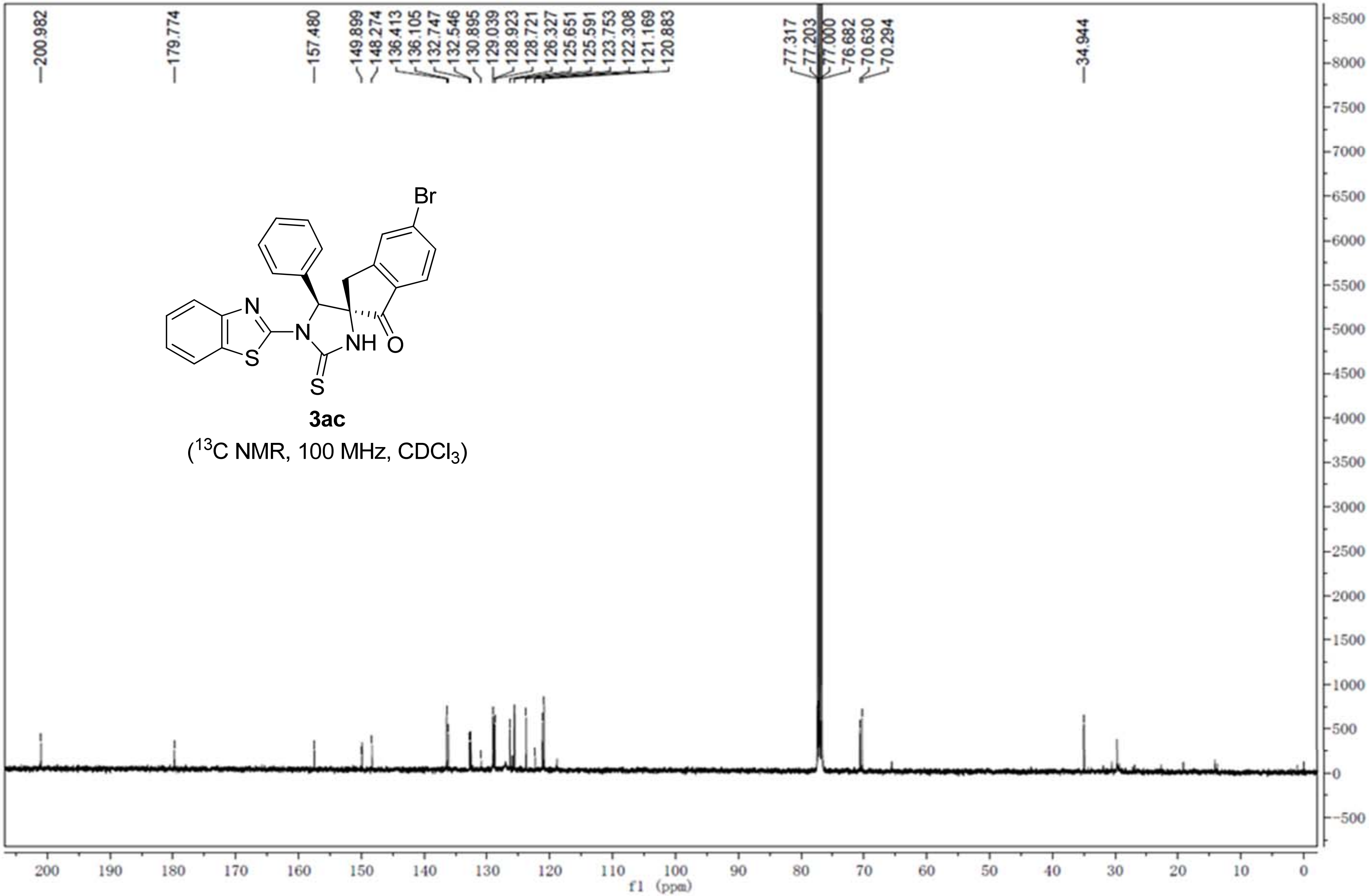
200.982
179.774
157.480
149.899
148.274
136.413
136.105
132.747
132.546
130.895
129.039
128.923
128.721
126.327
125.651
125.591
123.753
122.308
121.169
120.883
77.317
77.203
77.000
76.682
70.630
70.294
34.944
Br
N
S
N
NH
O
S
3ac
(13C NMR, 100 MHz, CDCl3)
f1 (ppm)

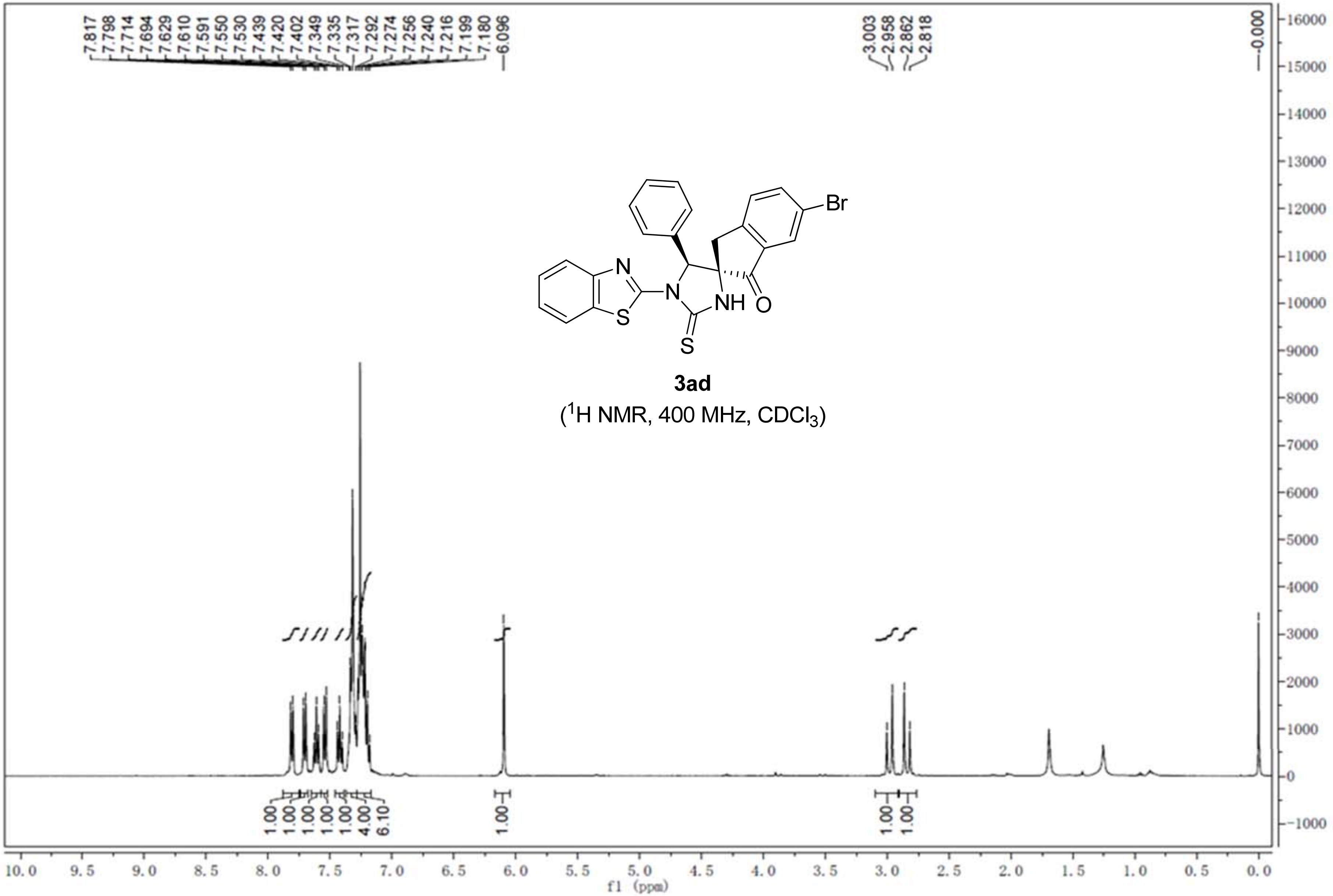
7.817
7.798
7.714
7.694
7.629
7.610
7.591
7.550
7.530
7.439
7.420
7.402
7.349
7.335
7.317
7.292
7.274
7.256
7.240
7.216
7.199
7.180
6.096
3.003
2.958
2.862
2.818
-0.000
N
N
S
NH
O
S
Br
3ad
(^{1}H NMR, 400 MHz, $CDCl_3$)
1.00
1.00
1.00
1.00
1.00
4.00
6.10
1.00
1.00
1.00
f1 (ppm)

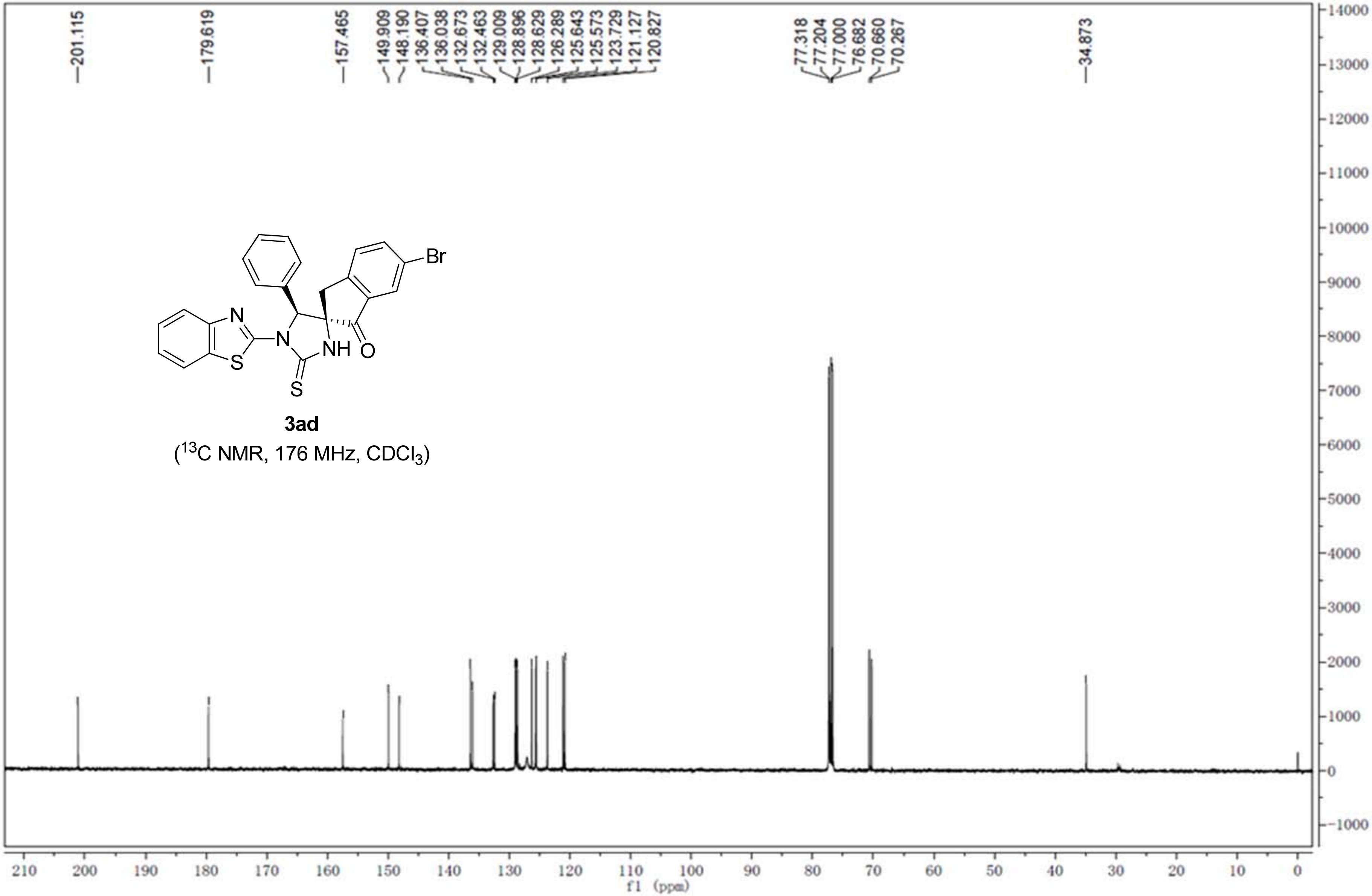

3ad
(^{13}C NMR, 176 MHz, $CDCl_3$)

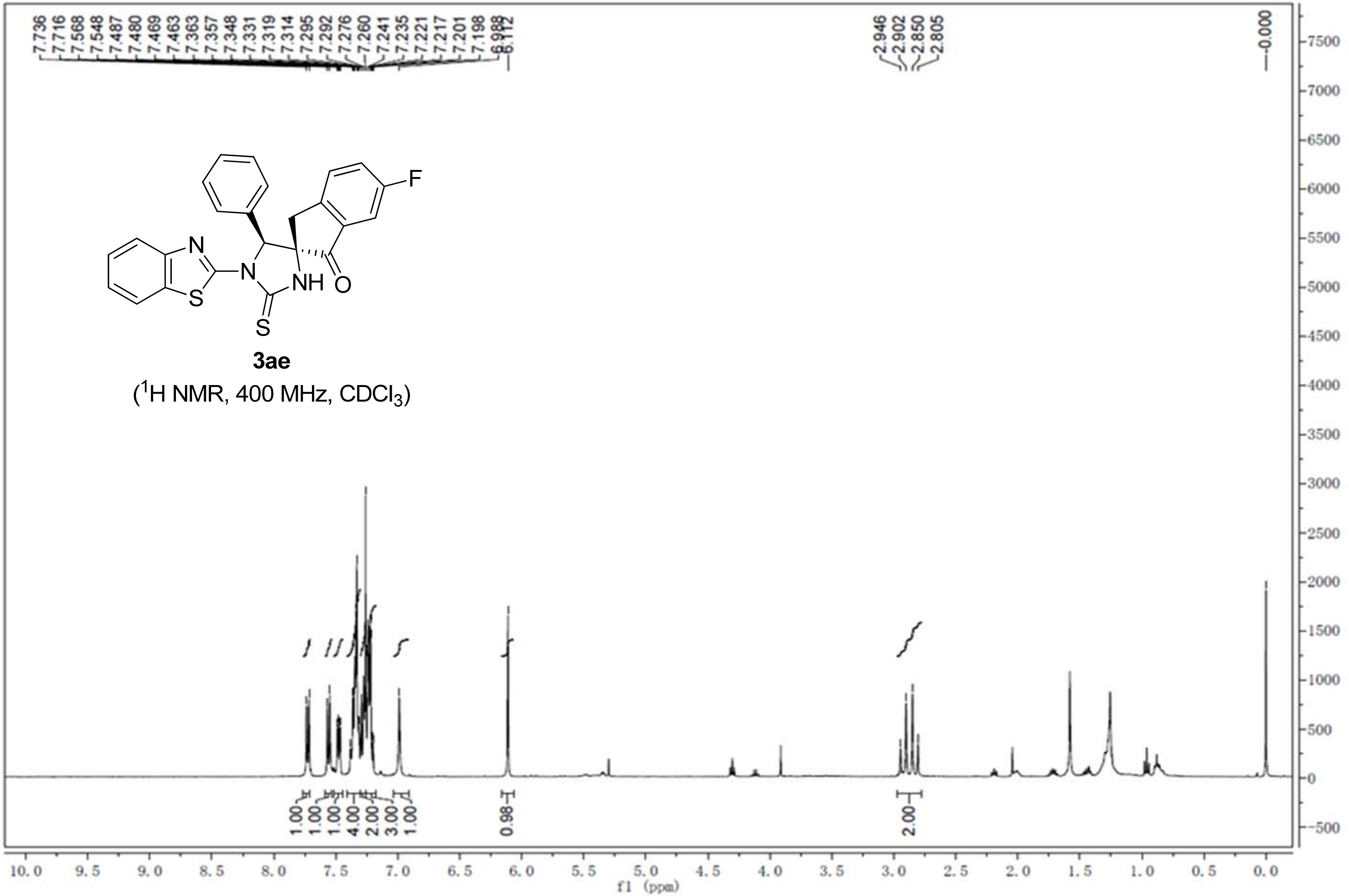

3ae

(^{1}H NMR, 400 MHz, $CDCl_3$)

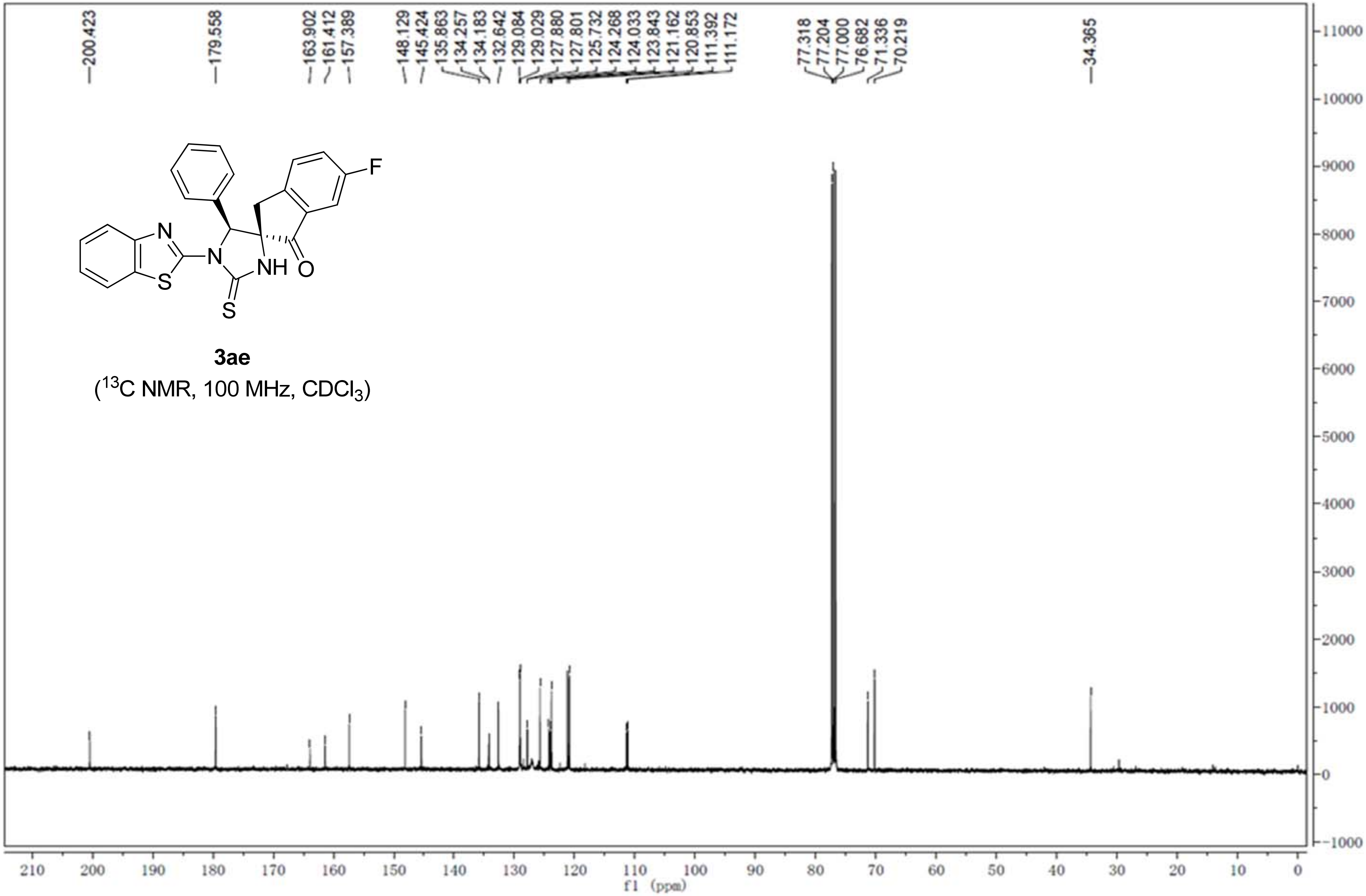

3ae
(^{13}C NMR, 100 MHz, $CDCl_3$)

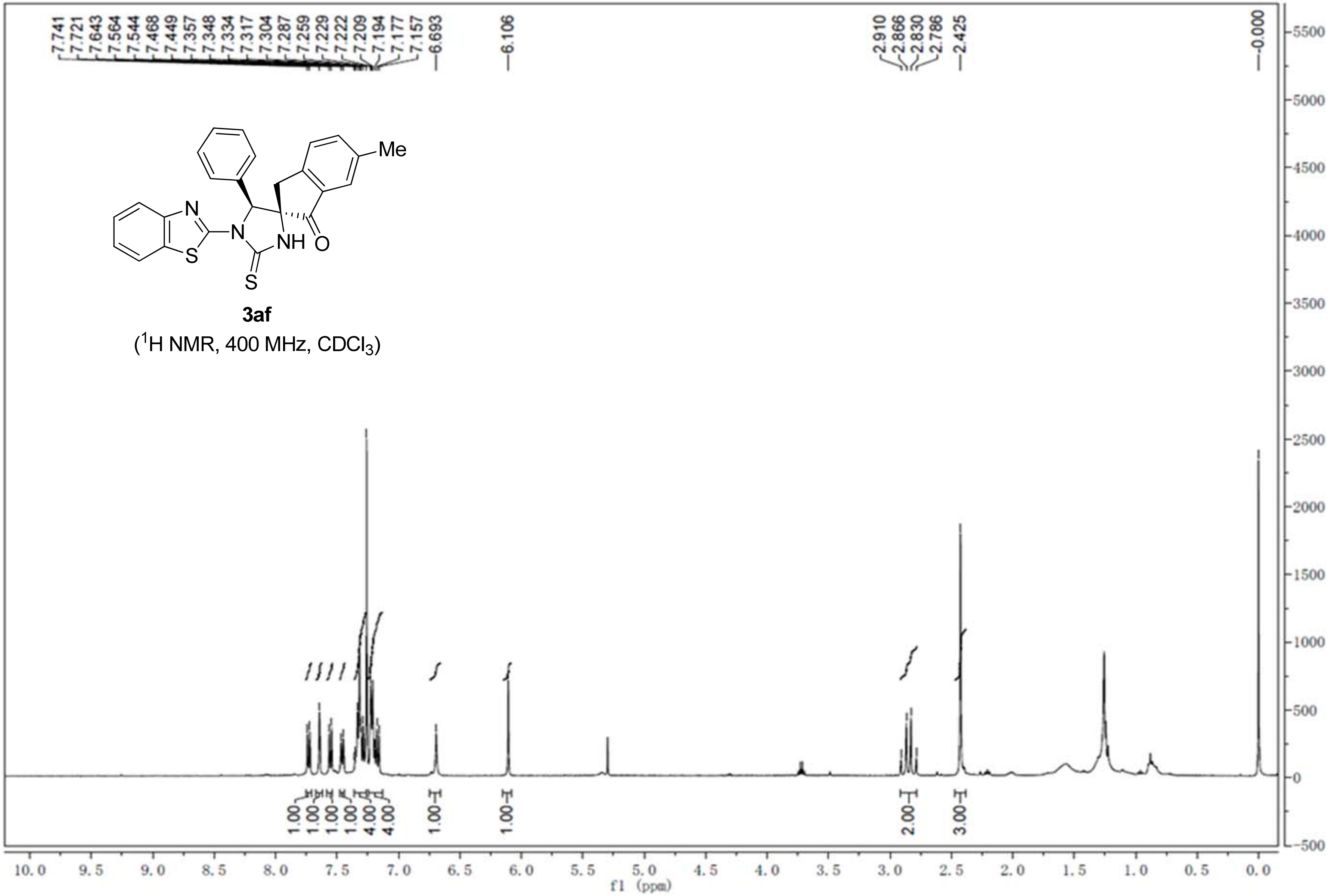

7.741 7.721 7.643 7.564 7.544 7.468 7.449 7.357 7.348 7.334 7.317 7.304 7.287 7.259 7.229 7.222 7.209 7.194 7.177 7.157 6.693
6.106
2.910 2.866 2.830 2.786 2.425
-0.000
Me
N
N
NH
O
S
S
3af
(^{1}H NMR, 400 MHz, $CDCl_3$)
1.00 1.00 1.00 1.00 4.00 4.00
1.00
1.00
2.00
3.00
f1 (ppm)

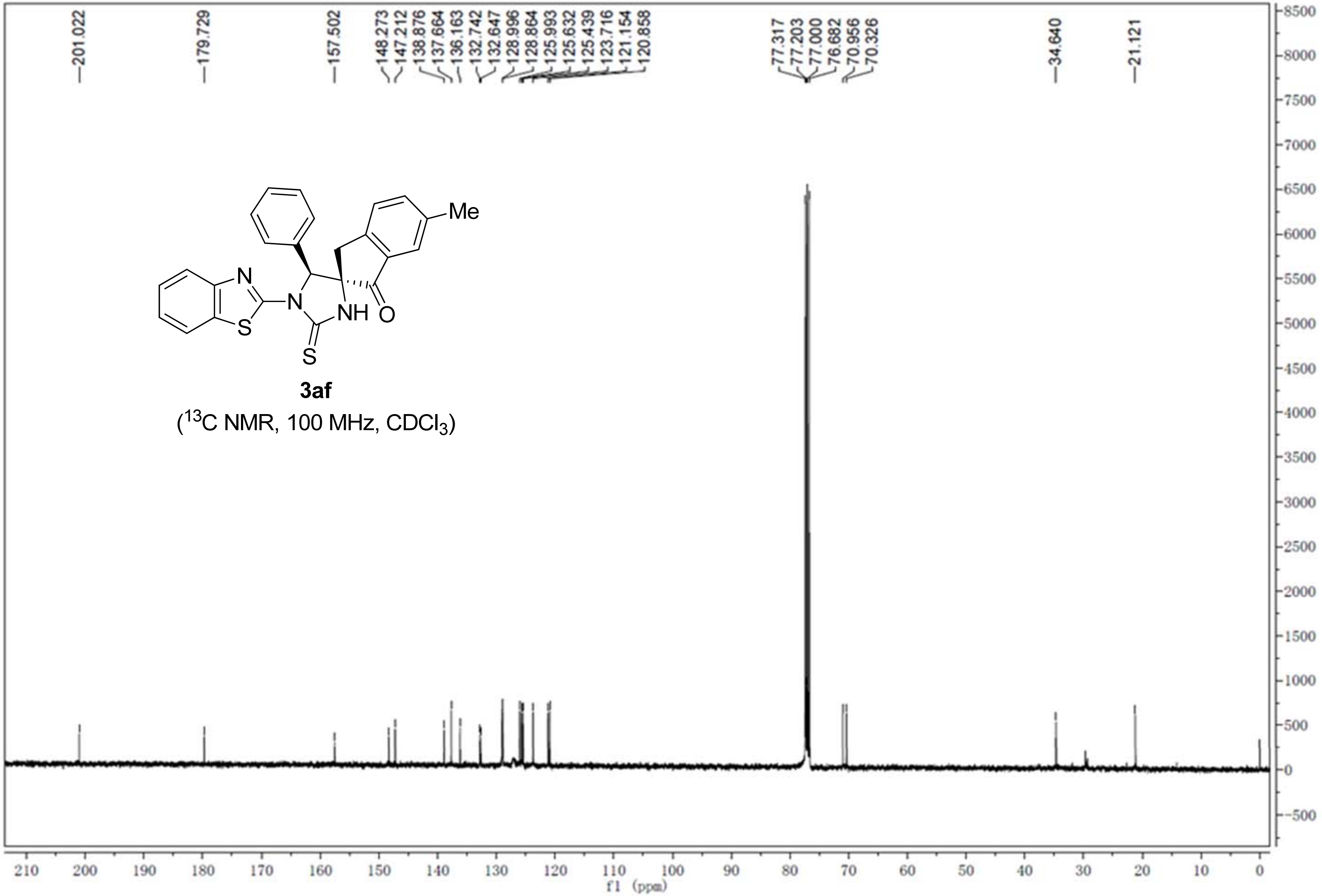

3af

(^{13}C NMR, 100 MHz, $CDCl_3$)

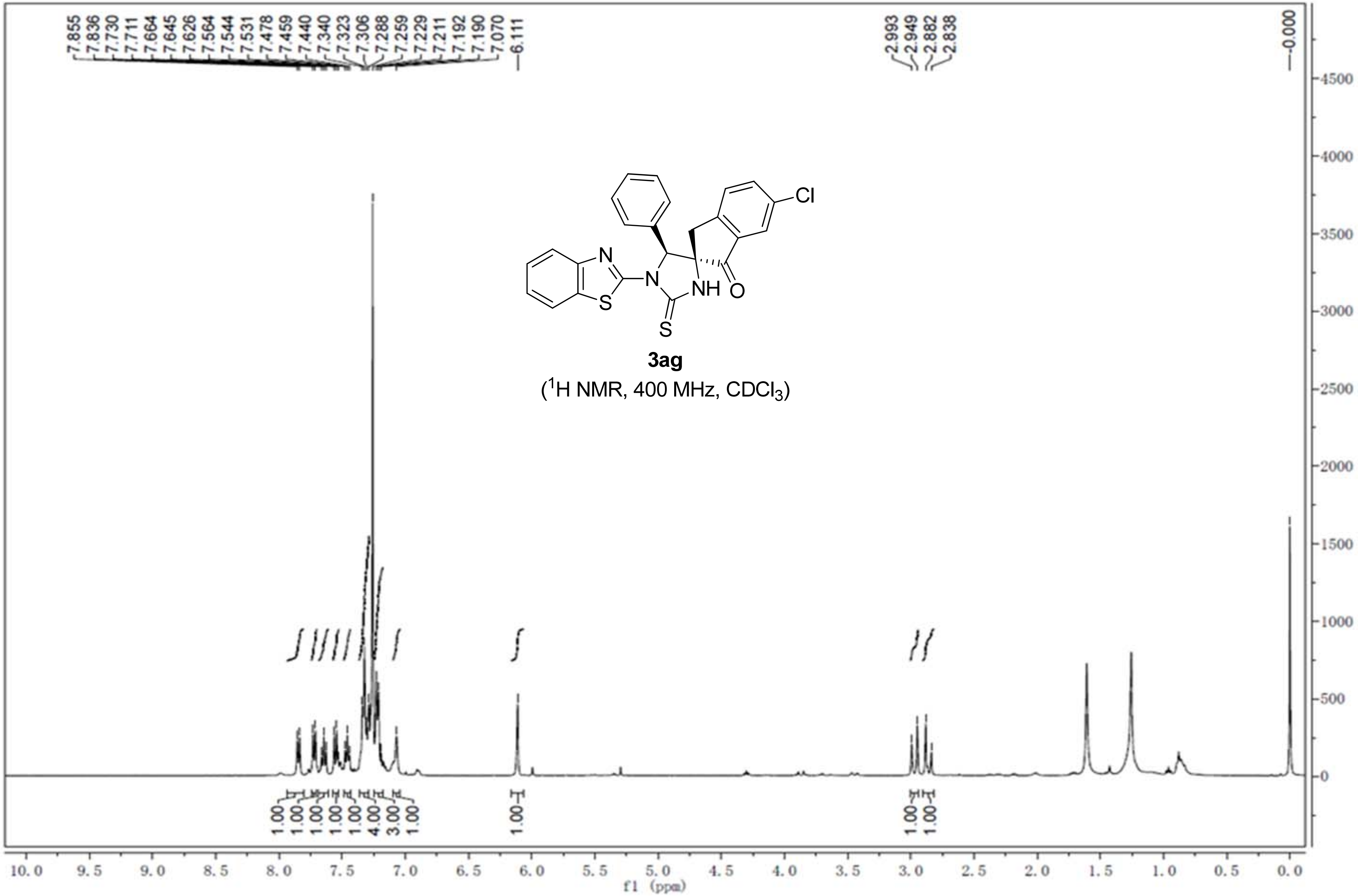
7.855
7.836
7.730
7.711
7.664
7.645
7.626
7.564
7.544
7.531
7.478
7.459
7.440
7.340
7.323
7.306
7.288
7.259
7.229
7.211
7.192
7.190
7.070
6.111
2.993
2.949
2.882
2.838
-0.000
3ag
(^{1}H NMR, 400 MHz, CDCl$_3$)
1.00
1.00
1.00
1.00
1.00
4.00
3.00
1.00
1.00
1.00
1.00
f1 (ppm)

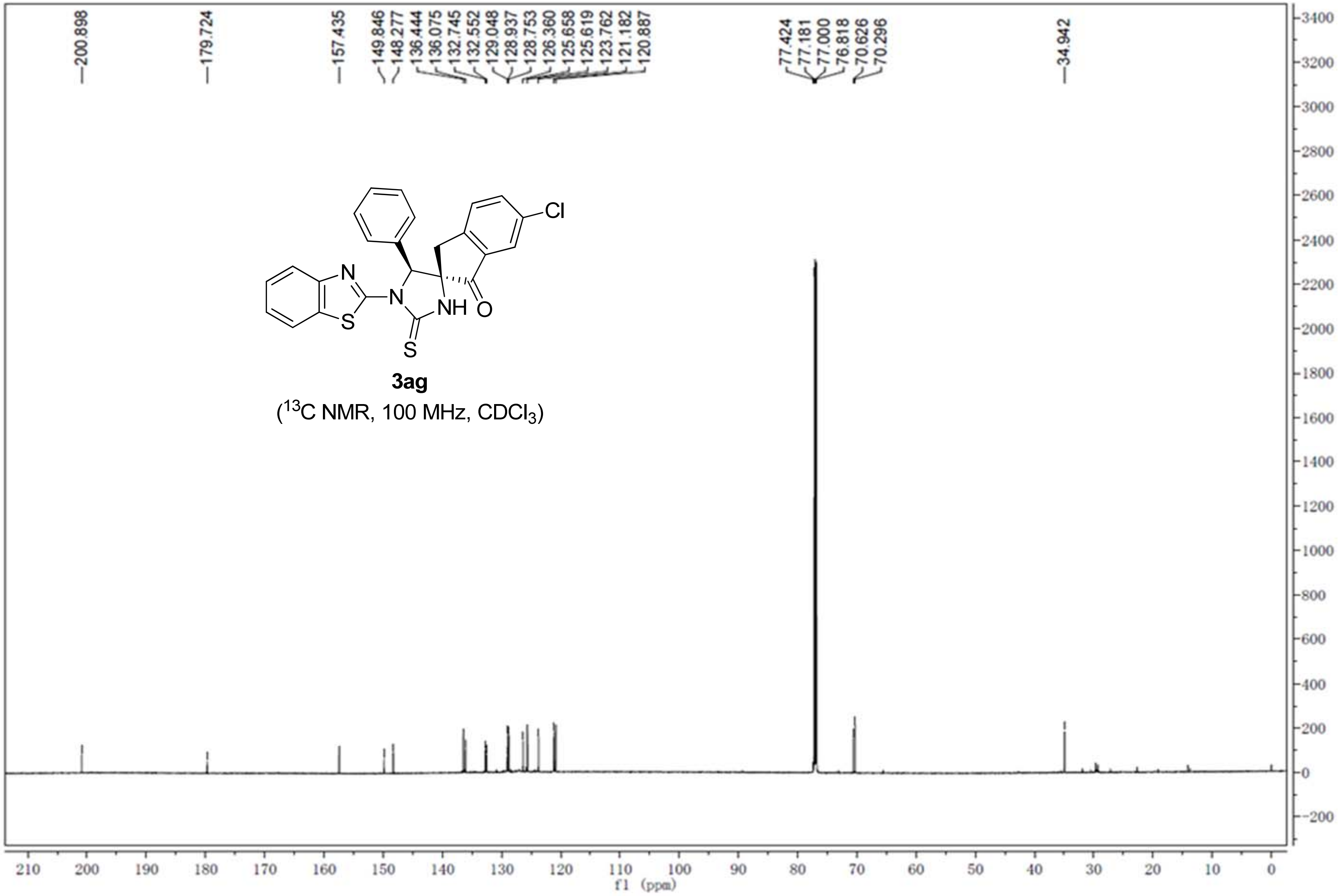

3ag

(^{13}C NMR, 100 MHz, $CDCl_3$)

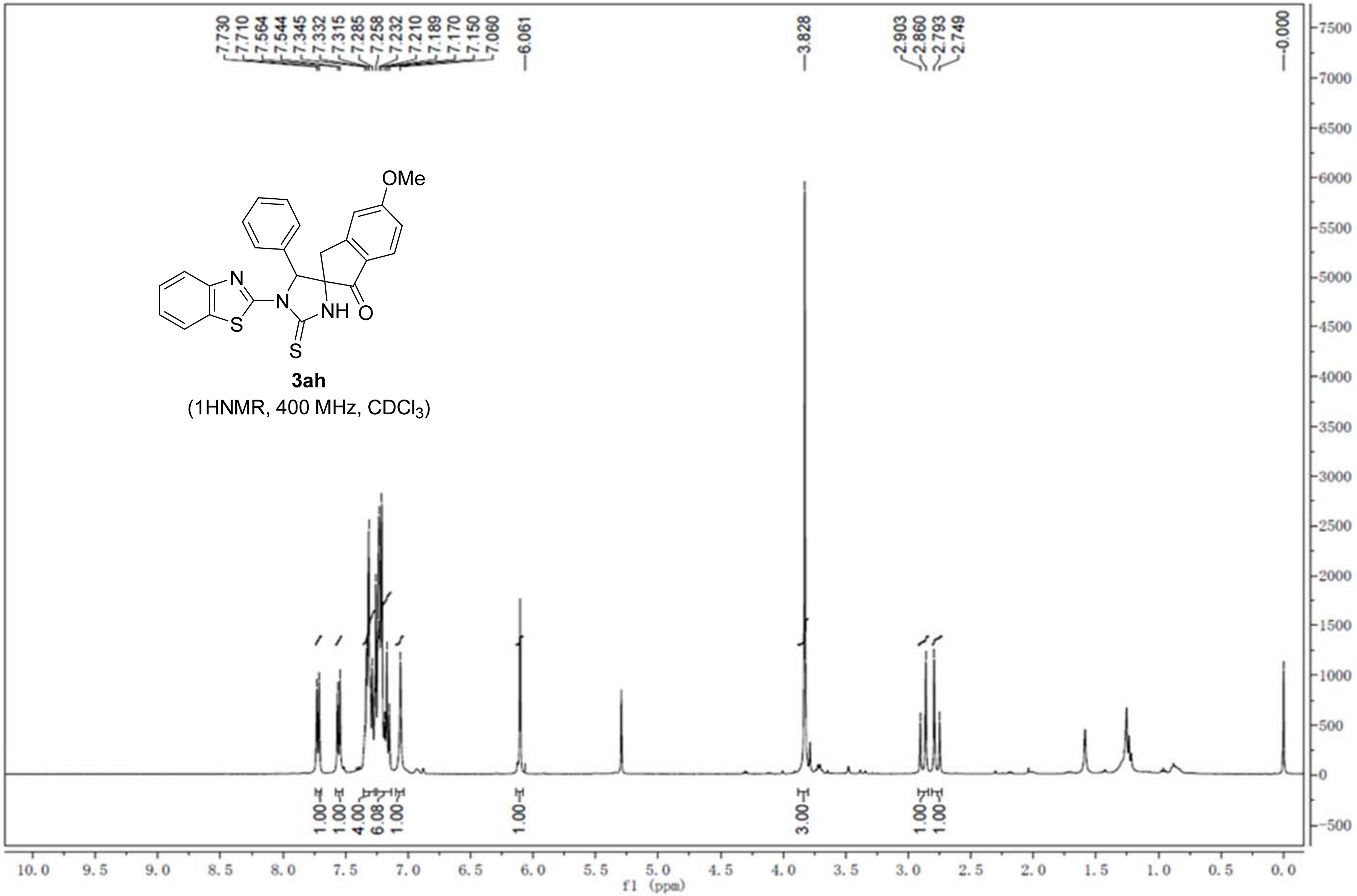
OMe
N
N
NH
O
S
S
3ah
(1HNMR, 400 MHz, $CDCl_3$)
7.730
7.710
7.564
7.544
7.345
7.332
7.315
7.285
7.258
7.232
7.210
7.189
7.170
7.150
7.060
6.061
3.828
2.903
2.860
2.793
2.749
0.000
1.00
1.00
4.00
6.08
1.00
1.00
3.00
1.00
1.00
f1 (ppm)

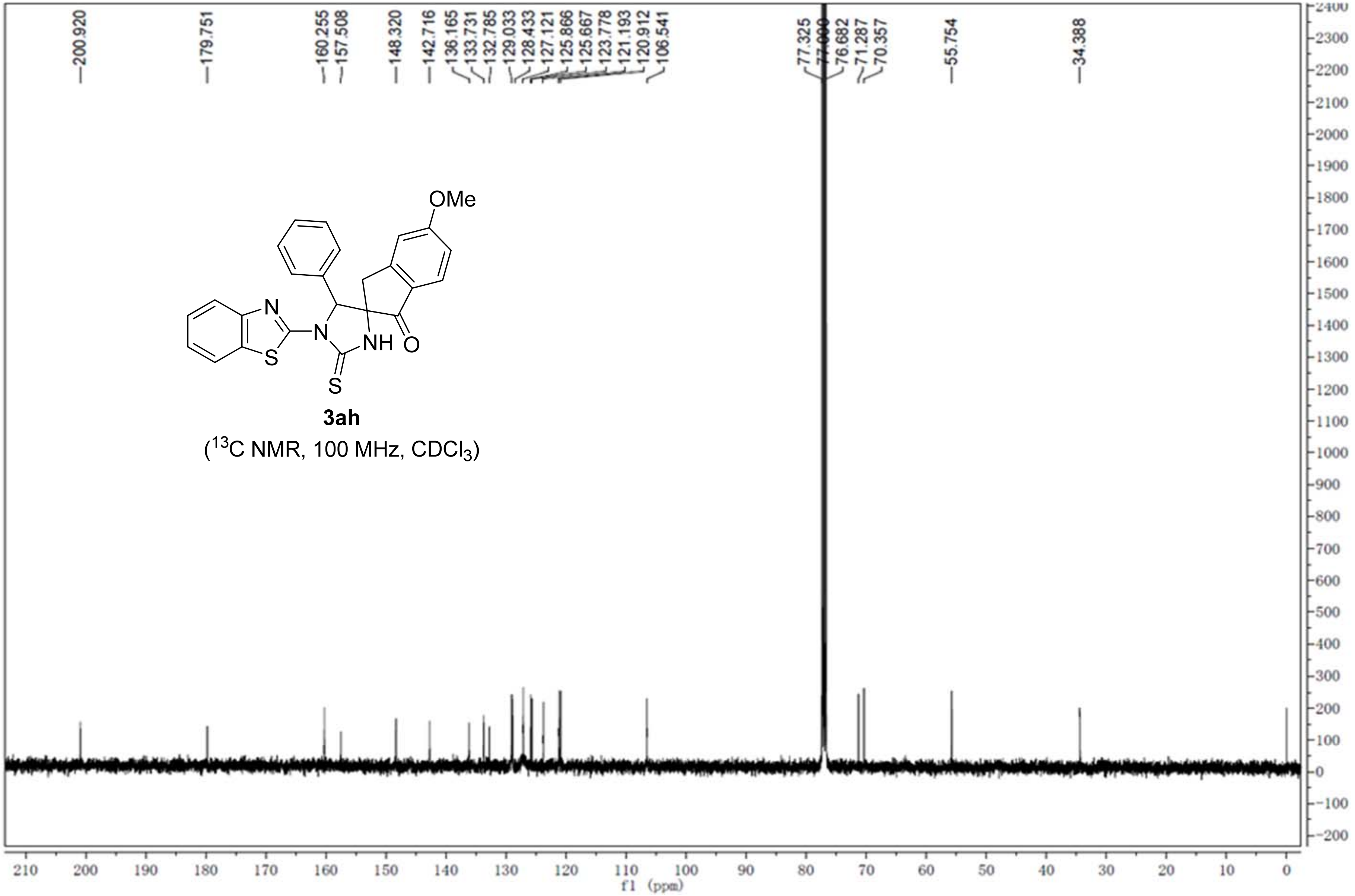

200.920
179.751
160.255
157.508
148.320
142.716
136.165
133.731
132.785
129.033
128.433
127.121
125.866
125.667
123.778
121.193
120.912
106.541
77.325
77.000
76.682
71.287
70.357
55.754
34.388
OMe
N
N
NH
O
S
S
3ah
(^{13}C NMR, 100 MHz, CDCl$_3$)
f1 (ppm)

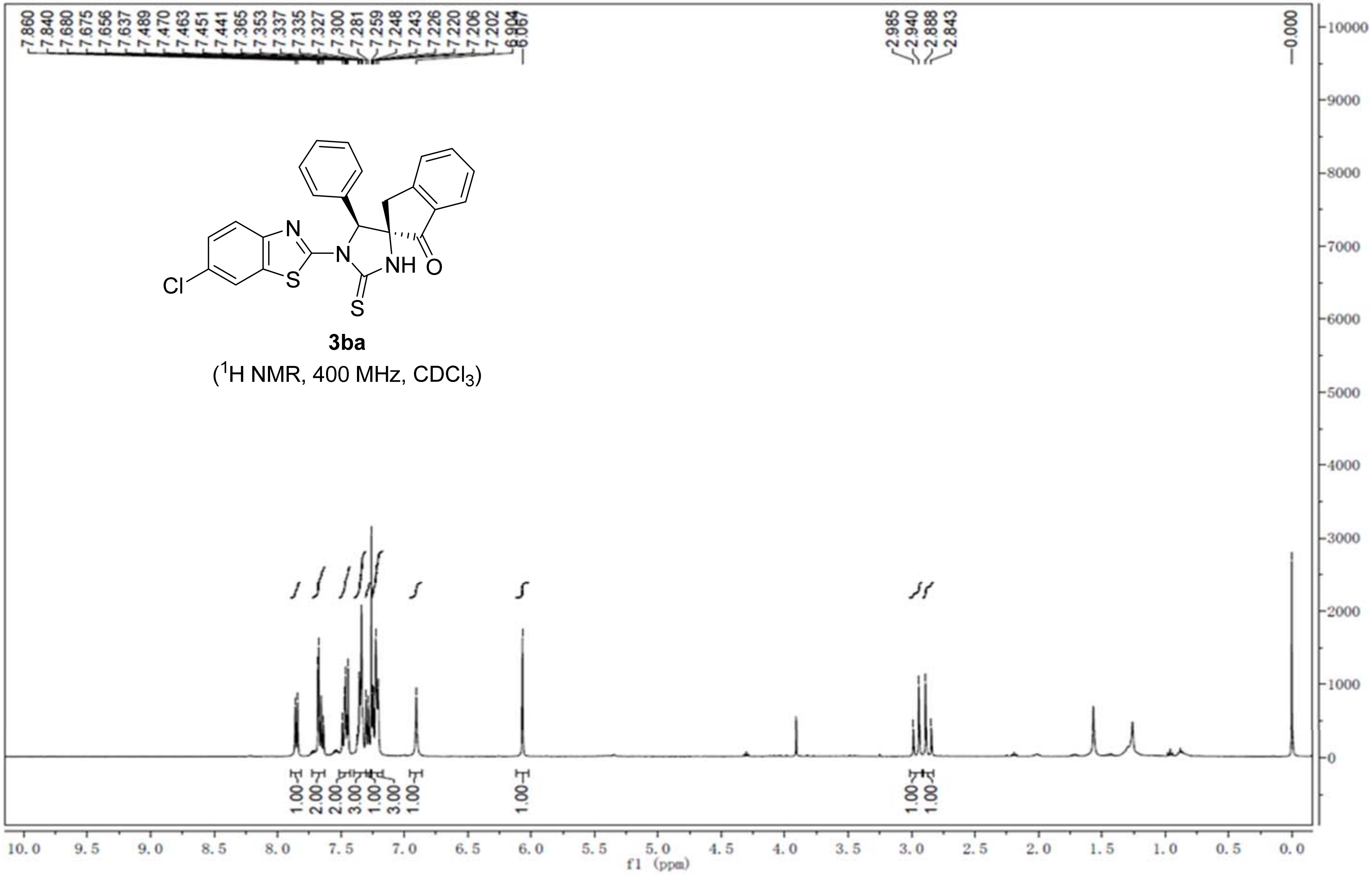

3ba
(¹H NMR, 400 MHz, CDCl₃)
f1 (ppm)

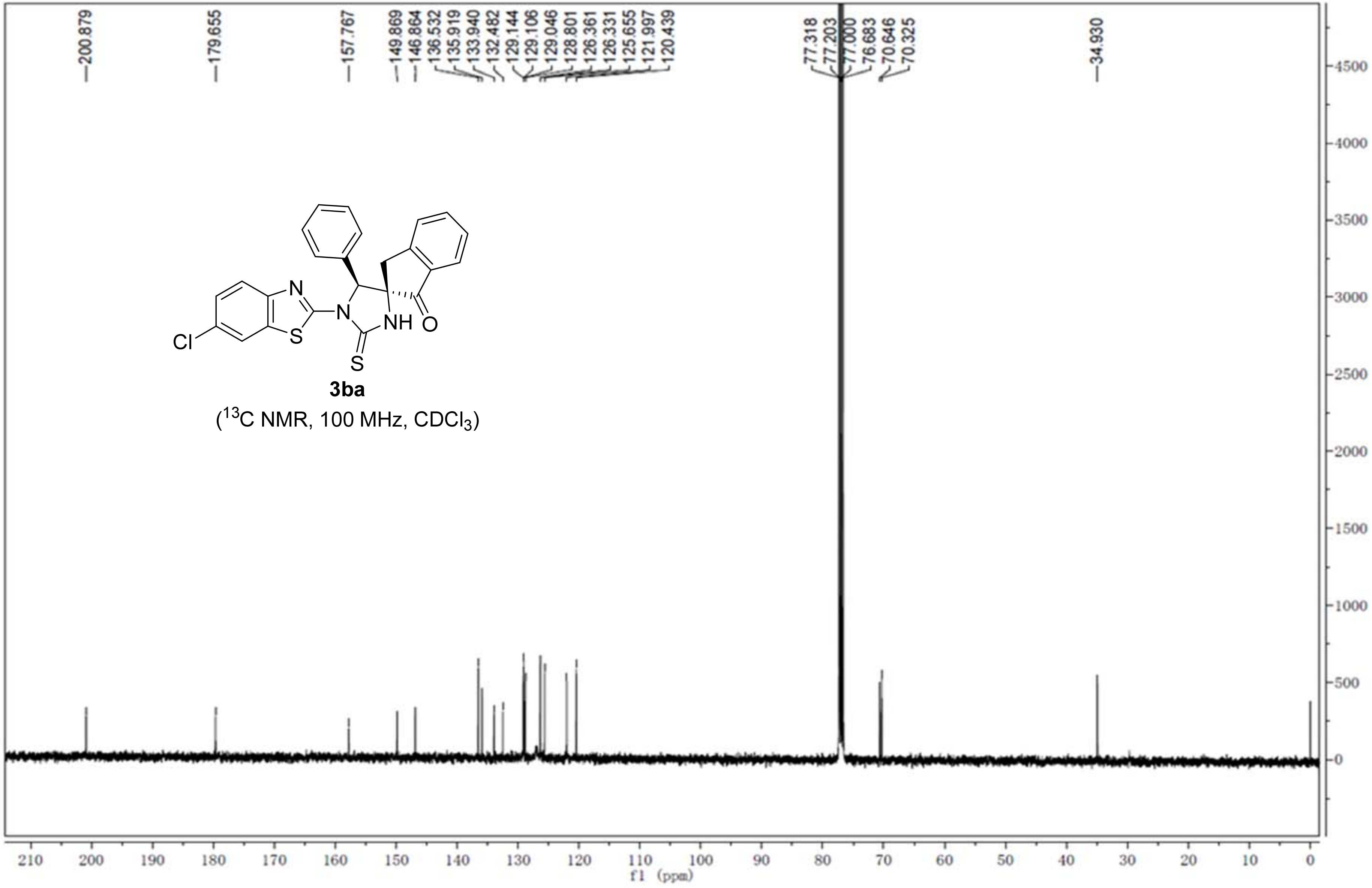
200.879
179.655
157.767
149.869
146.864
136.532
135.919
133.940
132.482
129.144
129.106
129.046
128.801
126.361
126.331
125.655
121.997
120.439
77.318
77.203
77.000
76.683
70.646
70.325
34.930
Cl
N
S
N
NH
O
S
3ba
(^{13}C NMR, 100 MHz, $CDCl_3$)
f1 (ppm)

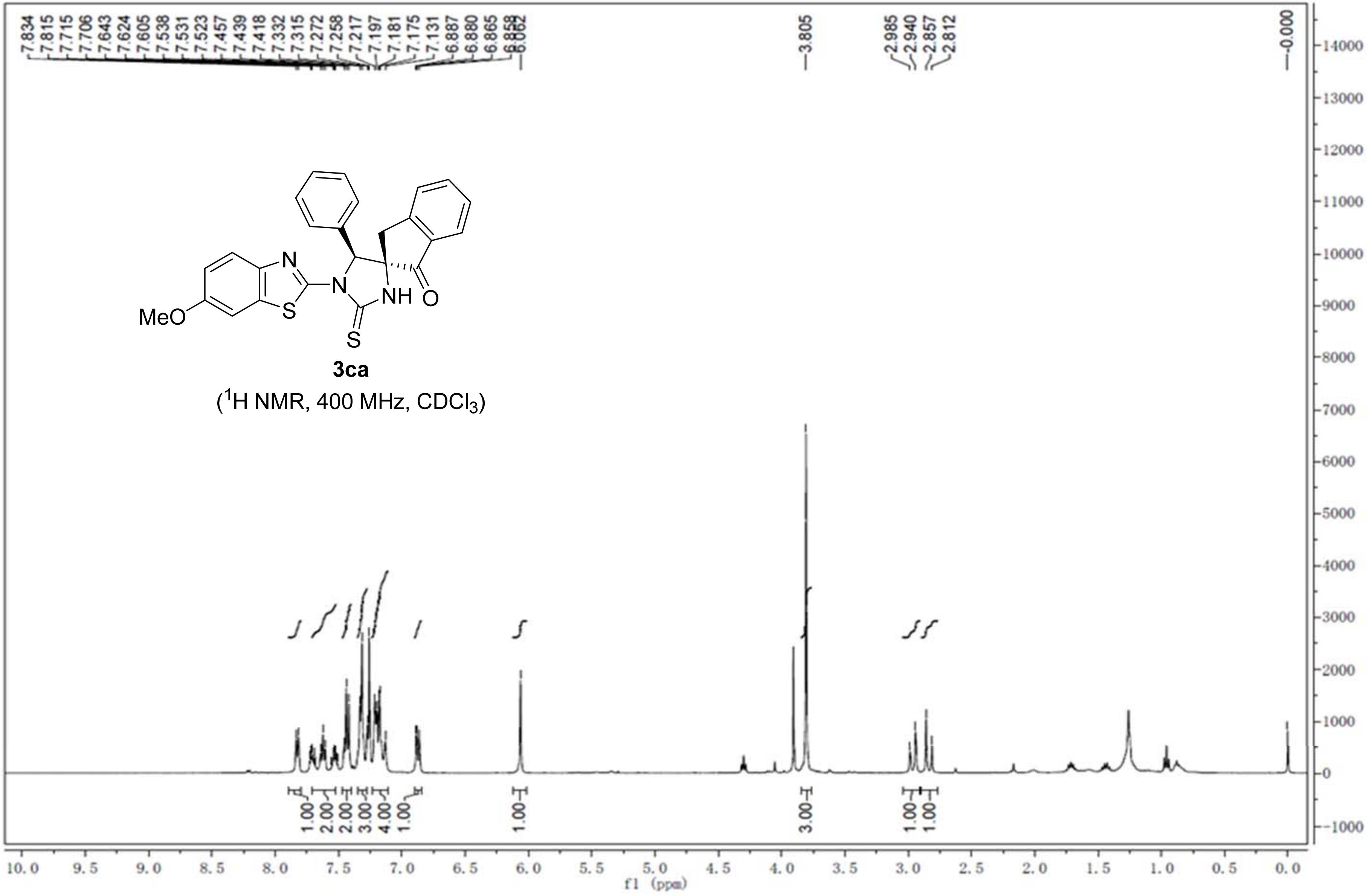
3ca
(1H NMR, 400 MHz, CDCl3)
f1 (ppm)

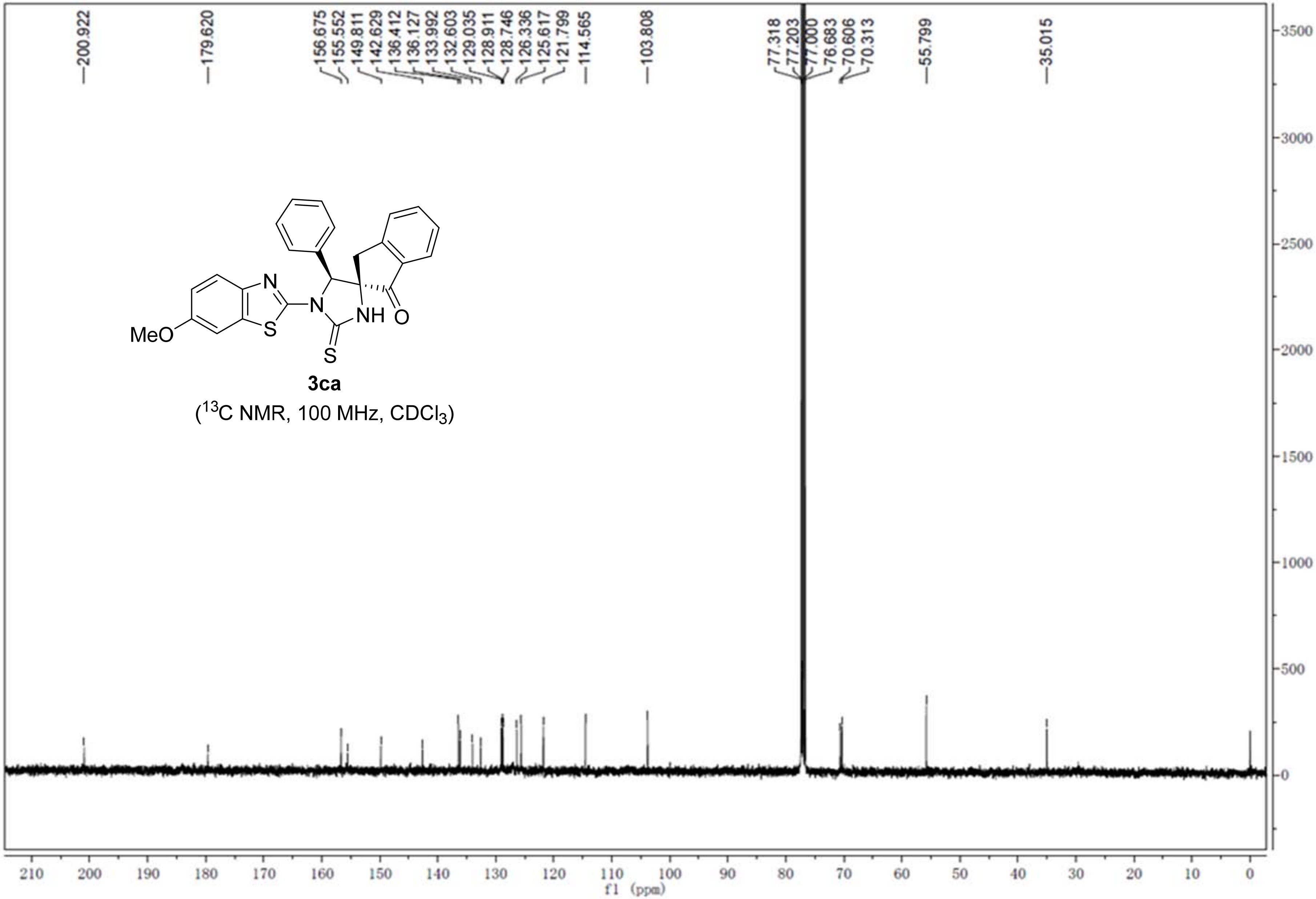
200.922
179.620
156.675
155.552
149.811
142.629
136.412
136.127
133.992
132.603
129.035
128.911
128.746
126.336
125.617
121.799
114.565
103.808
77.318
77.203
77.000
76.683
70.606
70.313
55.799
35.015
MeO
N
S
N
NH
O
S
3ca
(^{13}C NMR, 100 MHz, CDCl$_3$)
f1 (ppm)

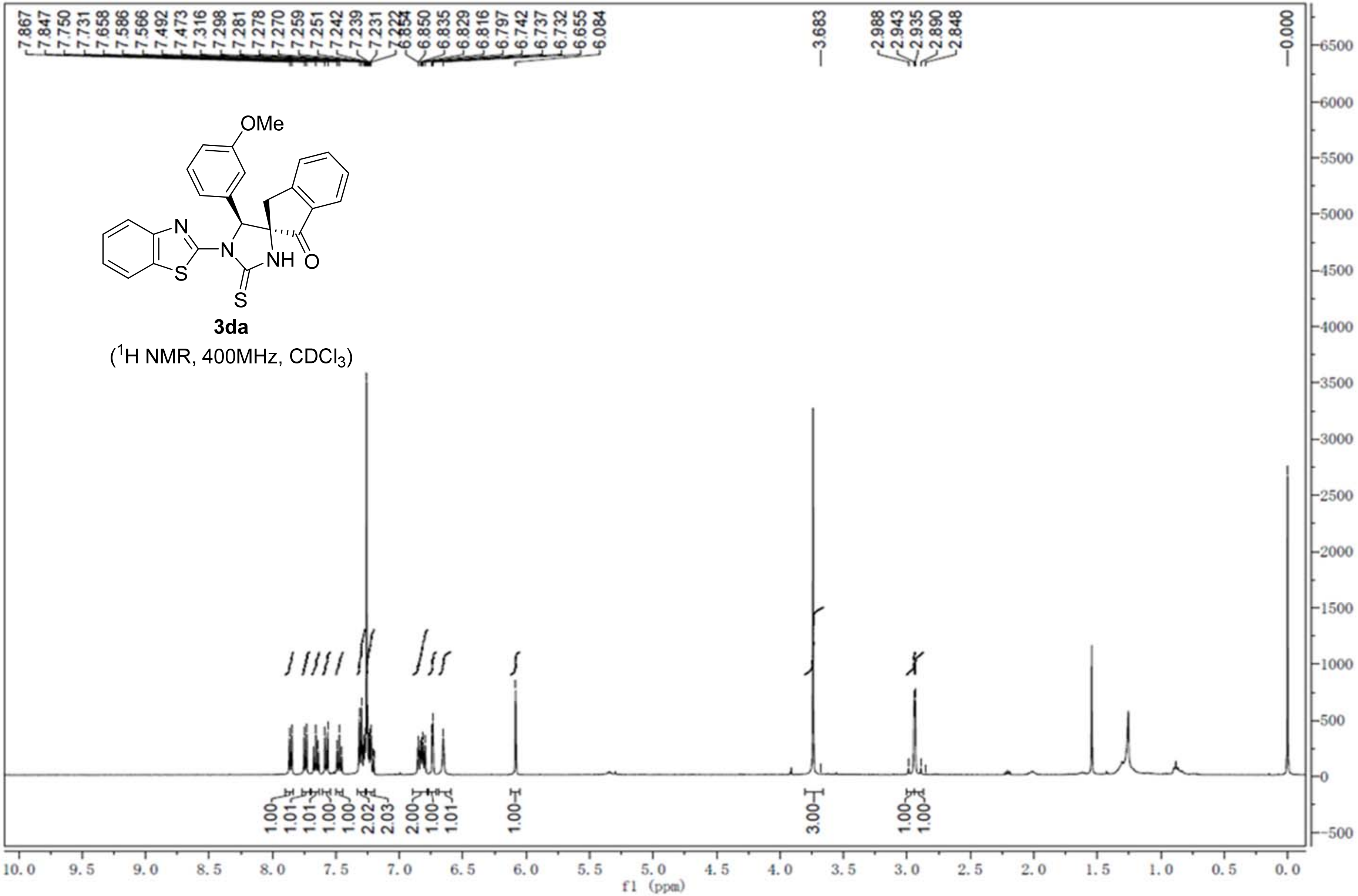
7.867 7.847 7.750 7.731 7.658 7.586 7.566 7.492 7.473 7.316 7.298 7.281 7.278 7.270 7.259 7.251 7.242 7.239 7.231 7.222 6.854 6.850 6.835 6.829 6.816 6.797 6.742 6.737 6.732 6.655 6.084
3.683
2.988 2.943 2.935 2.890 2.848
0.000
OMe
N
S
N
NH
O
S
3da
(^{1}H NMR, 400MHz, $CDCl_3$)
1.00 1.01 1.01 1.00 1.00 2.02 2.03 2.00 1.00 1.01 1.00 3.00 1.00 1.00
f1 (ppm)

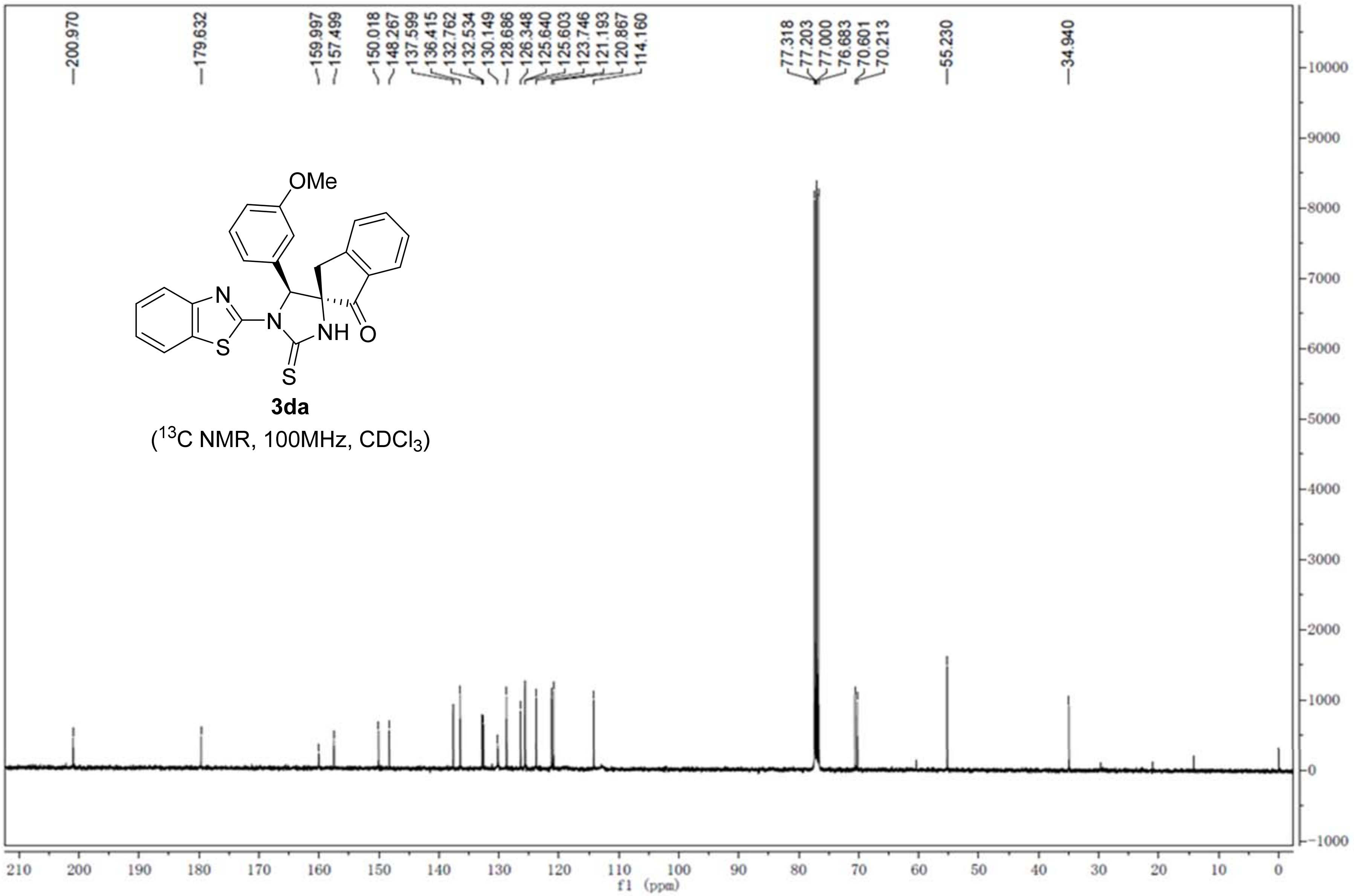
200.970
179.632
159.997
157.499
150.018
148.267
137.599
136.415
132.762
132.534
130.149
128.686
126.348
125.640
125.603
123.746
121.193
120.867
114.160
77.318
77.203
77.000
76.683
70.601
70.213
55.230
34.940
OMe
N
N
S
NH
O
S
3da
(13C NMR, 100MHz, CDCl3)
f1 (ppm)

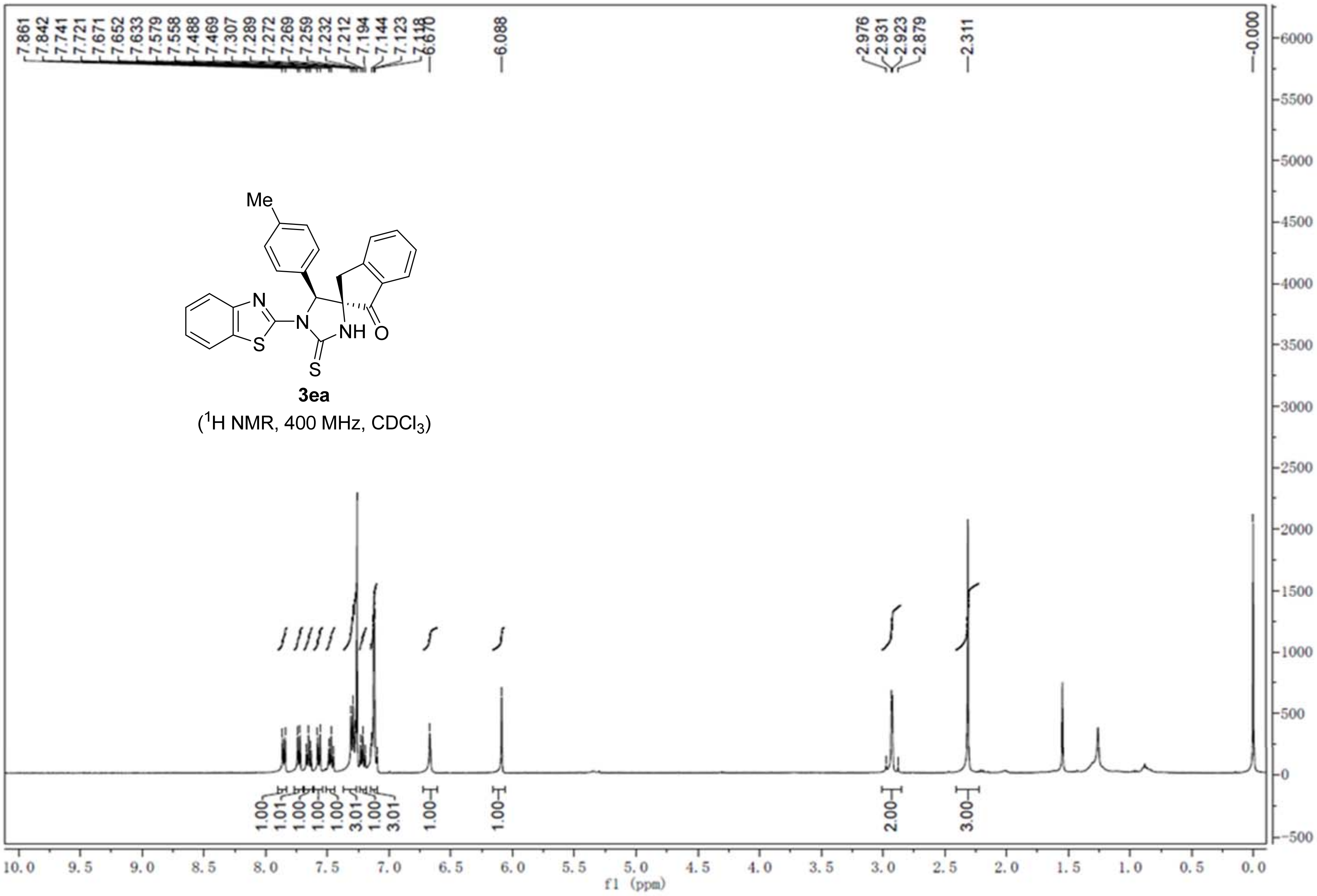
7.861
7.842
7.741
7.721
7.671
7.652
7.633
7.579
7.558
7.488
7.469
7.307
7.289
7.272
7.269
7.259
7.232
7.212
7.194
7.144
7.123
7.118
6.670
6.088
2.976
2.931
2.923
2.879
2.311
-0.000
Me
N
N
S
NH
O
S
3ea
(^{1}H NMR, 400 MHz, $CDCl_3$)
1.00
1.01
1.00
1.00
1.00
3.01
1.00
3.01
1.00
1.00
2.00
3.00
6000
5500
5000
4500
4000
3500
3000
2500
2000
1500
1000
500
0
-500
10.0 9.5 9.0 8.5 8.0 7.5 7.0 6.5 6.0 5.5 5.0 4.5 4.0 3.5 3.0 2.5 2.0 1.5 1.0 0.5 0.0
f1 (ppm)

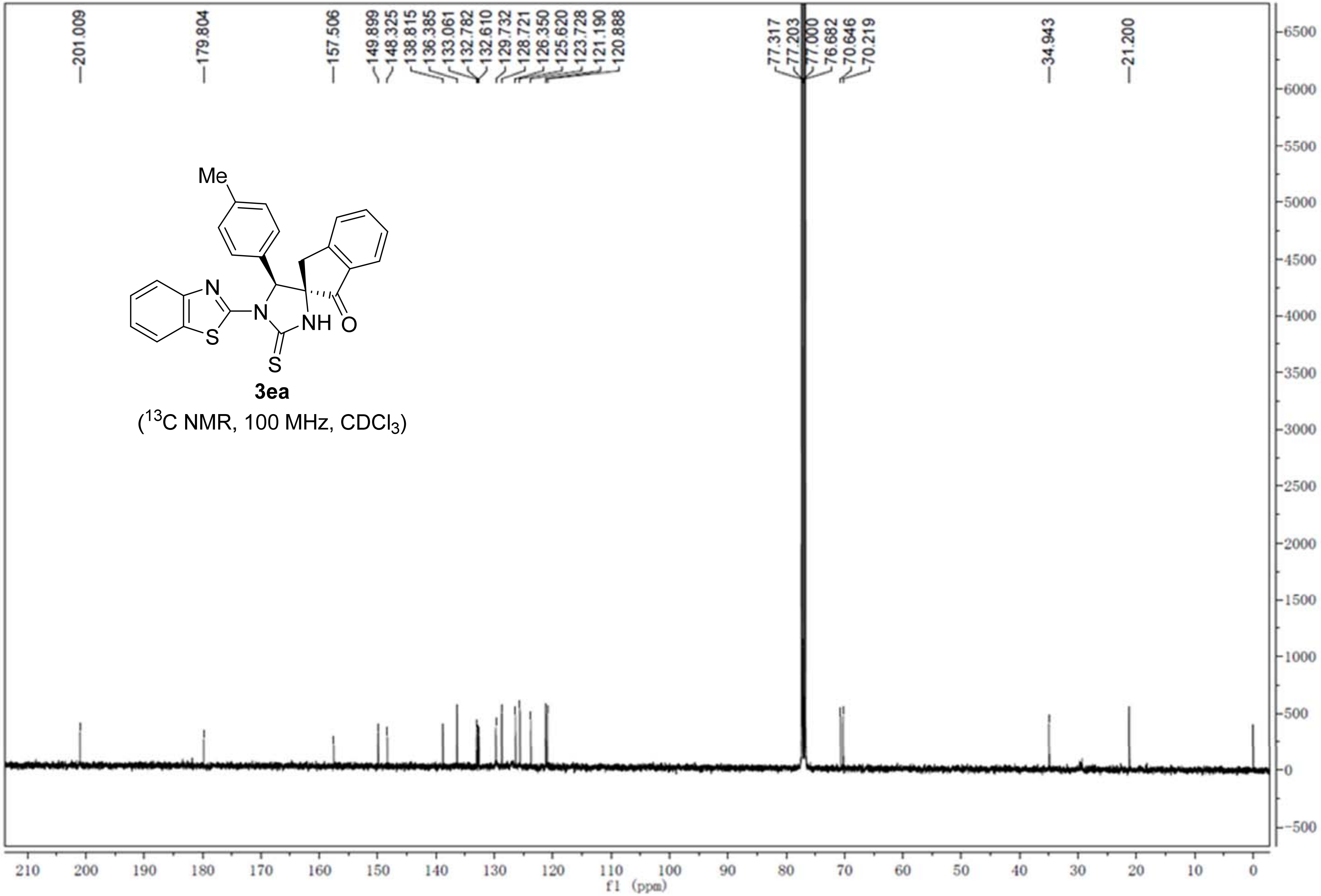

3ea

(^{13}C NMR, 100 MHz, $CDCl_3$)

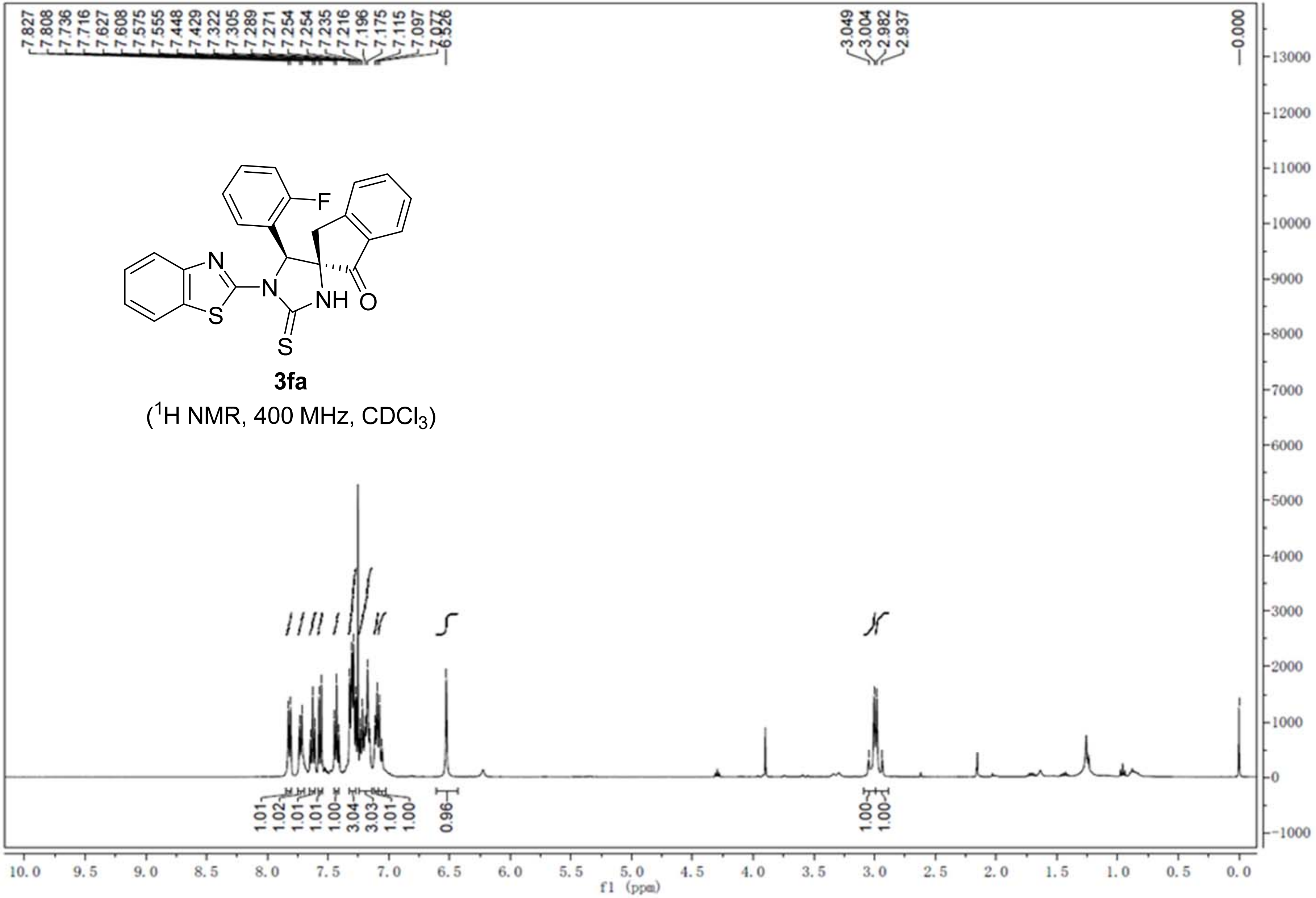

3fa

(^{1}H NMR, 400 MHz, $CDCl_3$)

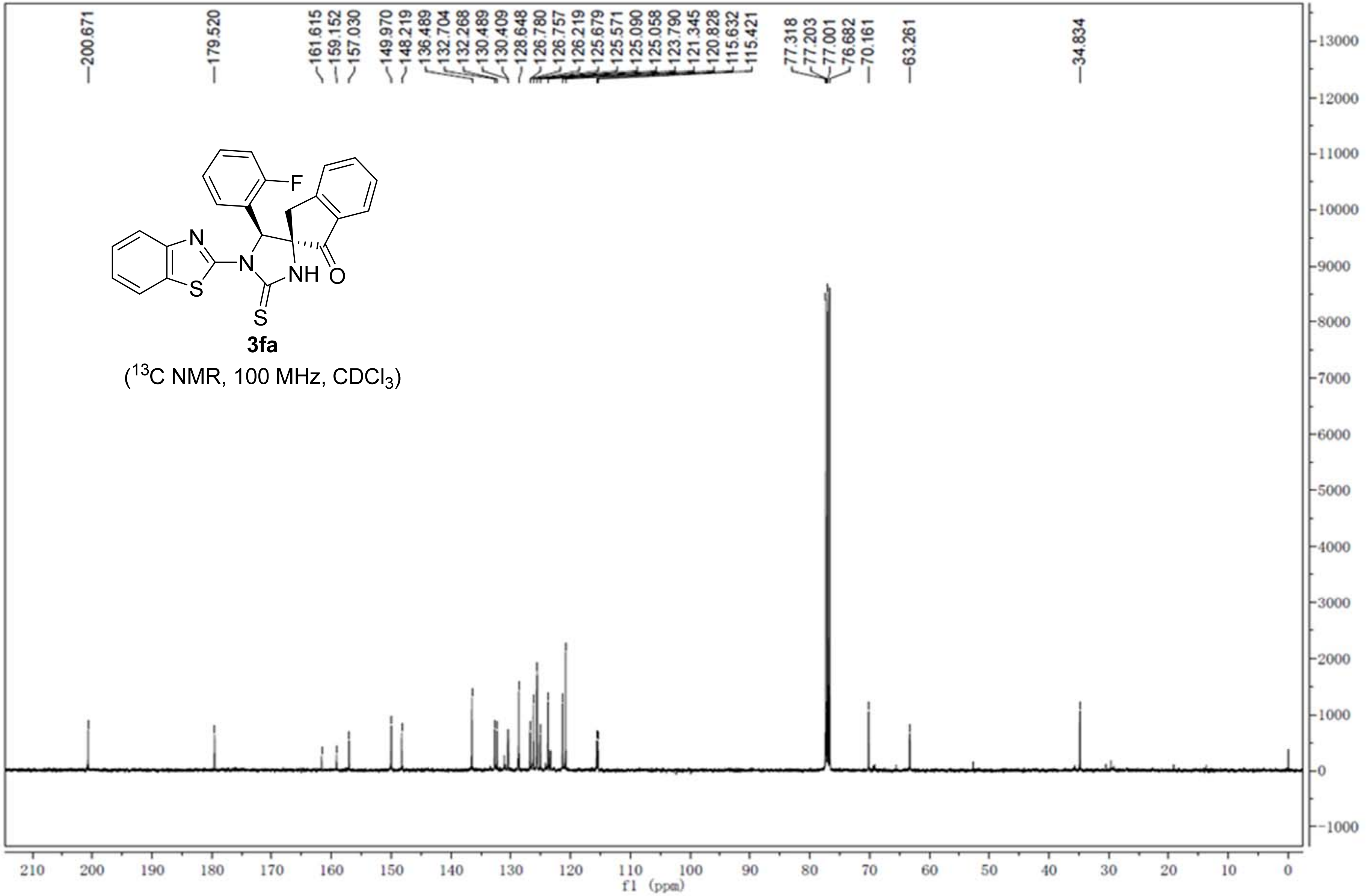

3fa

(^{13}C NMR, 100 MHz, $CDCl_3$)

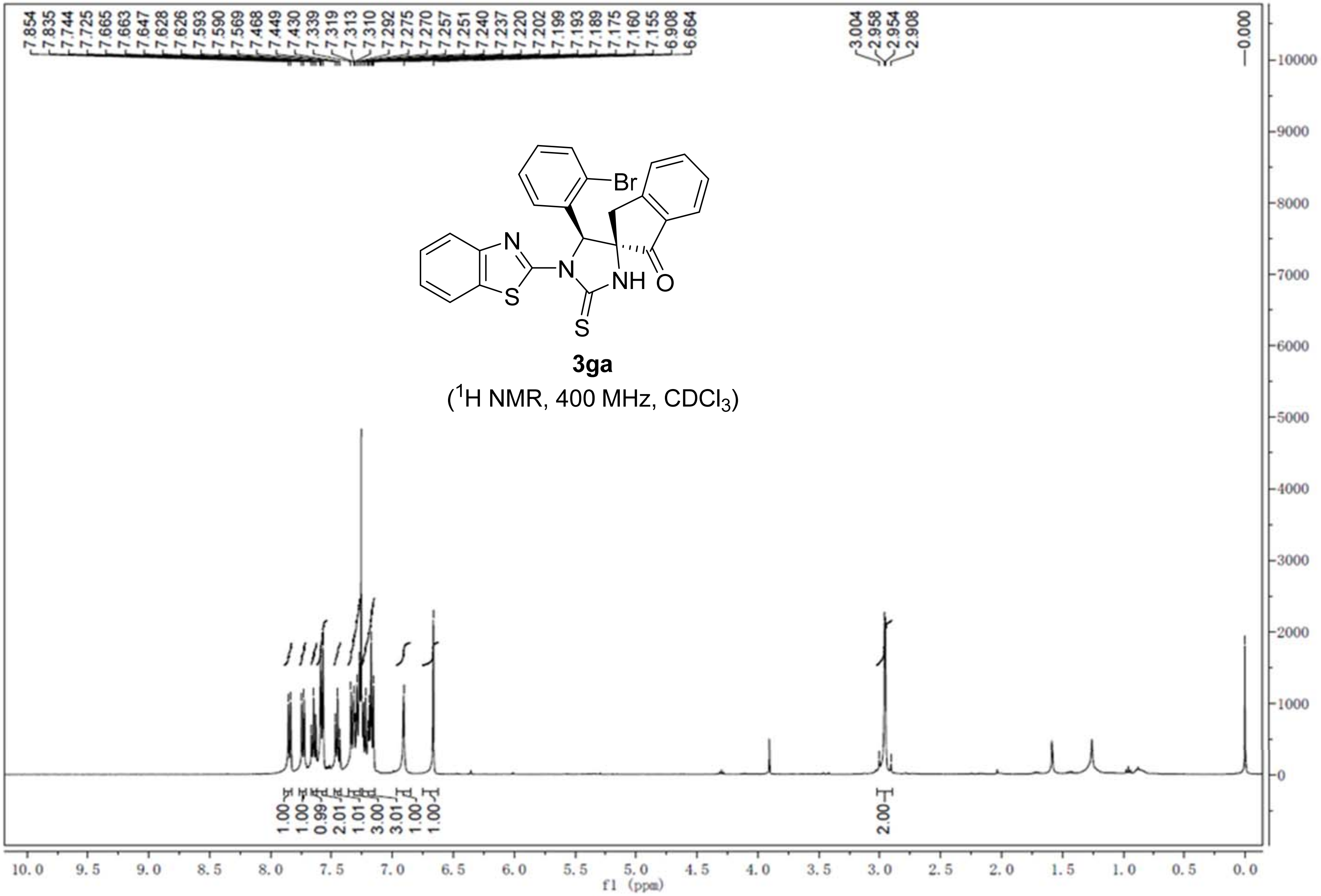

3ga

(^1H NMR, 400 MHz, $CDCl_3$)

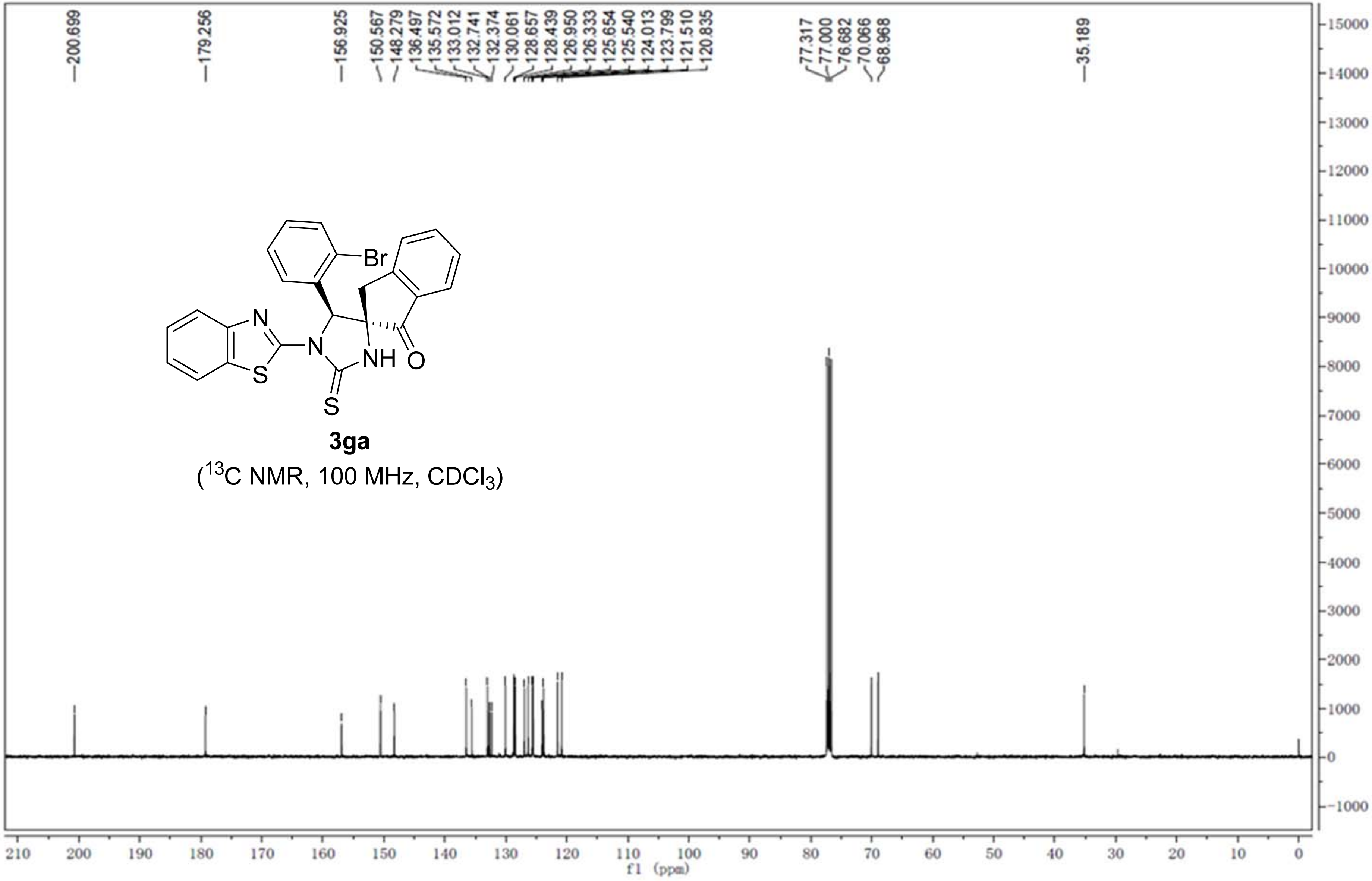

3ga

(^{13}C NMR, 100 MHz, $CDCl_3$)

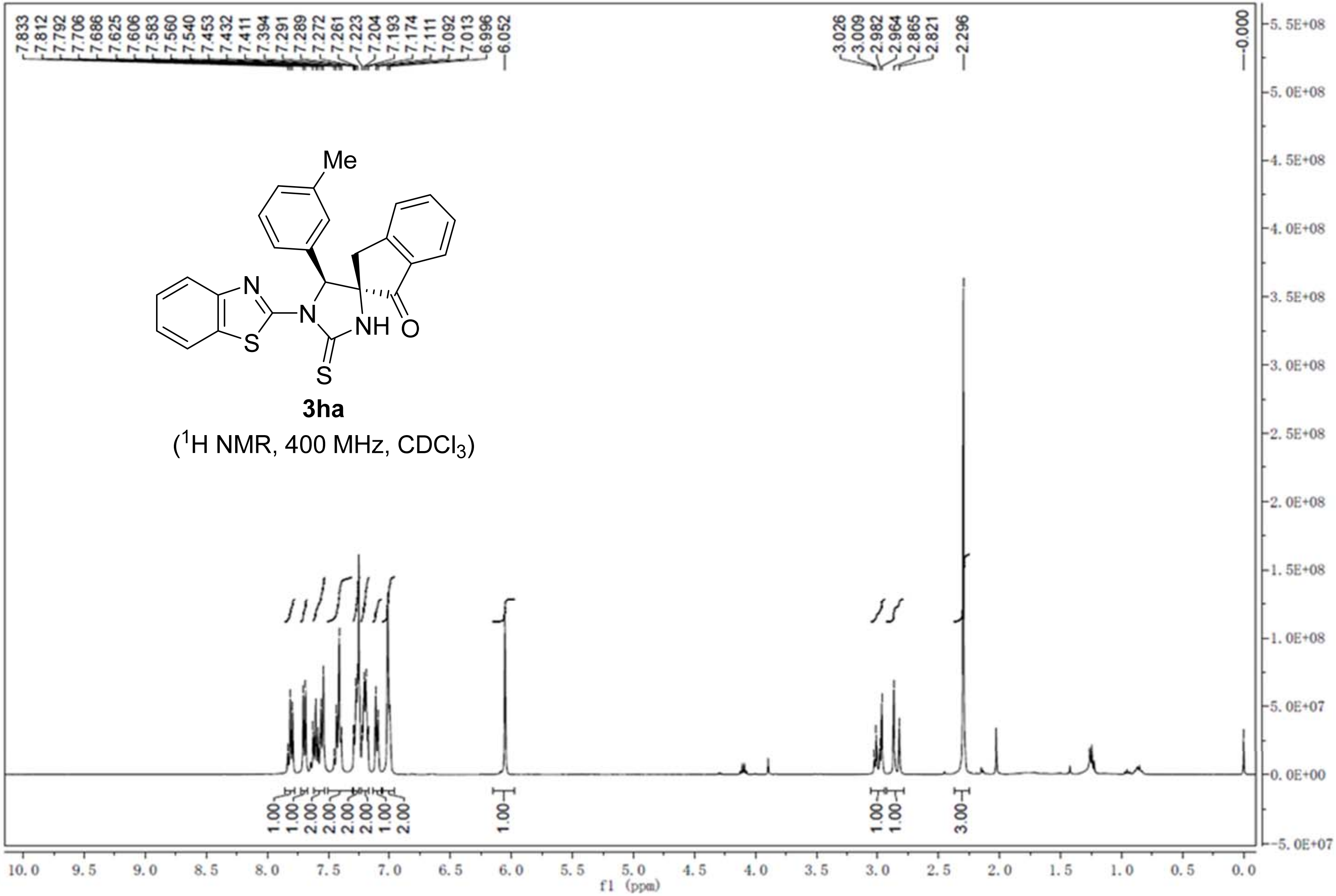

7.833
7.812
7.792
7.706
7.686
7.625
7.606
7.583
7.560
7.540
7.453
7.432
7.411
7.394
7.291
7.289
7.272
7.261
7.223
7.204
7.193
7.174
7.111
7.092
7.013
6.996
6.052
3.026
3.009
2.982
2.964
2.865
2.821
2.296
-0.000
Me
N
N
S
NH
O
S
3ha
(^{1}H NMR, 400 MHz, CDCl$_3$)
1.00
1.00
2.00
2.00
2.00
1.00
2.00
1.00
1.00
1.00
3.00
f1 (ppm)

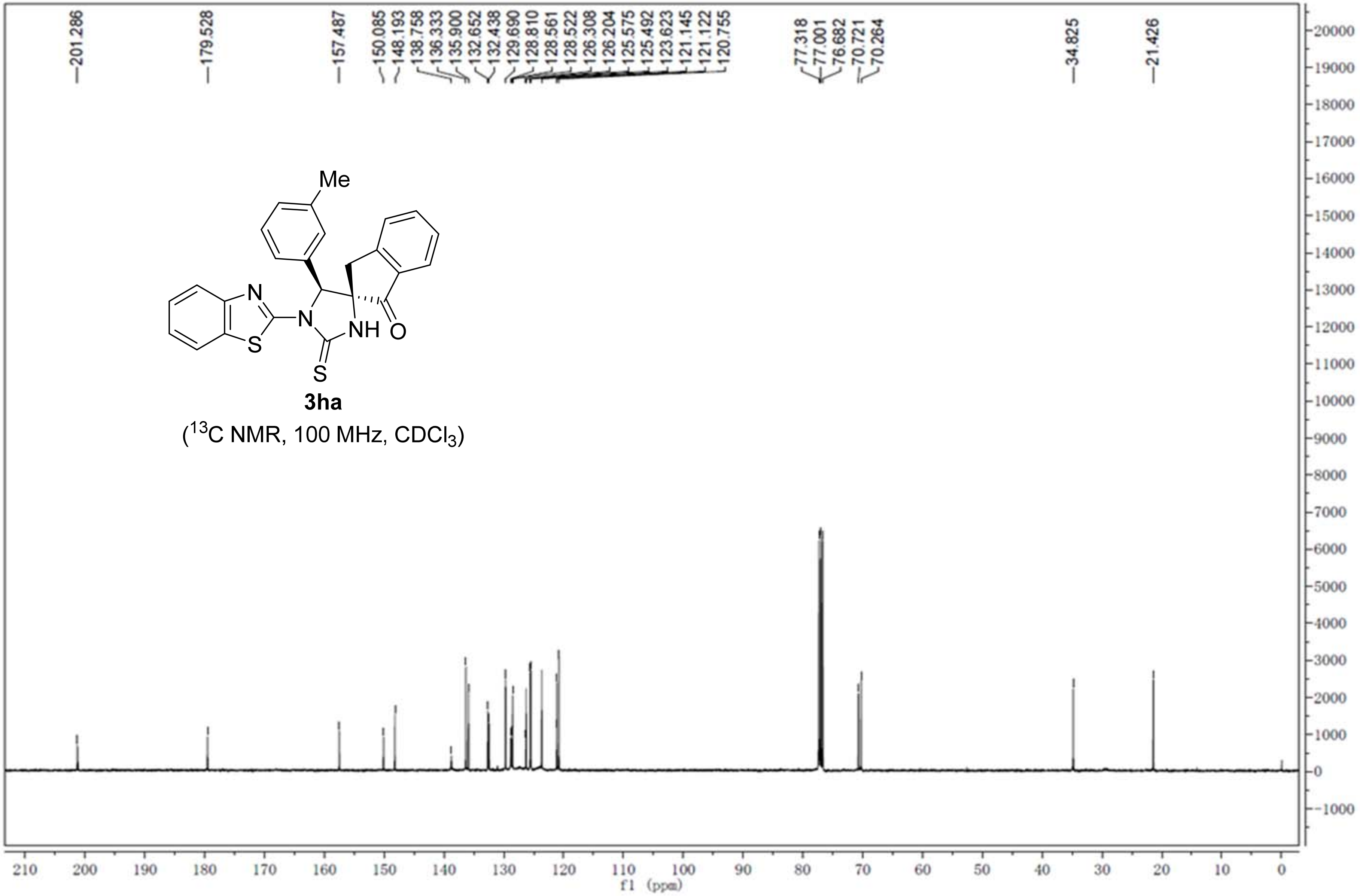

Me
N
N
S
NH
O
S
3ha
(^{13}C NMR, 100 MHz, $CDCl_3$)
201.286
179.528
157.487
150.085
148.193
138.758
136.333
135.900
132.652
132.438
129.690
128.810
128.561
128.522
126.308
126.204
125.575
125.492
123.623
121.145
121.122
120.755
77.318
77.001
76.682
70.721
70.264
34.825
21.426
f1 (ppm)

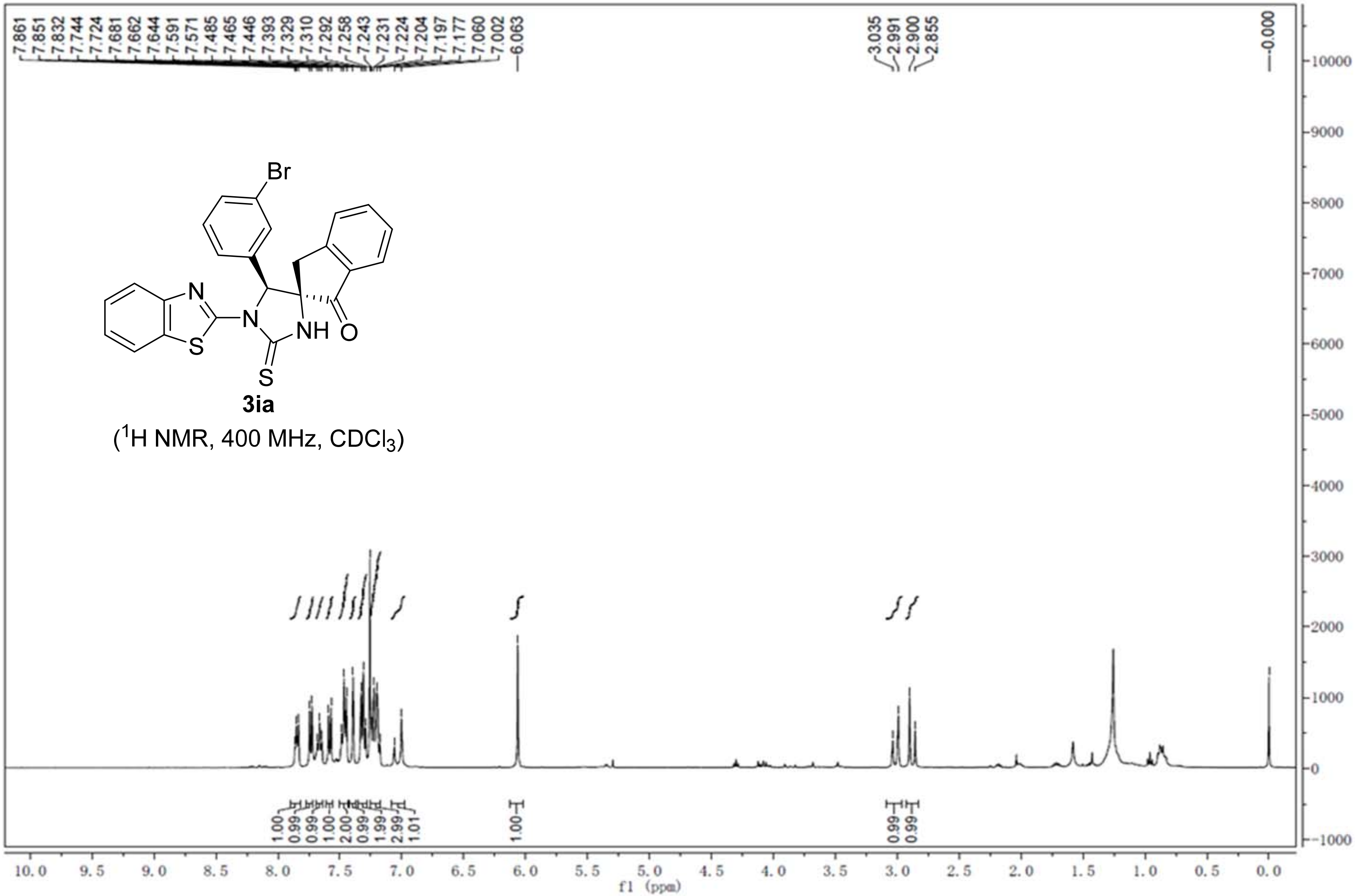

3ia

(^{1}H NMR, 400 MHz, $CDCl_3$)

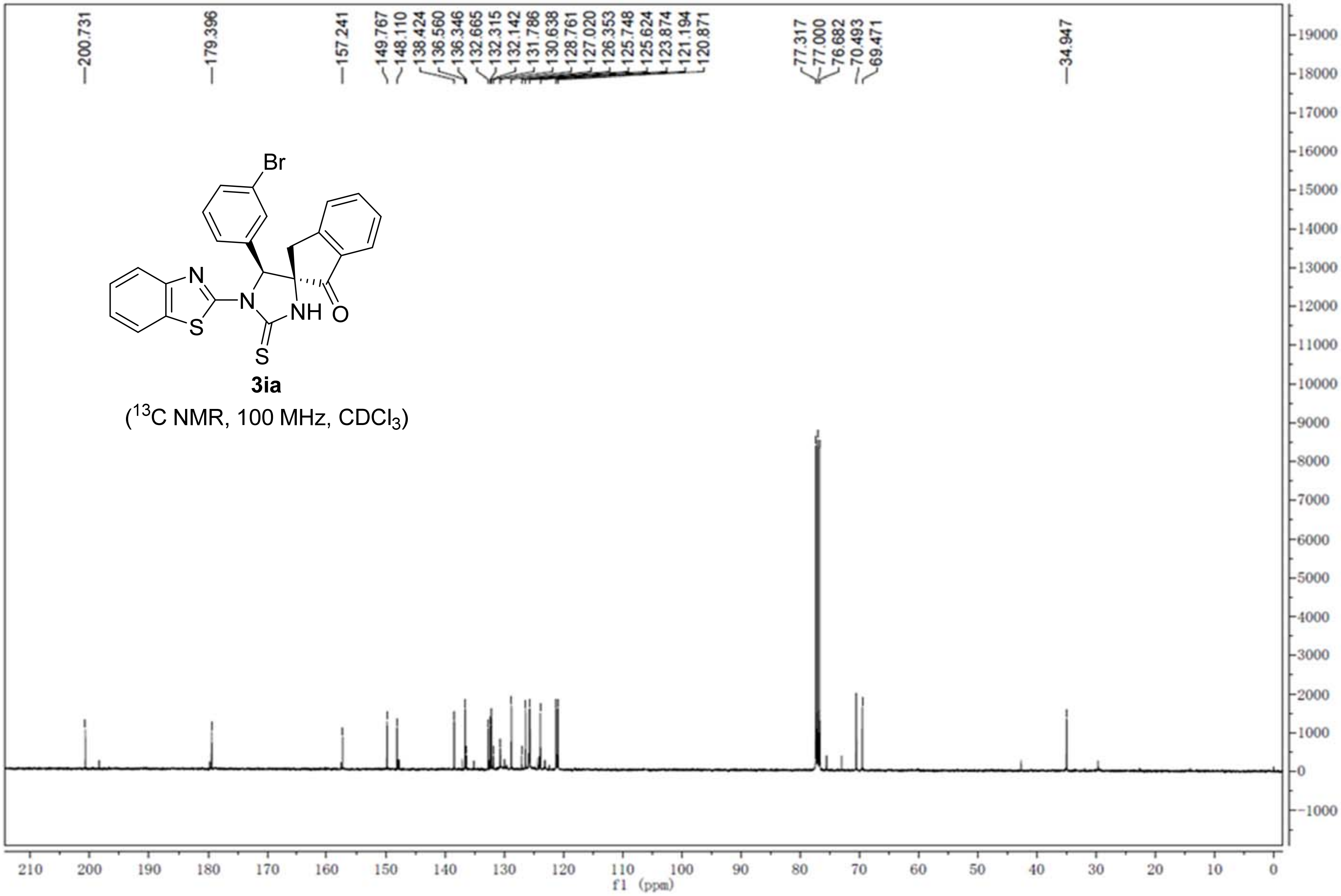

3ia

(^{13}C NMR, 100 MHz, $CDCl_3$)

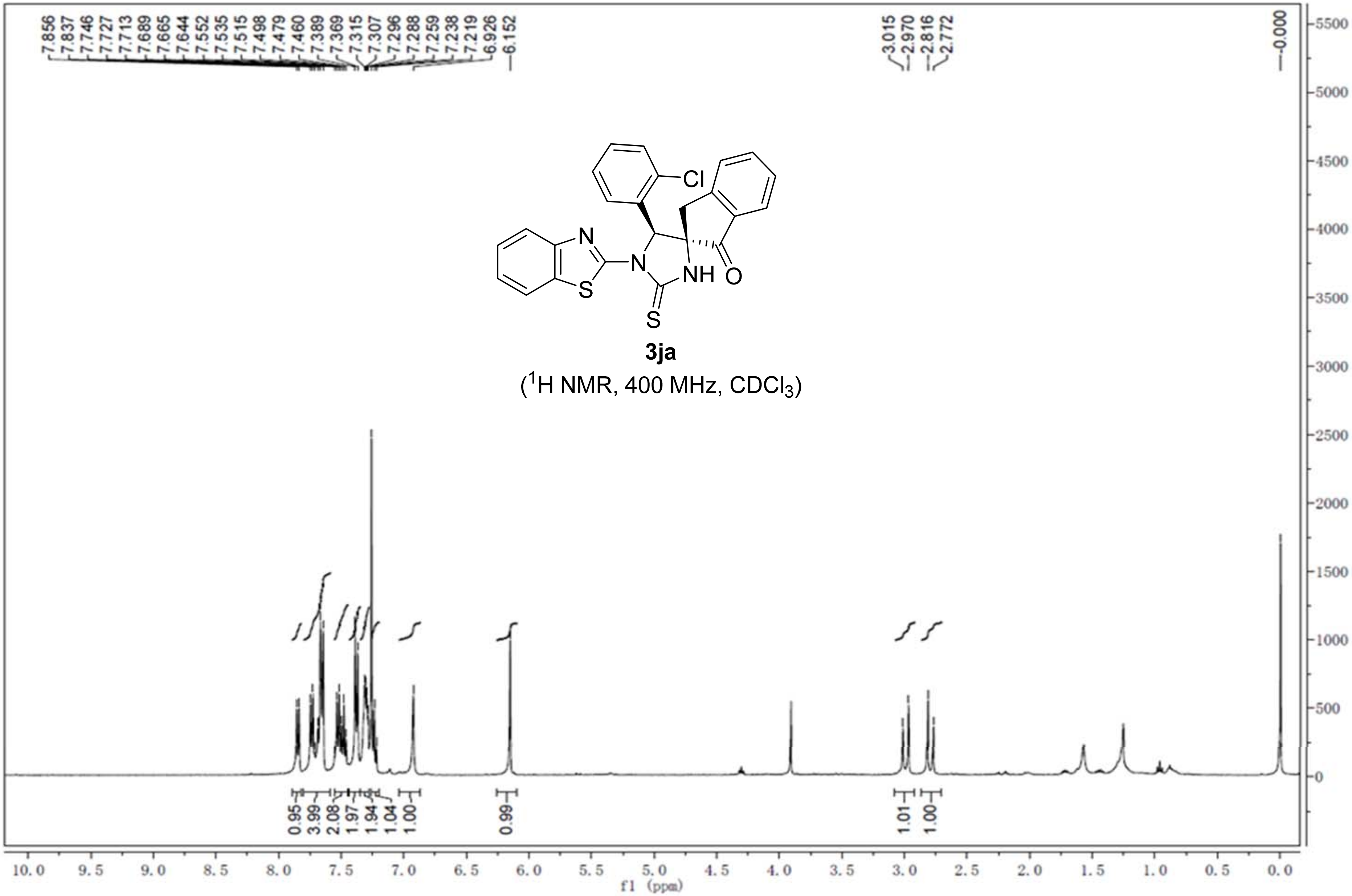
3ja
(^{1}H NMR, 400 MHz, $CDCl_3$)
f1 (ppm)

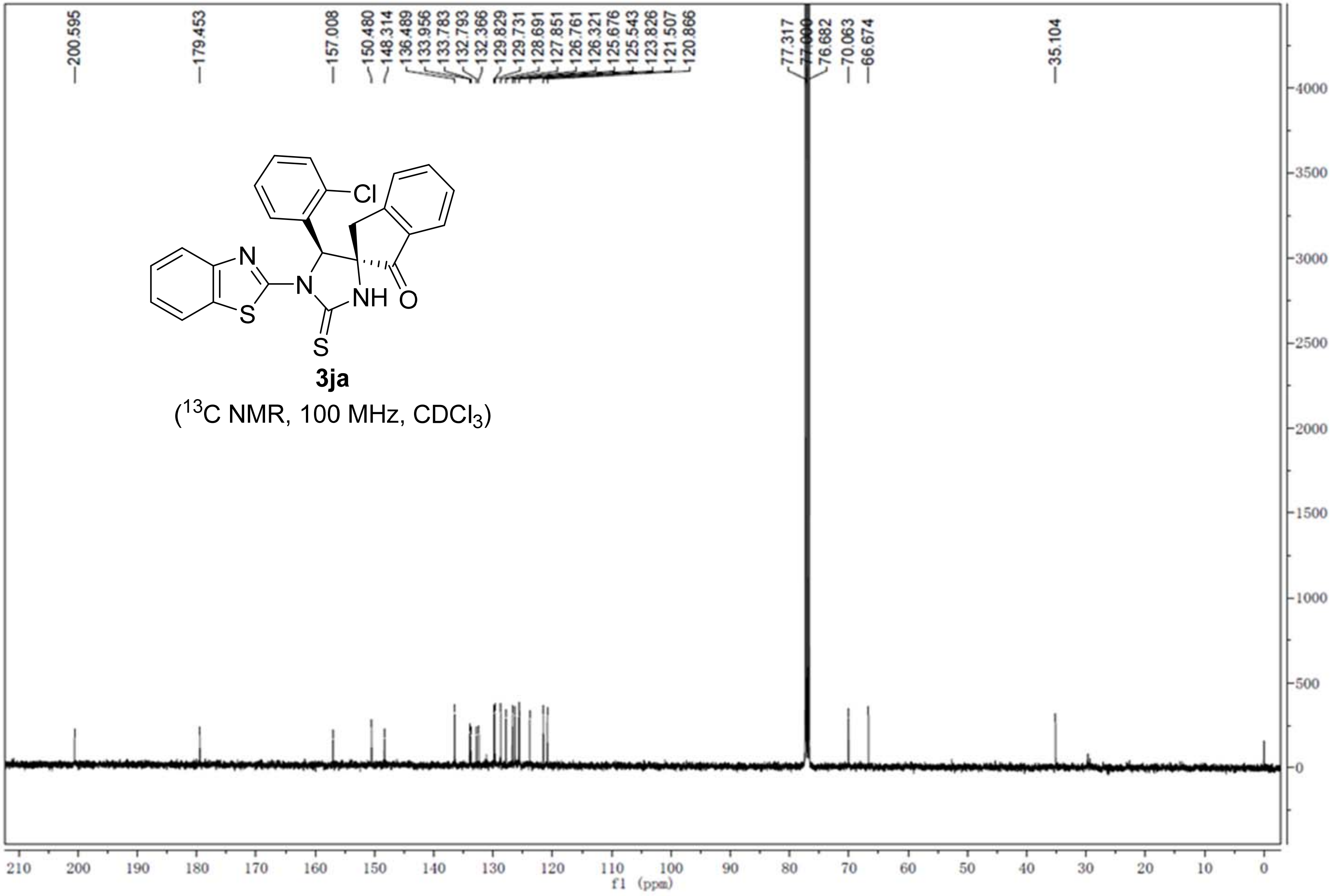
200.595
179.453
157.008
150.480
148.314
136.489
133.956
133.783
132.793
132.366
129.829
129.731
128.691
127.851
126.761
126.321
125.676
125.543
123.826
121.507
120.866
77.317
77.000
76.682
70.063
66.674
35.104
Cl
N
S
NH
O
3ja
(^{13}C NMR, 100 MHz, CDCl$_3$)
f1 (ppm)

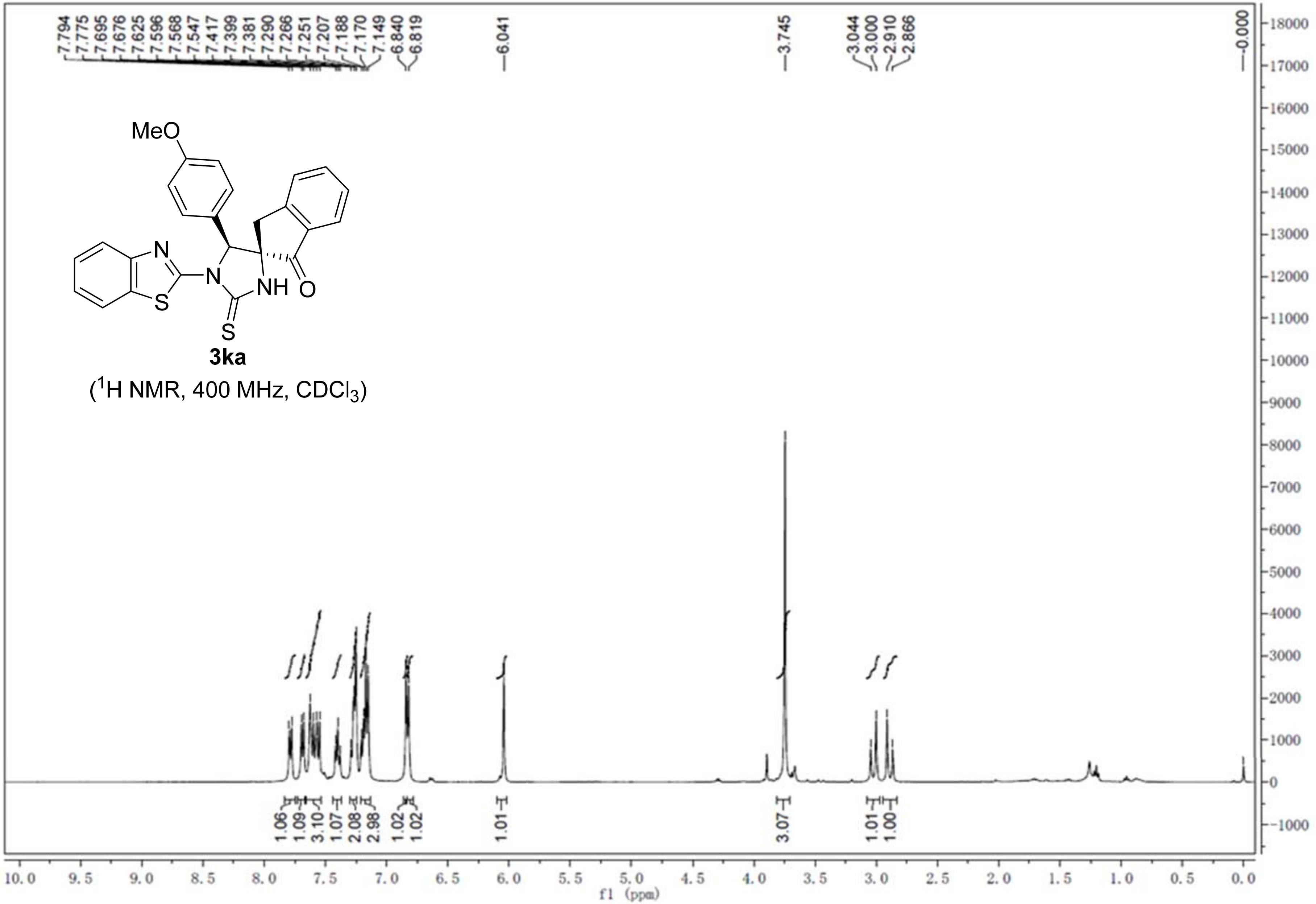

MeO
N
N
S
NH
O
S
3ka
(1H NMR, 400 MHz, CDCl3)
7.794
7.775
7.695
7.676
7.625
7.596
7.568
7.547
7.417
7.399
7.381
7.290
7.266
7.251
7.207
7.188
7.170
7.149
6.840
6.819
6.041
3.745
3.044
3.000
2.910
2.866
0.000
1.06
1.09
3.10
1.07
2.08
2.98
1.02
1.02
1.01
3.07
1.01
1.00
f1 (ppm)

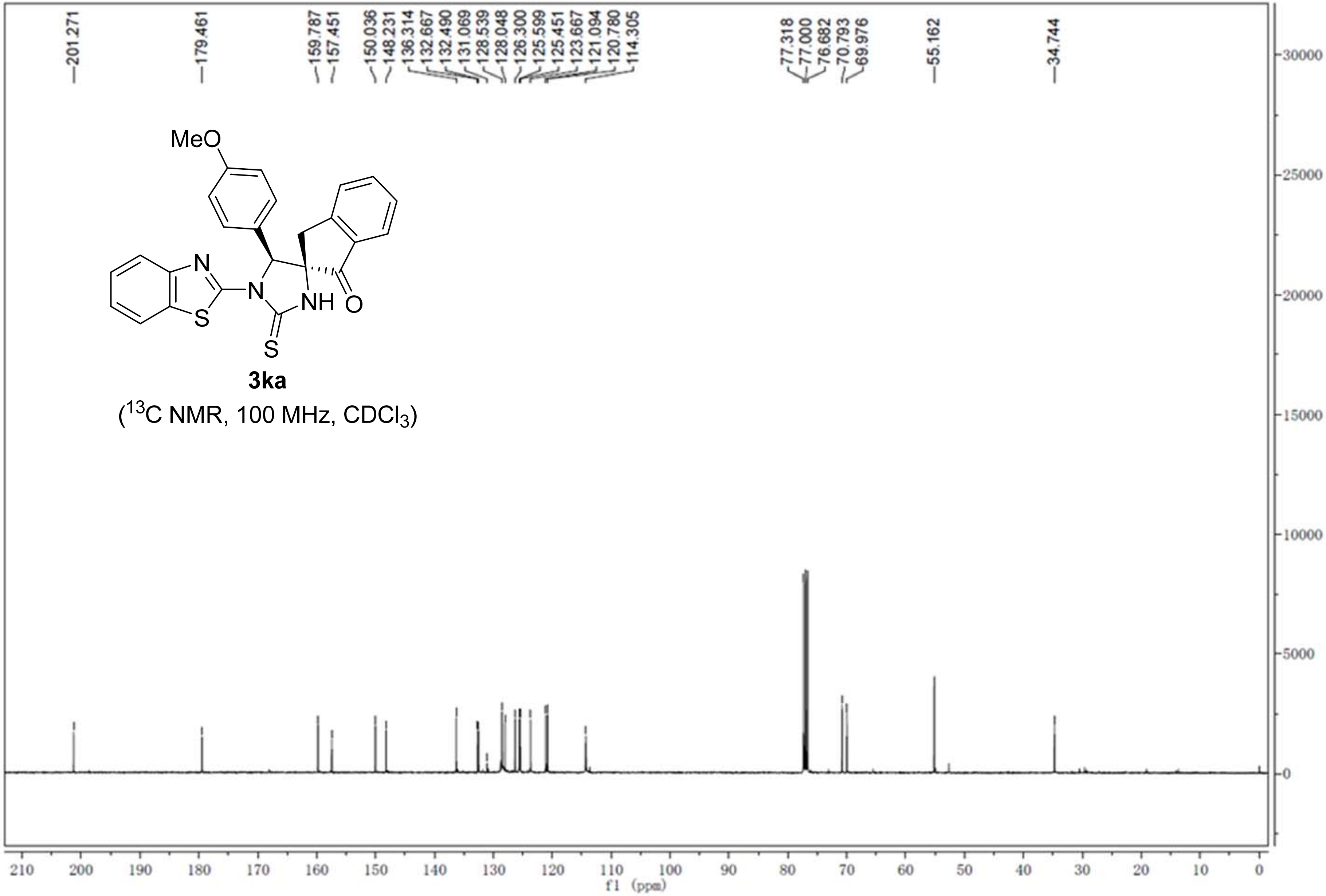

3ka

(^{13}C NMR, 100 MHz, $CDCl_3$)

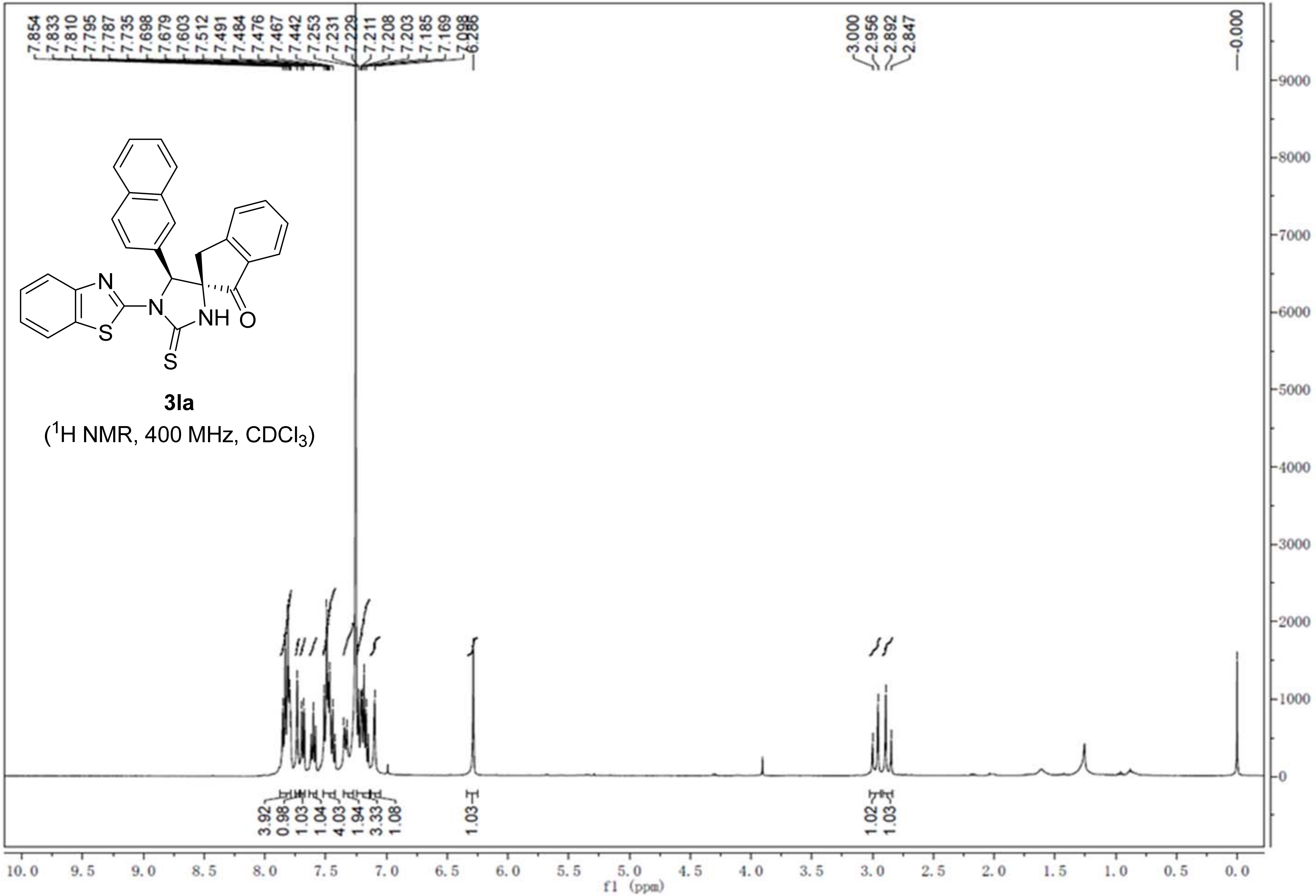
3la
(1H NMR, 400 MHz, CDCl3)
7.854 7.833 7.810 7.795 7.787 7.735 7.698 7.679 7.603 7.512 7.491 7.484 7.476 7.467 7.442 7.253 7.231 7.229 7.211 7.208 7.203 7.185 7.169 7.098 6.286
3.000 2.956 2.892 2.847
0.000
3.92 0.98 1.03 1.04 4.03 1.94 3.33 1.08
1.03
1.02 1.03
f1 (ppm)

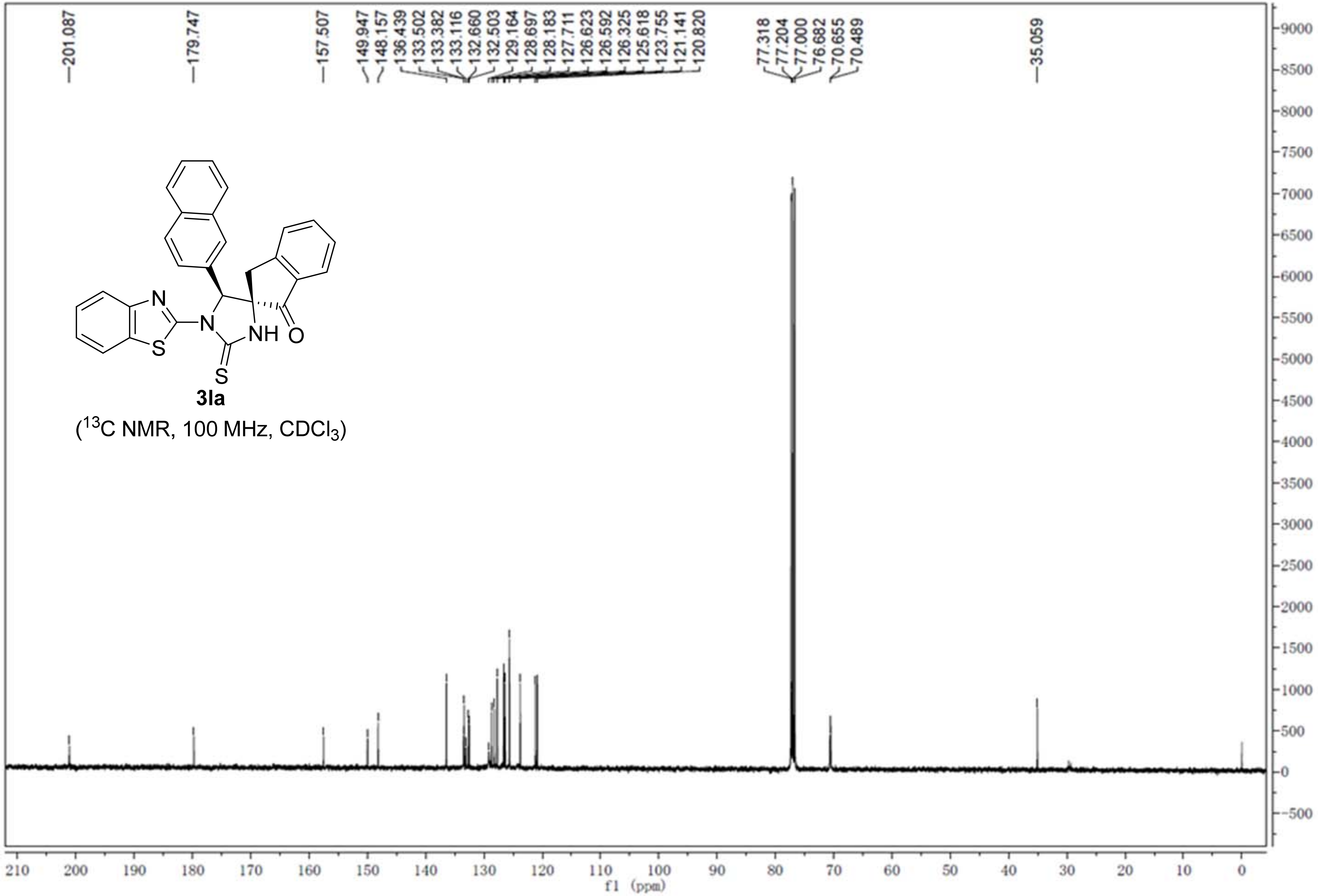
201.087
179.747
157.507
149.947
148.157
136.439
133.502
133.382
133.116
132.660
132.503
129.164
128.697
128.183
127.711
126.623
126.592
126.325
125.618
123.755
121.141
120.820
77.318
77.204
77.000
76.682
70.655
70.489
35.059
3la
(^{13}C NMR, 100 MHz, CDCl$_3$)
f1 (ppm)

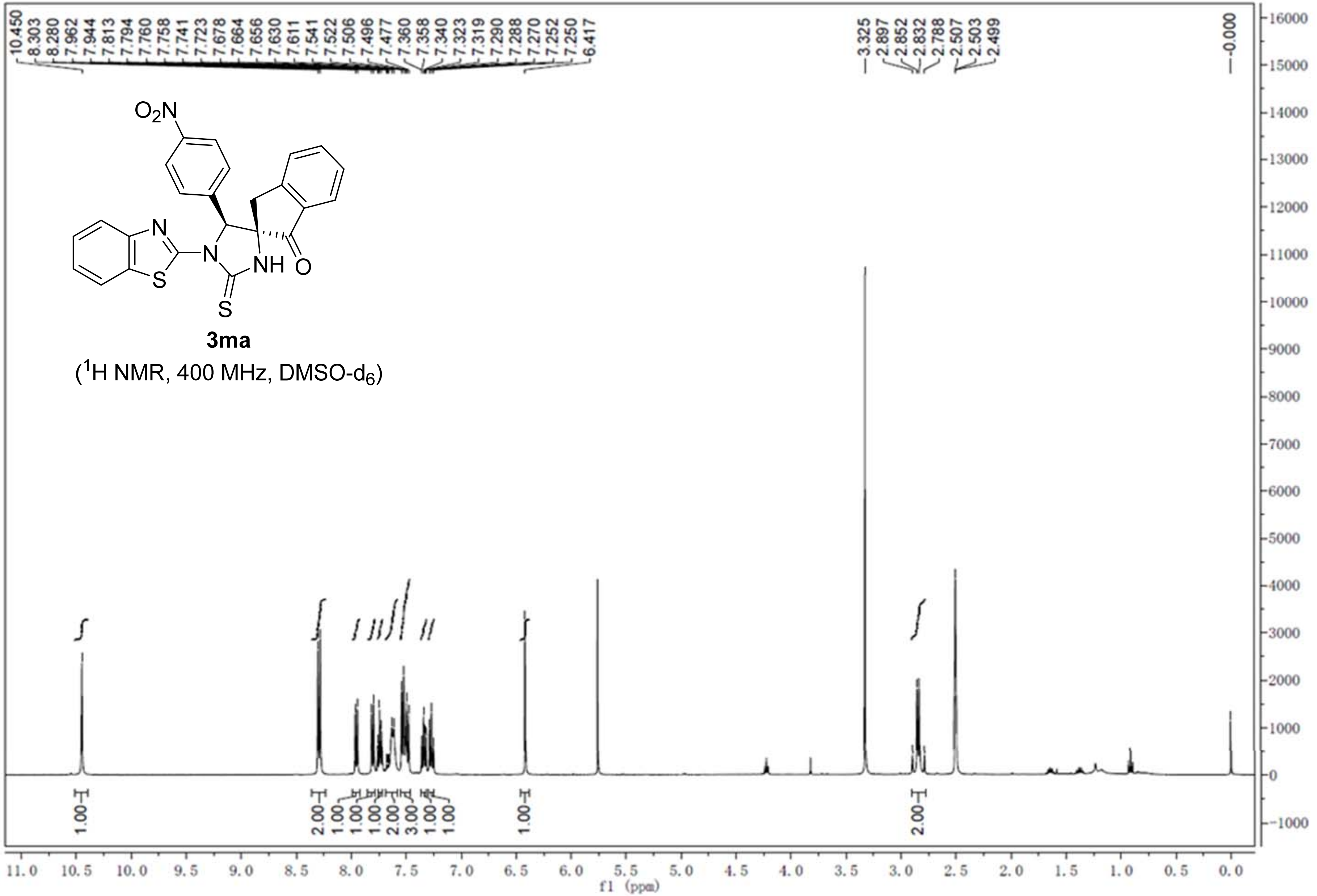

3ma

(^{1}H NMR, 400 MHz, DMSO-d_6)

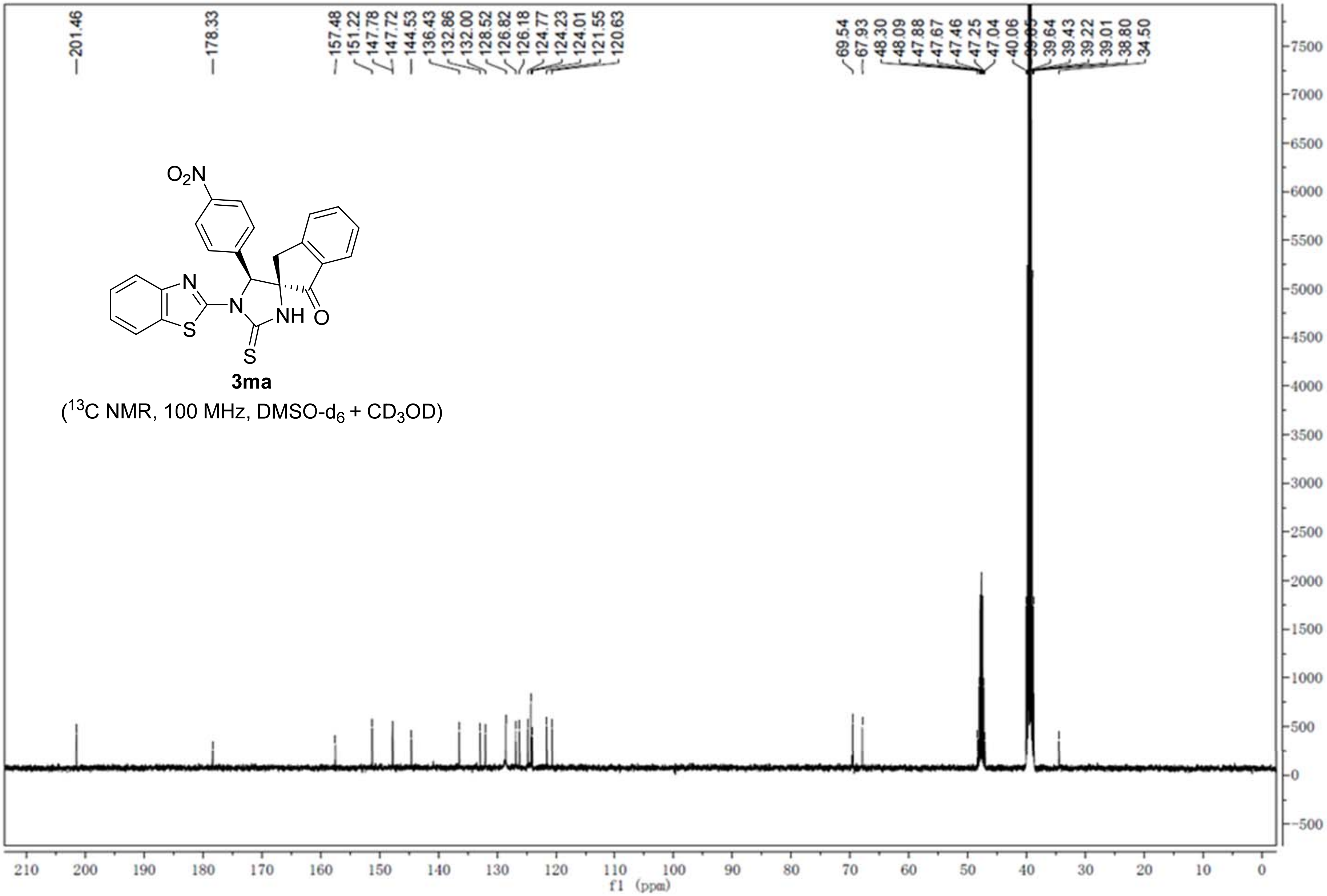

3ma

(^{13}C NMR, 100 MHz, DMSO-d_6 + CD_3OD)

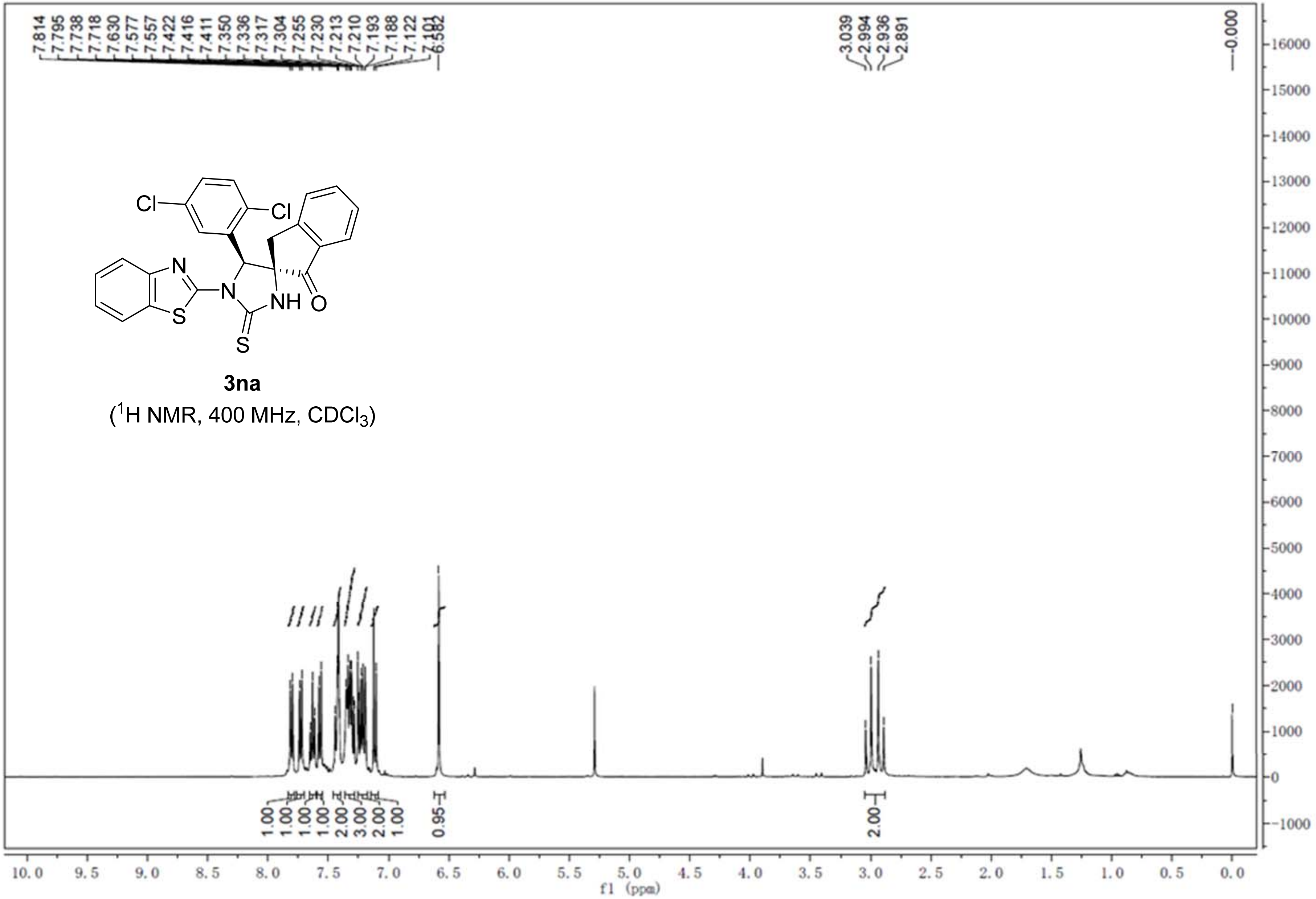

7.814
7.795
7.738
7.718
7.630
7.577
7.557
7.422
7.416
7.411
7.350
7.336
7.317
7.304
7.255
7.230
7.213
7.210
7.193
7.188
7.122
7.101
6.582
3.039
2.994
2.936
2.891
0.000
Cl
Cl
N
N
S
NH
O
S
3na
(^{1}H NMR, 400 MHz, CDCl$_3$)
1.00
1.00
1.00
1.00
2.00
3.00
2.00
1.00
0.95
2.00
f1 (ppm)

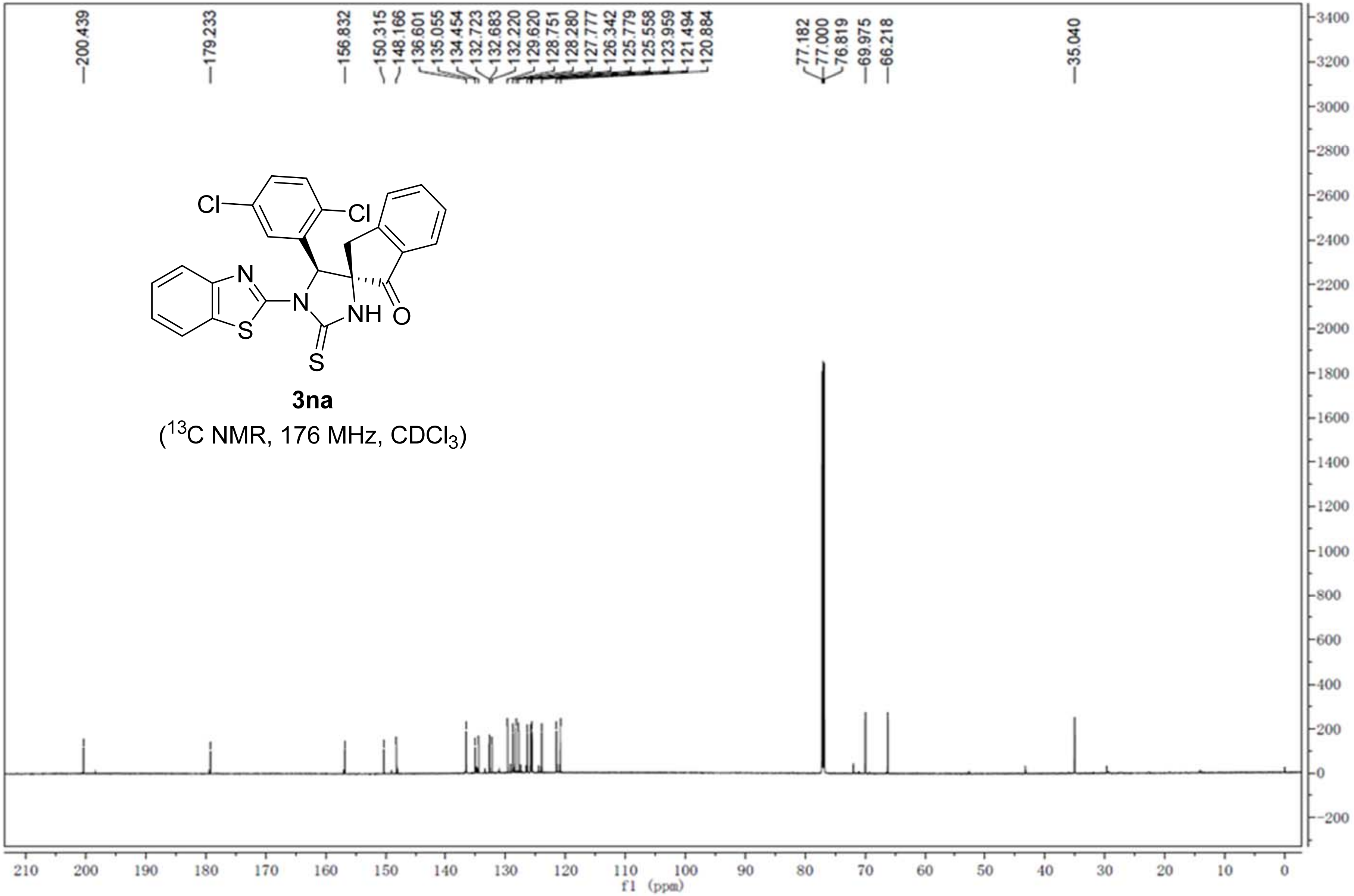

3na

(^{13}C NMR, 176 MHz, $CDCl_3$)

2. X-ray single-crystal data for product 3ab

The single crystal of **3ab** was cultured in a mixture of petroleum ether and ethyl acetate, the single crystal data see Table S1.

Table S1 Crystal data and structure refinement for **3ab**

Identification code	CCDC 2346984
Empirical formula	$C_{25}H_{19}N_3O_2S_2$
Formula weight	457.55
Temperature/K	296.15
Crystal system	monoclinic
Space group	P21
a/Å	9.79193(14)
b/Å	10.91195(14)
c/Å	11.23258(15)
α/°	90
β/°	111.0557(16)
γ/°	90
Volume/Å^3	1120.06(3)
Z	2
ρ calcg/cm^3	1.357
μ /mm^{-1}	2.380
F(000)	476.0
Crystal size/mm^3	0.22 × 0.20 × 0.15
Radiation	CuKα (λ = 1.54184)
2Θ range for data collection/°	8.436 to 152.382
Index ranges	-12 ≤ h ≤ 12, -11 ≤ k ≤ 13, -14 ≤ 1 ≤ 14
Reflections collected	19967
Independent reflections	4362 [R_{int} = 0.0387, R_{sigma} = 0.0283]
Data/restraints/parameters	4362/1/291
Goodness-of-fit on F2	1.069
Final R indexes [I>=2σ (I)]	R_1 = 0.0274, wR_2 = 0.0724
Final R indexes [all data]	R_1 = 0.0289, wR_2 = 0.0735
Largest diff. peak/hole / e Å^{-3}	0.14/-0.12
Flack parameter	-0.010(7)

3. Copies of HPLC chromatograms

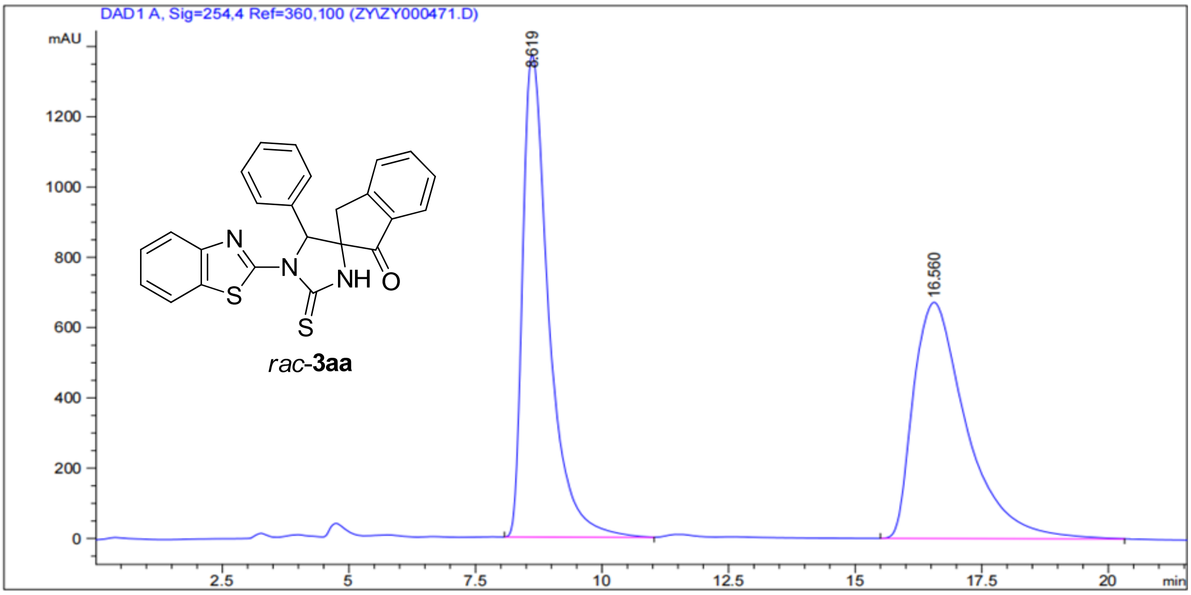

Peak #	RetTime [min]	Type	Width [min]	Area [mAU*s]	Height [mAU]	Area %
1	8.619	BB	0.5388	4.89980e4	1371.61890	49.9605
2	16.560	BB	1.1155	4.90755e4	672.37659	50.0395

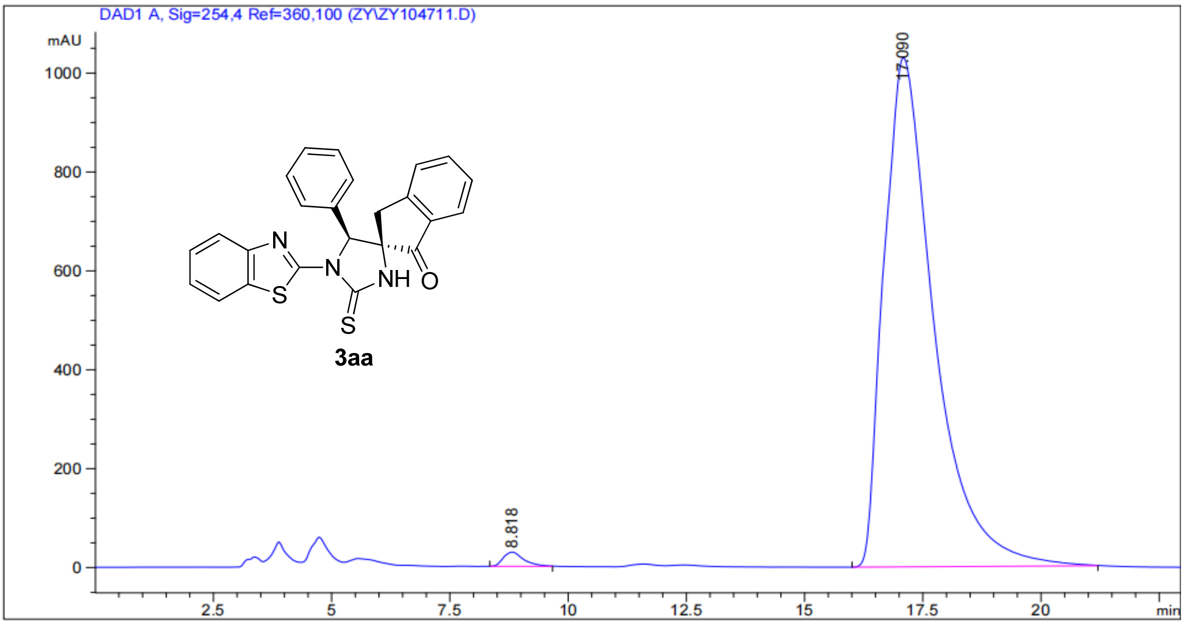

Peak #	RetTime [min]	Type	Width [min]	Area [mAU*s]	Height [mAU]	Area %
1	8.818	BB	0.4583	863.86151	28.77445	1.1245
2	17.090	BB	1.0386	7.59549e4	1029.71899	98.8755

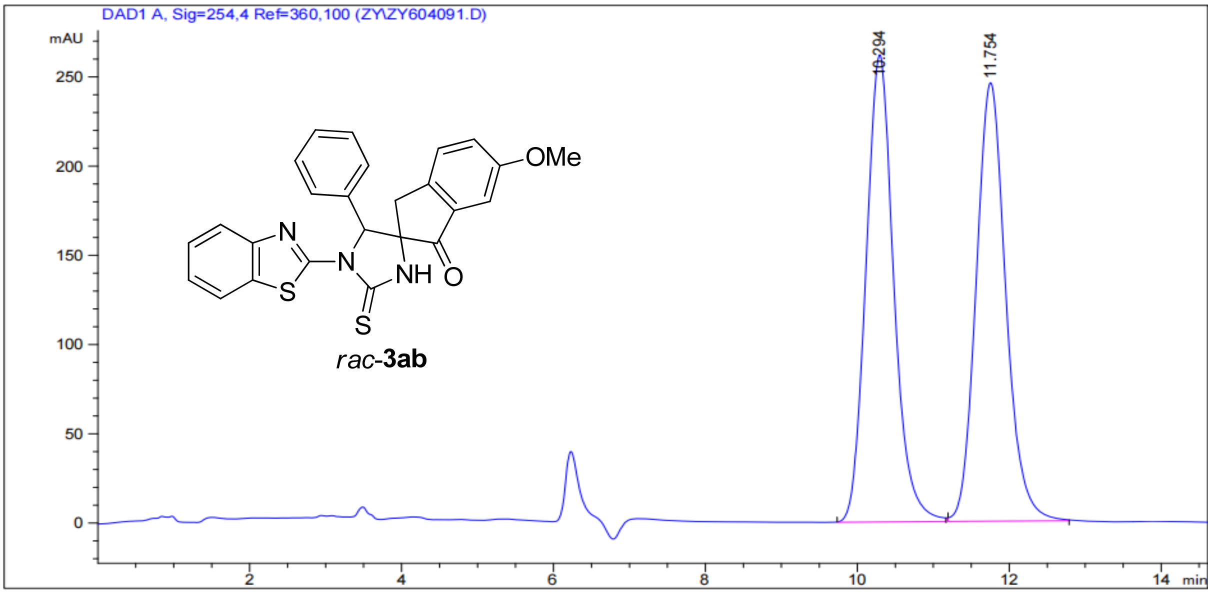

Peak #	RetTime [min]	Type	Width [min]	Area [mAU*s]	Height [mAU]	Area %
1	10.294	BB	0.3876	6634.37061	261.81299	49.9128
2	11.754	BB	0.4161	6657.56396	245.71350	50.0872

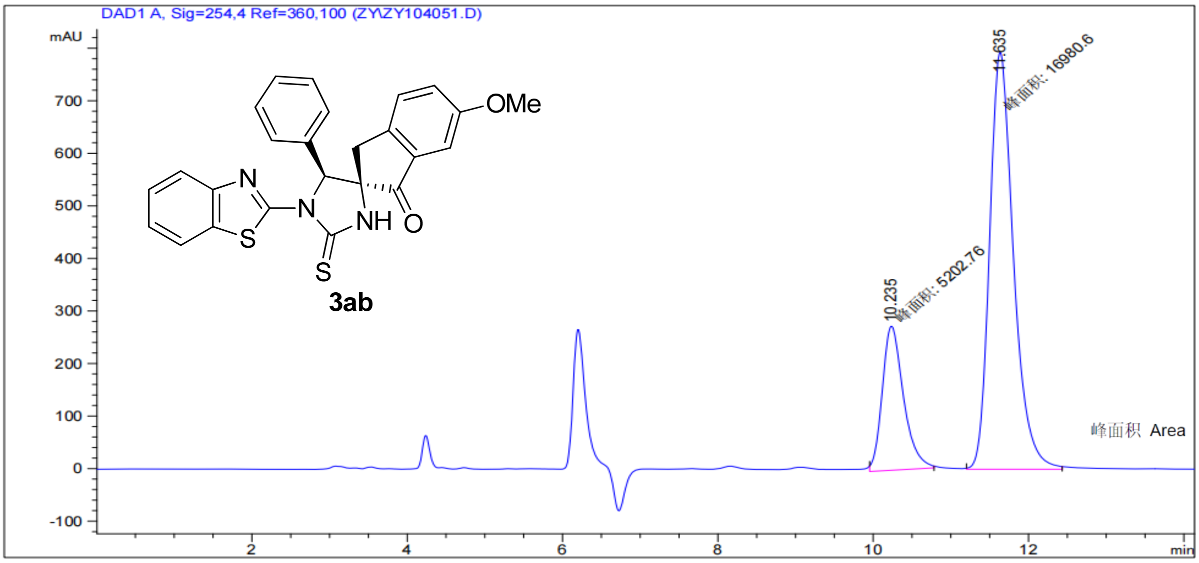

Peak #	RetTime [min]	Type	Width [min]	Area [mAU*s]	Height [mAU]	Area %
1	10.235	MM	0.3163	5202.75537	274.17947	23.4534
2	11.635	MM	0.3565	1.69806e4	793.90430	76.5466

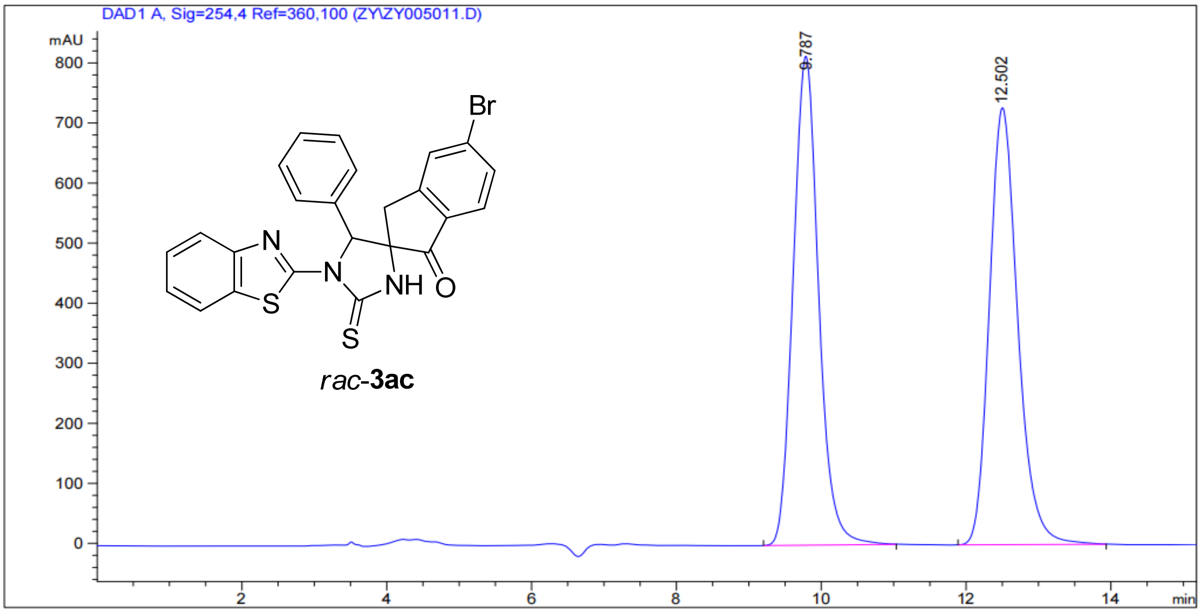

Peak #	RetTime [min]	Type	Width [min]	Area [mAU*s]	Height [mAU]	Area %
1	9.787	BB	0.3765	1.98554e4	814.08441	50.0252
2	12.502	BB	0.4201	1.98354e4	727.53284	49.9748

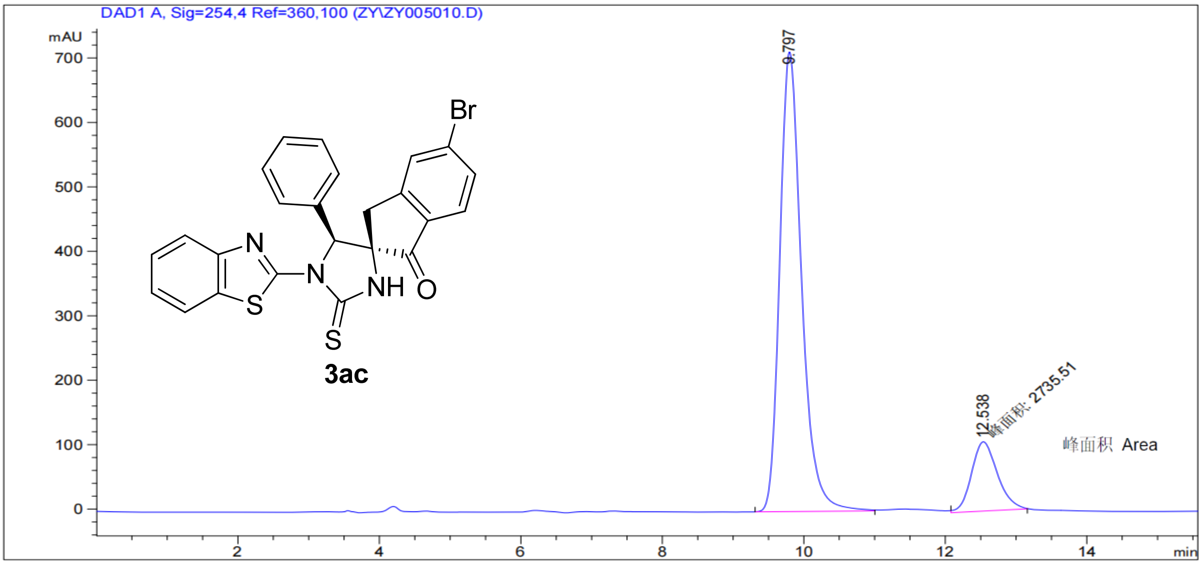

Peak #	RetTime [min]	Type	Width [min]	Area [mAU*s]	Height [mAU]	Area %
1	9.797	BB	0.3280	1.52824e4	713.23334	84.8179
2	12.538	MM	0.4242	2735.51147	107.48721	15.1821

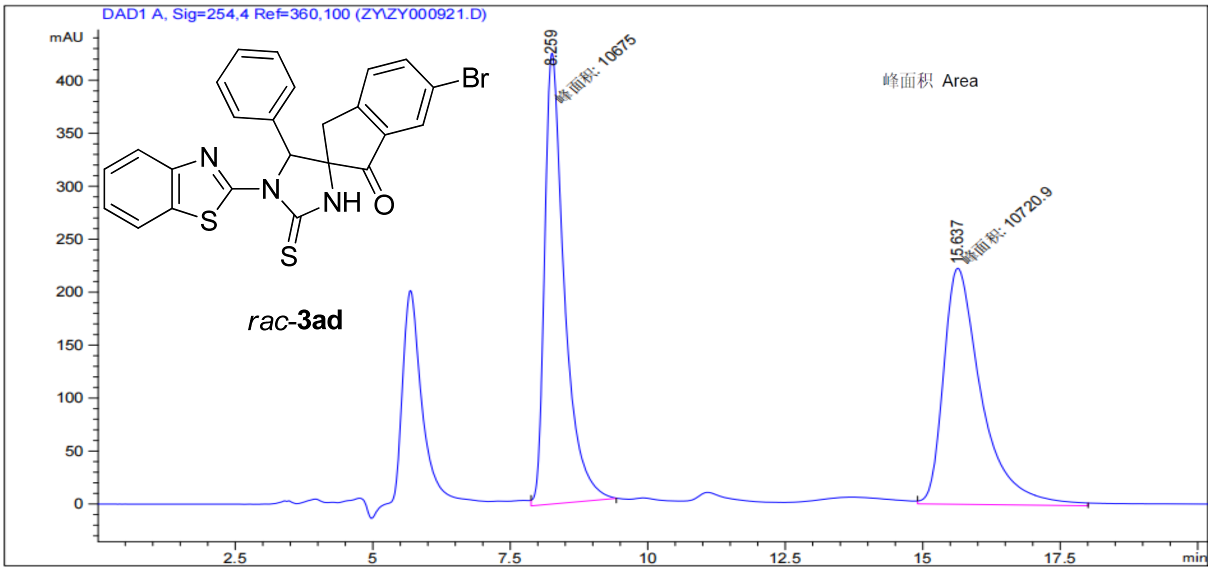

Peak #	RetTime [min]	Type	Width [min]	Area [mAU*s]	Height [mAU]	Area %
1	8.259	MM	0.4178	1.06750e4	425.81555	49.8927
2	15.637	MM	0.8016	1.07209e4	222.91397	50.1073

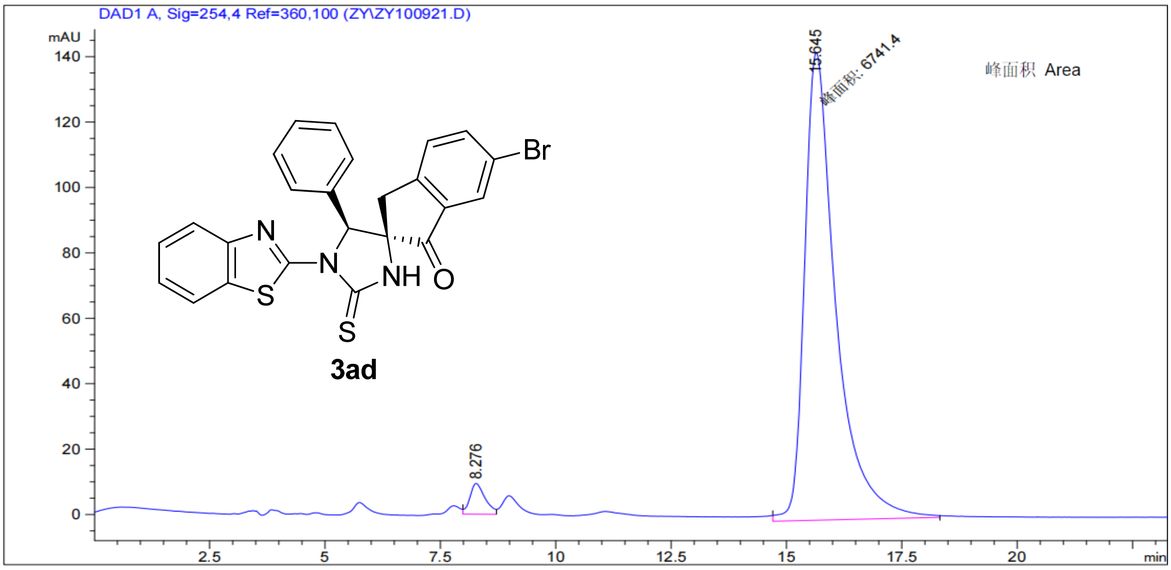

Peak #	RetTime [min]	Type	Width [min]	Area [mAU*s]	Height [mAU]	Area %
1	8.276	VV	0.3373	215.28442	9.39692	3.0946
2	15.645	MM	0.7860	6741.39648	142.95525	96.9054

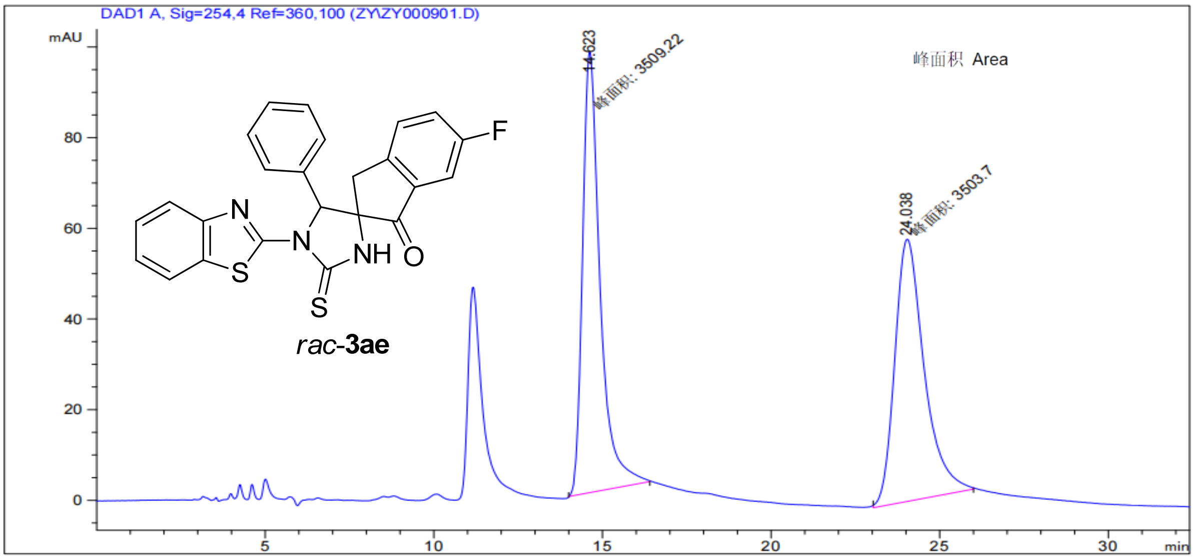

Peak #	RetTime [min]	Type	Width [min]	Area [mAU*s]	Height [mAU]	Area %
1	14.623	MM	0.6017	3509.21509	97.20454	50.0393
2	24.038	MM	1.0098	3503.69995	57.83085	49.9607

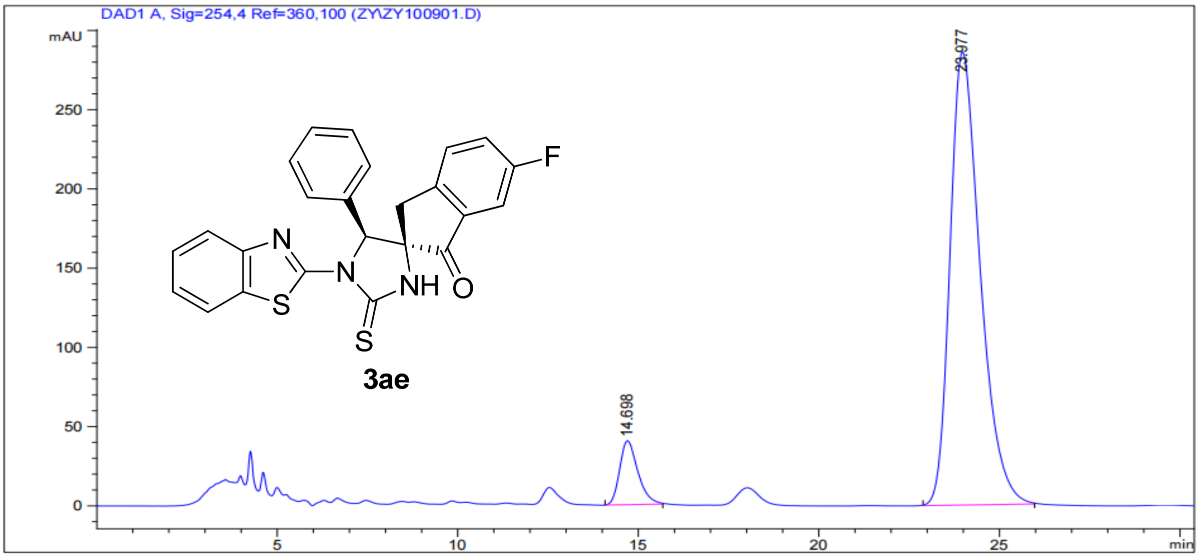

Peak #	RetTime [min]	Type	Width [min]	Area [mAU*s]	Height [mAU]	Area %
1	14.698	BB	0.5343	1413.52380	40.38930	7.8392
2	23.977	BB	0.8840	1.66180e4	285.93530	92.1608

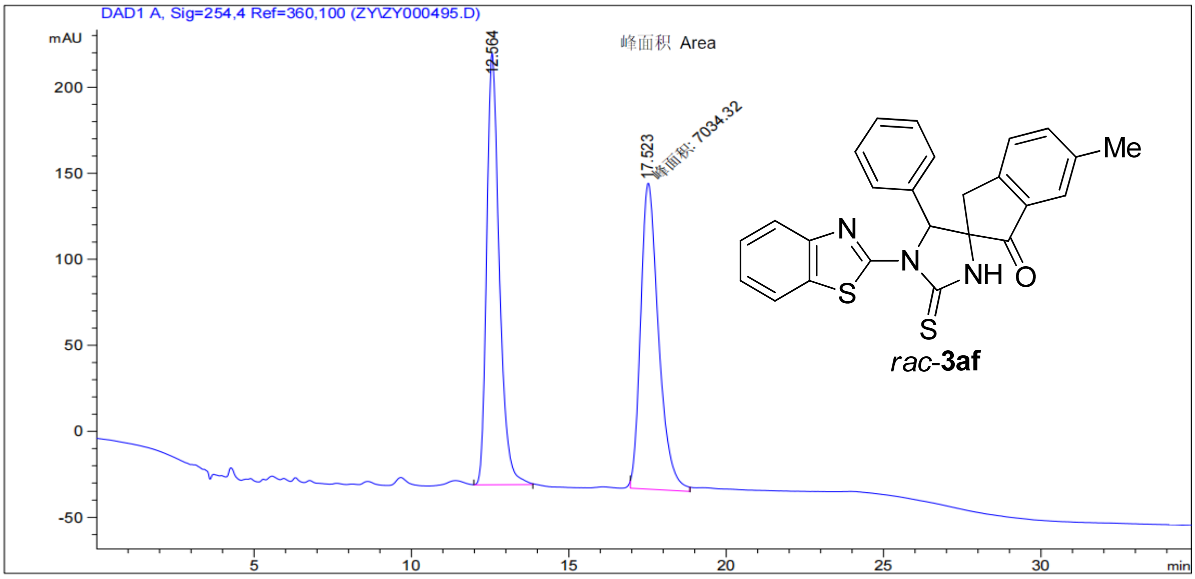

Peak #	RetTime [min]	Type	Width [min]	Area [mAU*s]	Height [mAU]	Area %
1	12.564	VB	0.4242	7008.11426	250.61877	49.9067
2	17.523	MM	0.6593	7034.32471	177.82365	50.0933

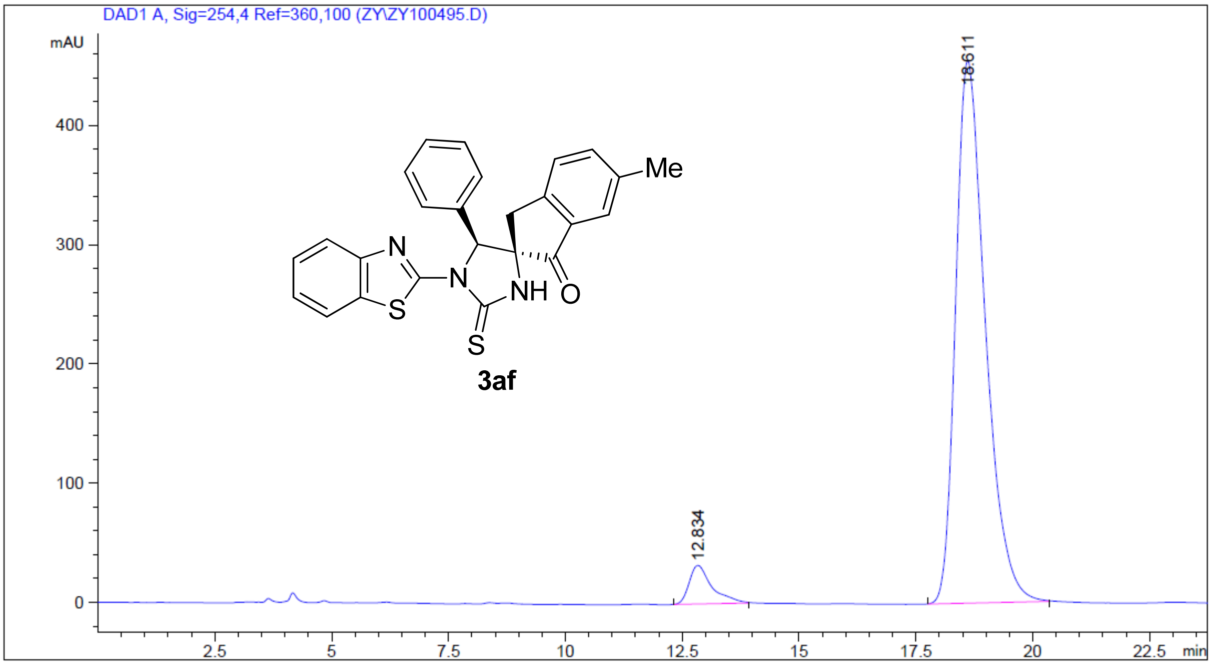

Peak #	RetTime [min]	Type	Width [min]	Area [mAU*s]	Height [mAU]	Area %
1	12.834	BB	0.4926	1099.14575	32.49813	5.1862
2	18.611	BB	0.6694	2.00947e4	454.82361	94.8138

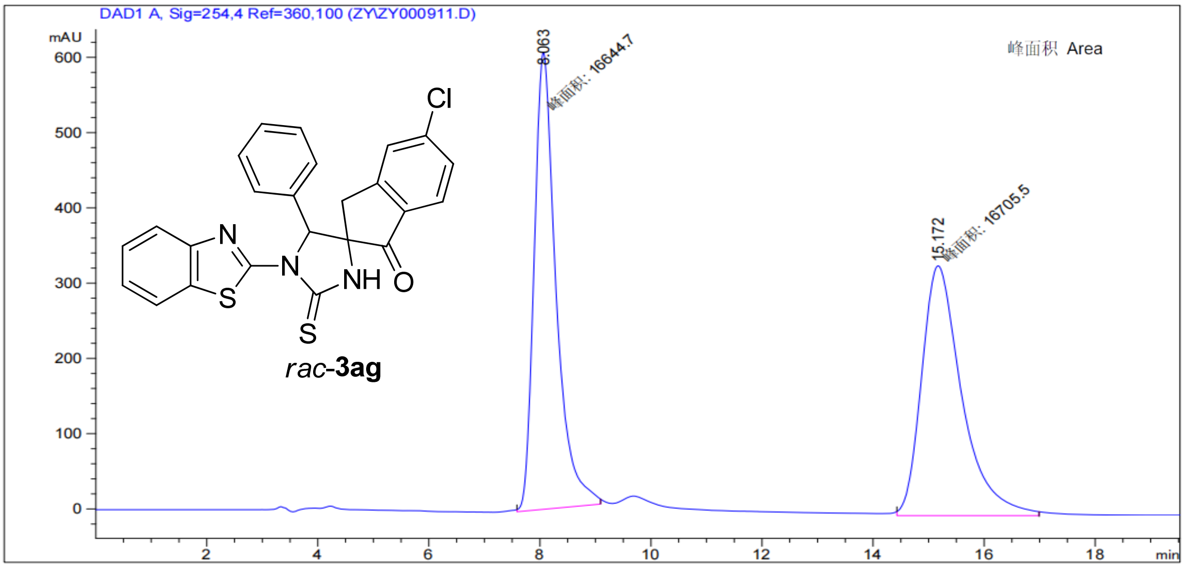

Peak #	RetTime [min]	Type	Width [min]	Area [mAU*s]	Height [mAU]	Area %
1	8.063	MM	0.4571	1.66447e4	606.95404	49.9089
2	15.172	MM	0.8387	1.67055e4	331.99033	50.0911

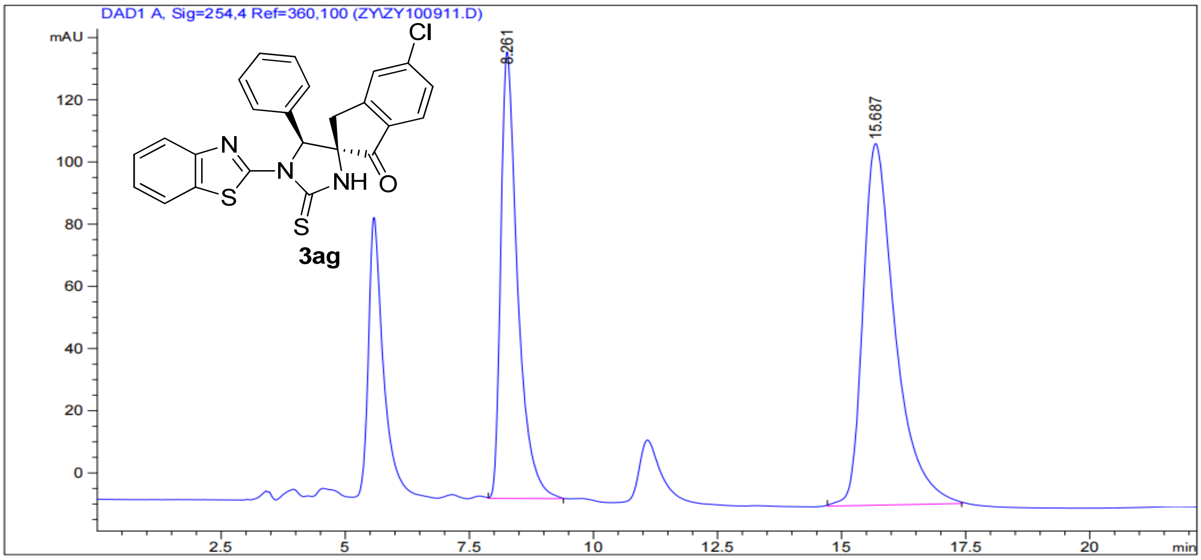

Peak #	RetTime [min]	Type	Width [min]	Area [mAU*s]	Height [mAU]	Area %
1	8.261	BB	0.3503	3401.48682	143.61298	39.2712
2	15.687	BB	0.6737	5260.05078	116.28135	60.7288

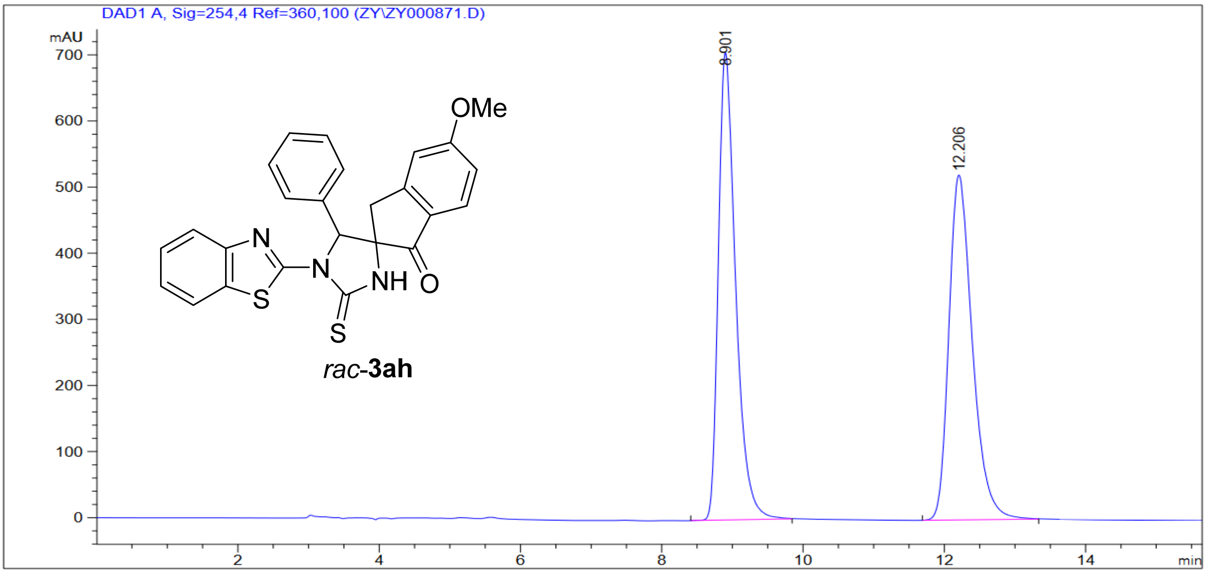

Peak #	RetTime [min]	Type	Width [min]	Area [mAU*s]	Height [mAU]	Area %
1	8.901	BB	0.2586	1.20354e4	706.87311	50.0299
2	12.206	BB	0.3529	1.20210e4	521.57898	49.9701

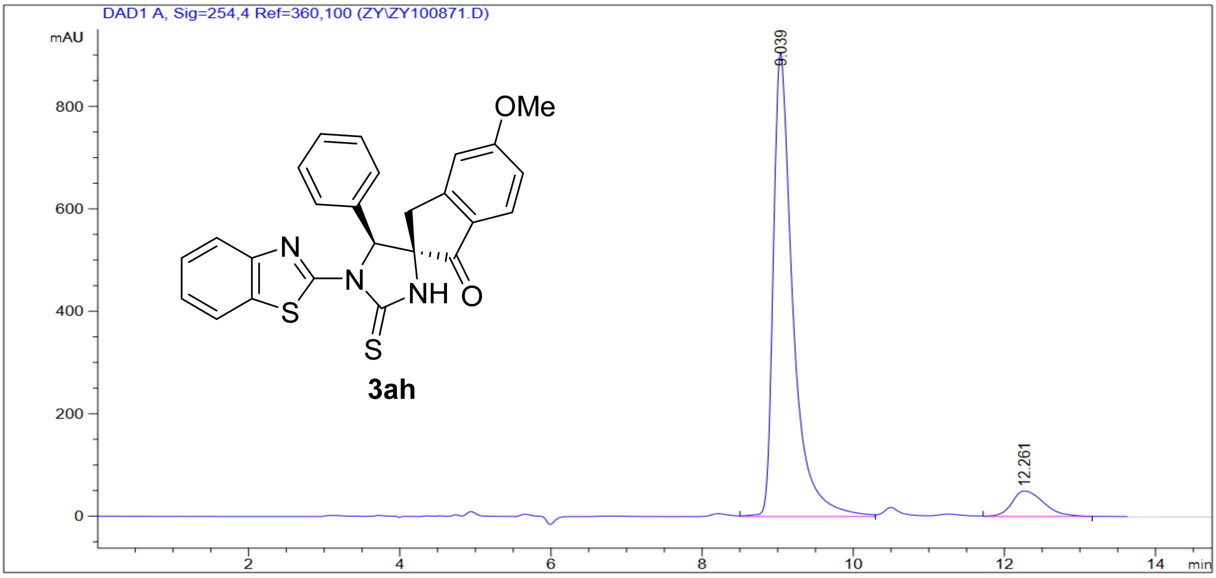

Peak #	RetTime [min]	Type	Width [min]	Area [mAU*s]	Height [mAU]	Area %
1	9.039	VV	0.2682	1.63026e4	904.66656	91.7493
2	12.261	VB	0.4521	1466.03577	50.00271	8.2507

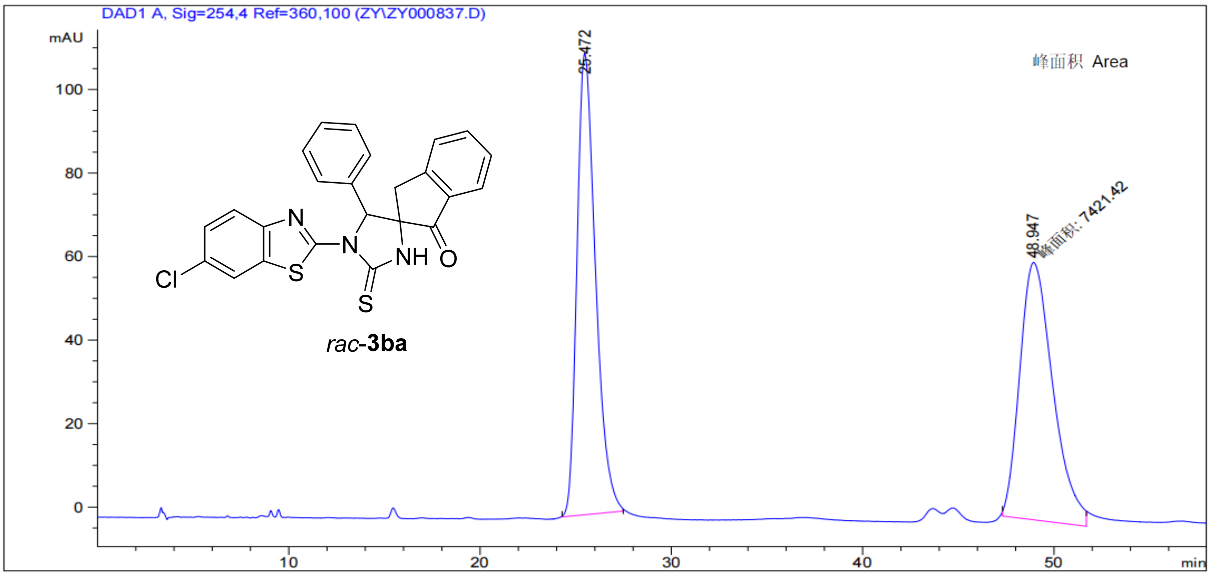

Peak #	RetTime [min]	Type	Width [min]	Area [mAU*s]	Height [mAU]	Area %
1	25.472	BB	1.0122	7445.99658	110.48976	50.0827
2	48.947	MM	2.0060	7421.41895	61.65995	49.9173

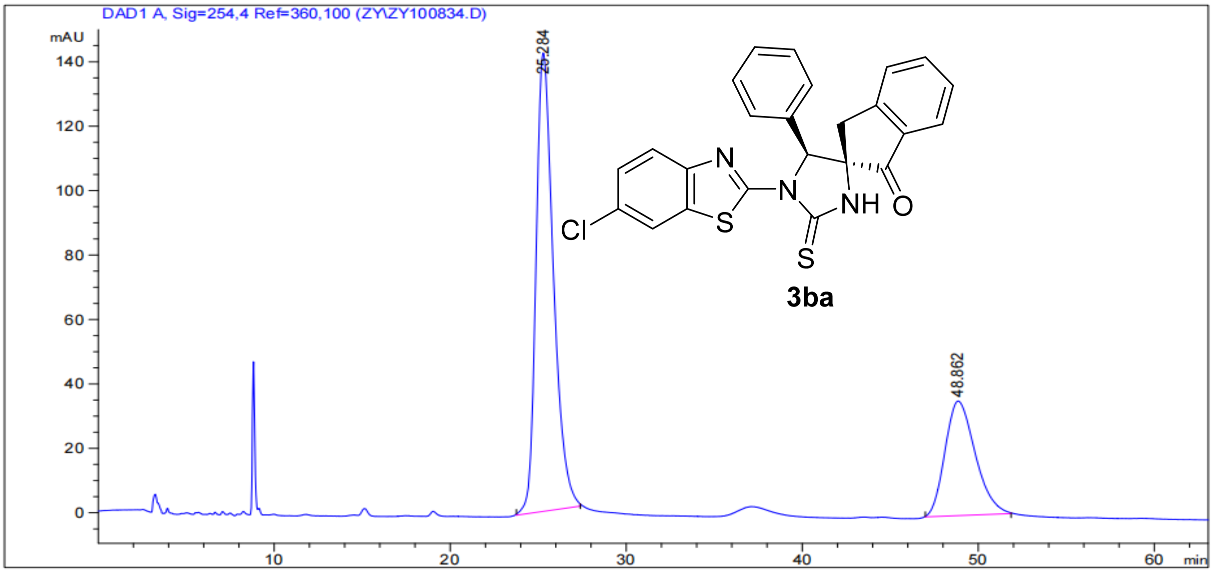

Peak #	RetTime [min]	Type	Width [min]	Area [mAU*s]	Height [mAU]	Area %
1	25.284	BB	1.0316	9778.74023	142.31329	69.7595
2	48.862	BB	1.3965	4239.05762	35.56564	30.2405

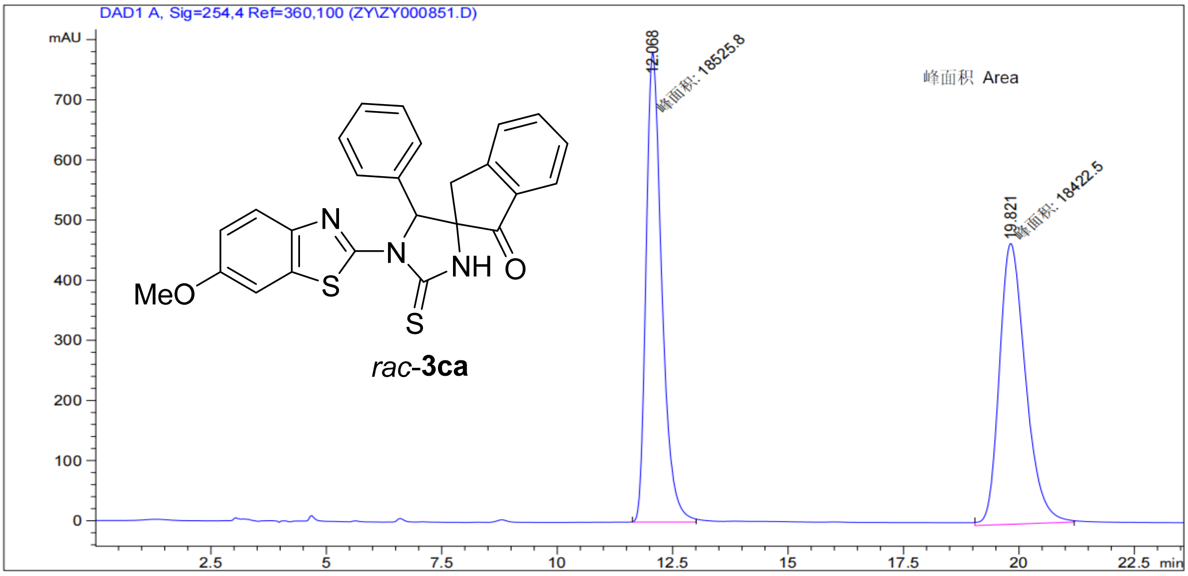

Peak #	RetTime [min]	Type	Width [min]	Area [mAU*s]	Height [mAU]	Area %
1	12.068	MM	0.3957	1.85258e4	780.32489	50.1398
2	19.821	MM	0.6576	1.84225e4	466.94281	49.8602

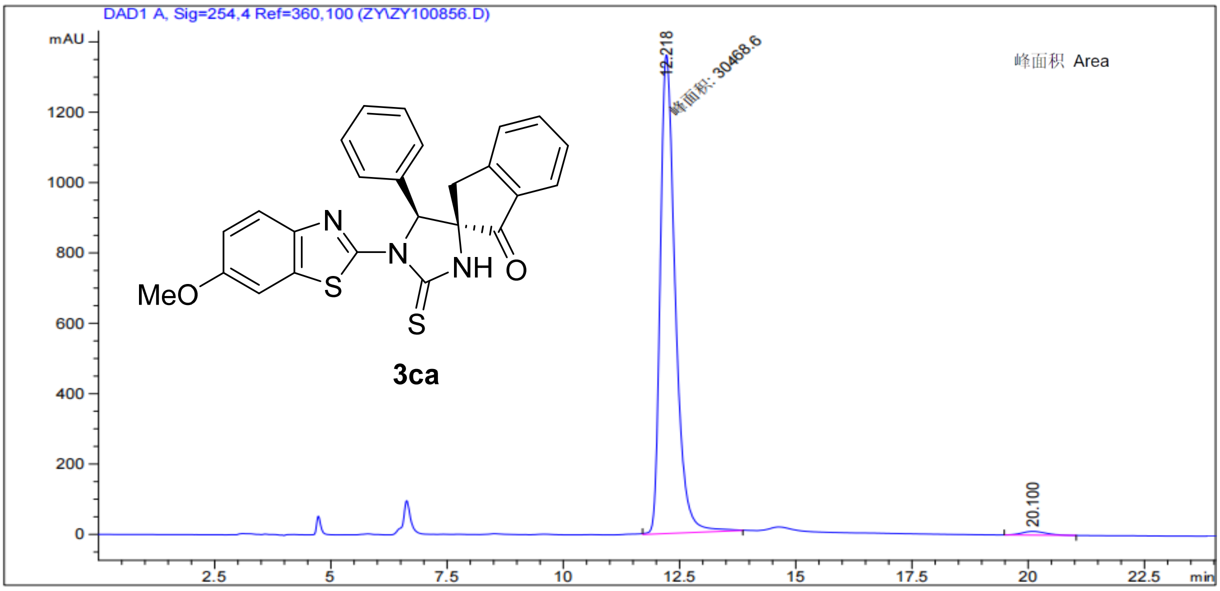

Peak #	RetTime [min]	Type	Width [min]	Area [mAU*s]	Height [mAU]	Area %
1	12.218	MM	0.3731	3.04686e4	1361.10913	98.7415
2	20.100	BB	0.5344	388.33139	10.62409	1.2585

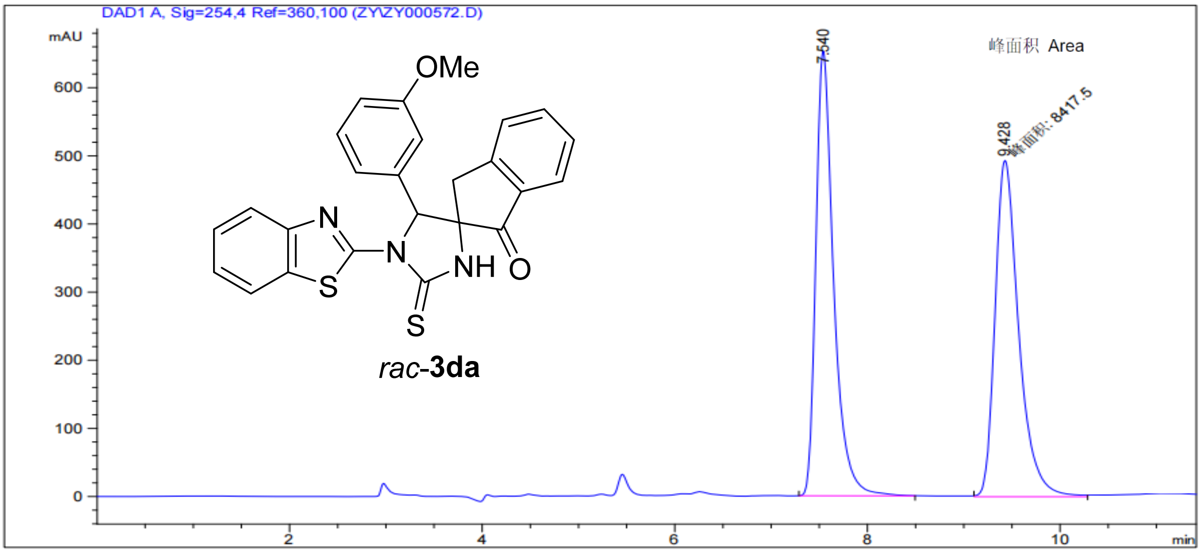

Peak #	RetTime [min]	Type	Width [min]	Area [mAU*s]	Height [mAU]	Area %
1	7.540	BB	0.1940	8392.28613	652.65869	49.9250
2	9.428	MM	0.2843	8417.49805	493.53262	50.0750

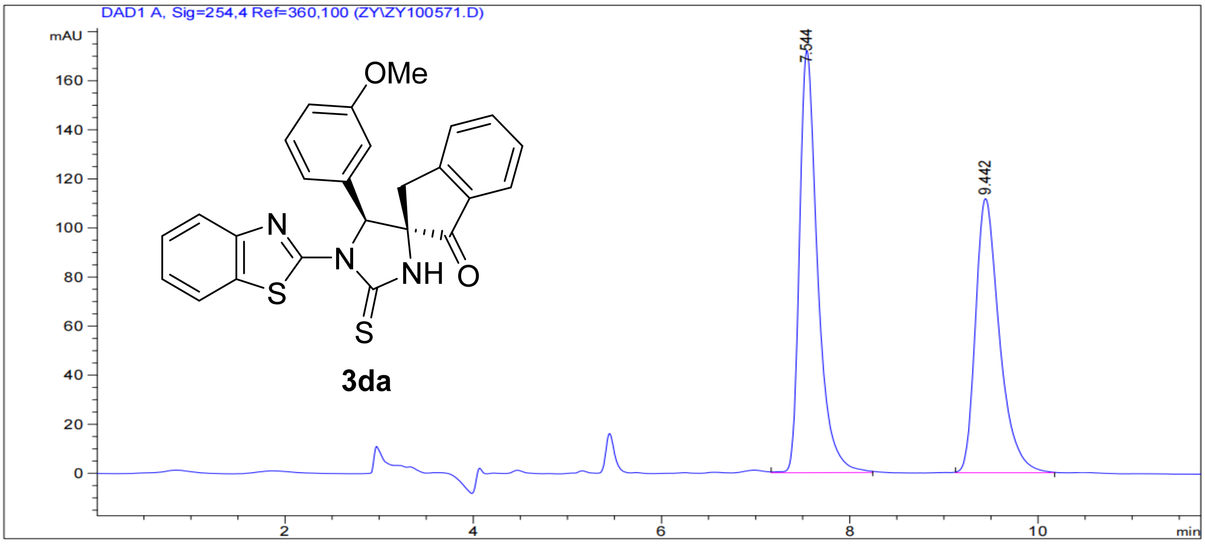

Peak #	RetTime [min]	Type	Width [min]	Area [mAU*s]	Height [mAU]	Area %
1	7.544	VB	0.2018	2298.62573	172.10153	53.8215
2	9.442	BB	0.2699	1972.20557	111.72974	46.1785

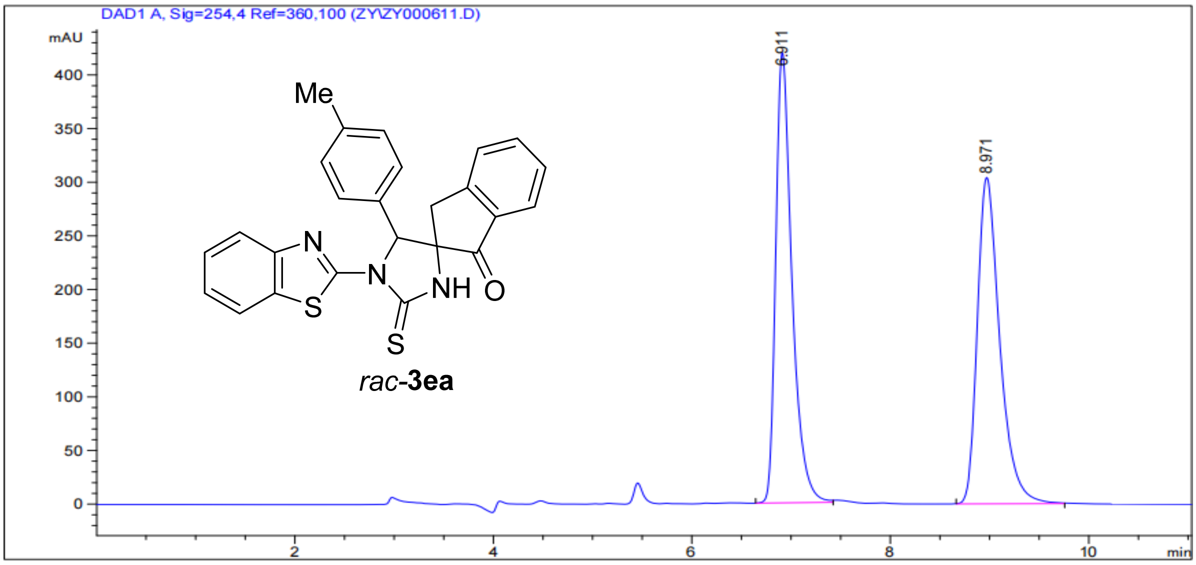

Peak #	RetTime [min]	Type	Width [min]	Area [mAU*s]	Height [mAU]	Area %
1	6.911	BB	0.1703	4759.18945	419.68997	49.8712
2	8.971	BB	0.2395	4783.76855	304.20016	50.1288

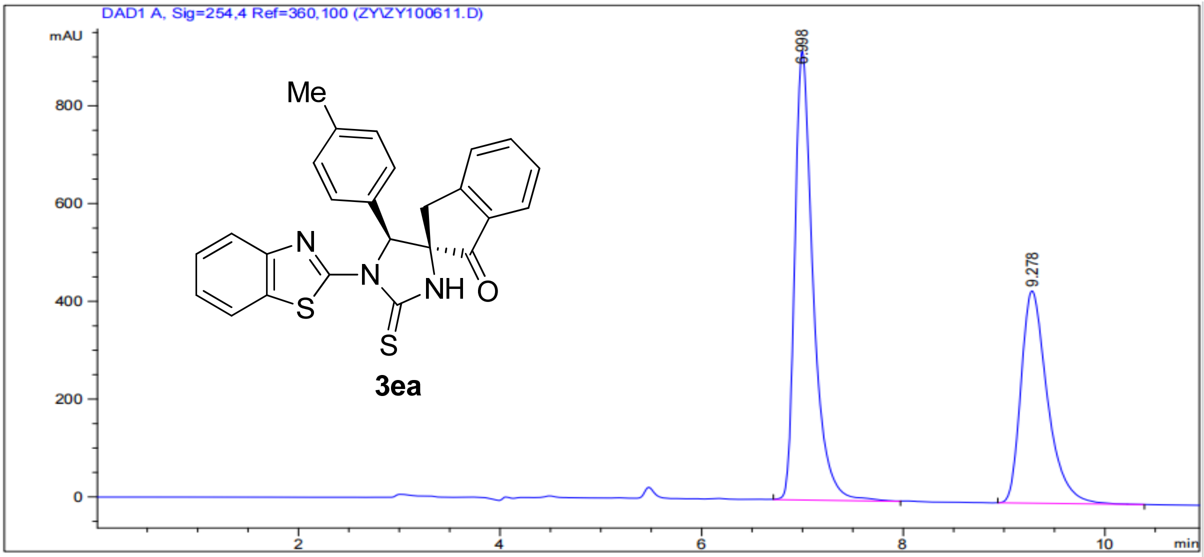

Peak #	RetTime [min]	Type	Width [min]	Area [mAU*s]	Height [mAU]	Area %
1	6.998	BB	0.1917	1.16174e4	917.42780	59.4000
2	9.278	BB	0.2773	7940.52783	434.36005	40.6000

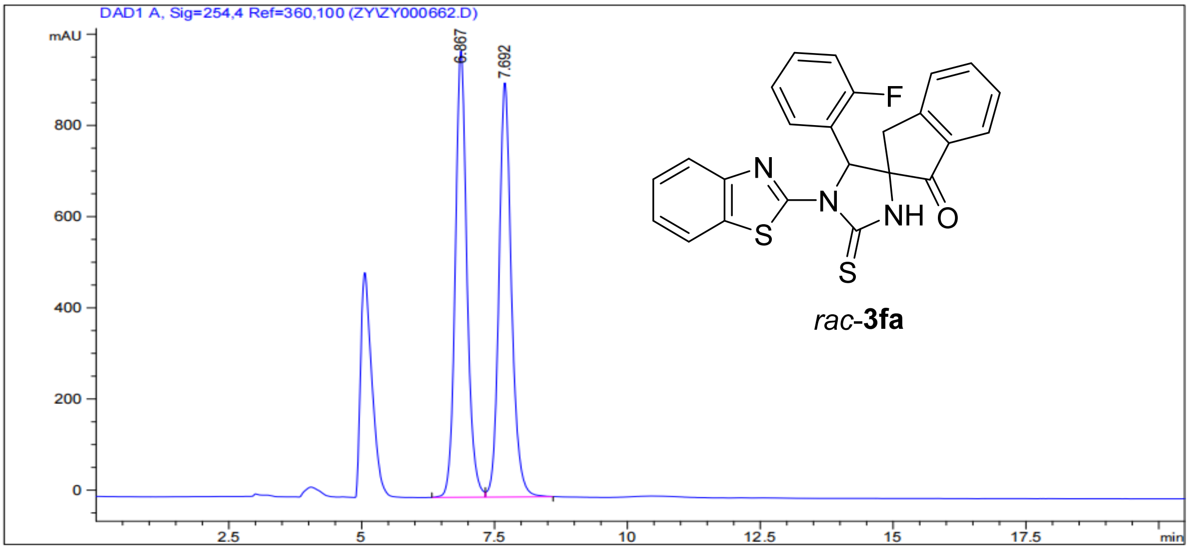

Peak #	RetTime [min]	Type	Width [min]	Area [mAU*s]	Height [mAU]	Area %
1	6.867	BV	0.2317	1.49187e4	979.31372	50.0607
2	7.692	VB	0.2471	1.48825e4	908.55011	49.9393

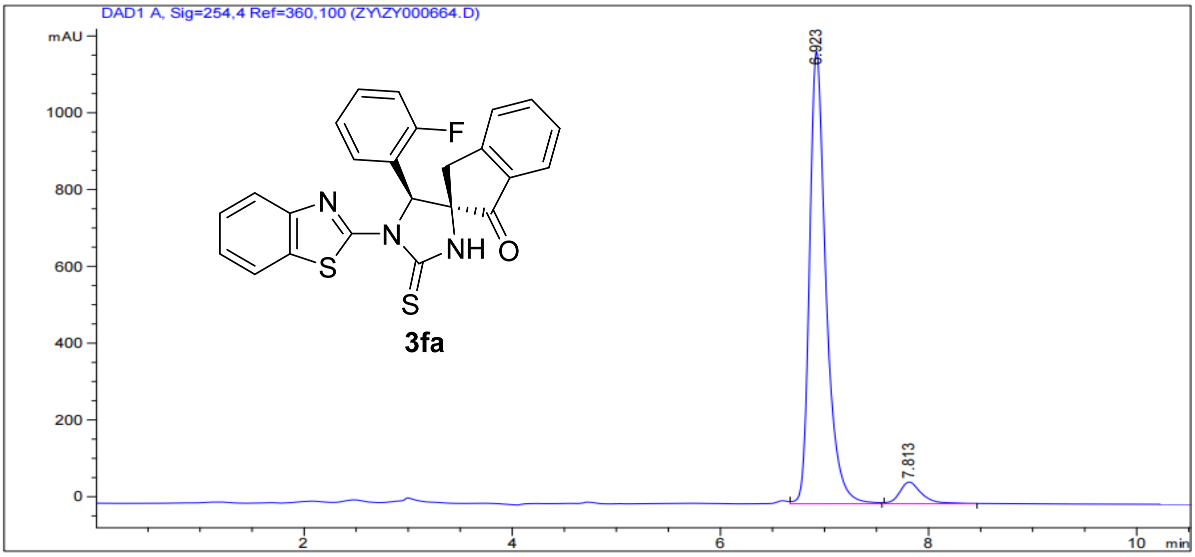

Peak #	RetTime [min]	Type	Width [min]	Area [mAU*s]	Height [mAU]	Area %
1	6.923	VB	0.1641	1.29286e4	1177.84607	93.9699
2	7.813	BB	0.2214	829.63287	56.46943	6.0301

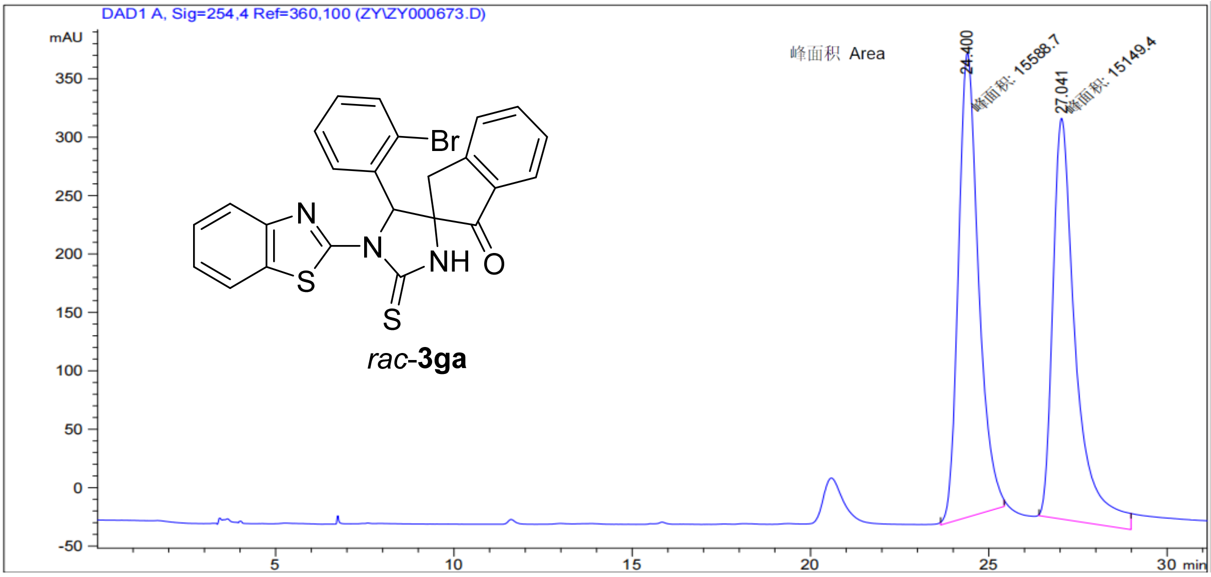

Peak #	RetTime [min]	Type	Width [min]	Area [mAU*s]	Height [mAU]	Area %
1	24.400	MM	0.6541	1.55887e4	397.20563	50.7146
2	27.041	MM	0.7359	1.51494e4	343.08807	49.2854

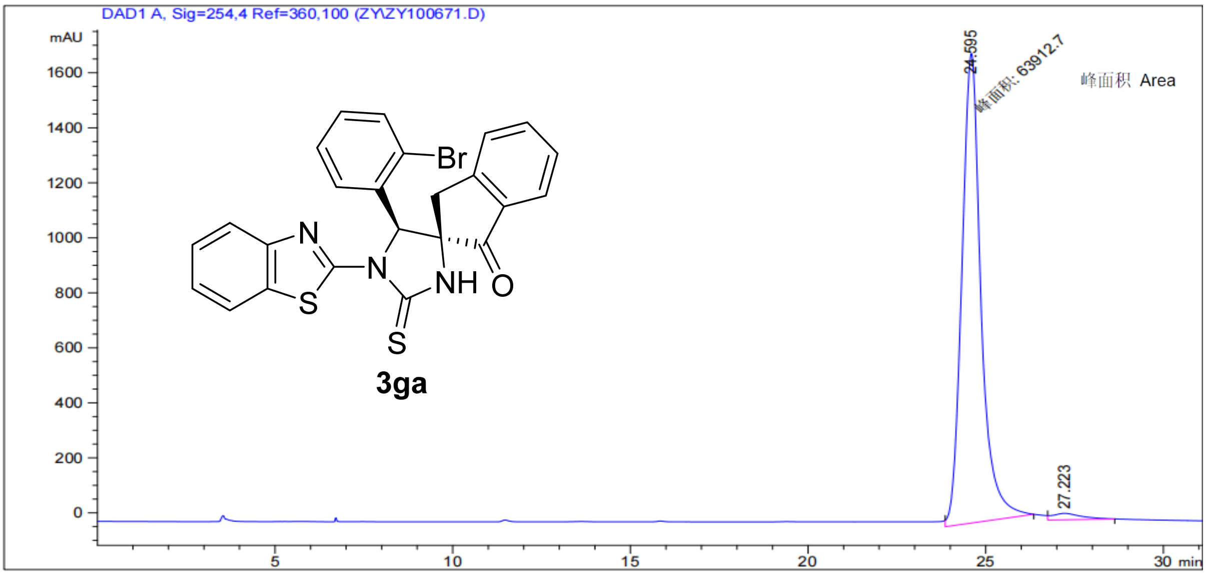

Peak #	RetTime [min]	Type	Width [min]	Area [mAU*s]	Height [mAU]	Area %
1	24.595	MM	0.6239	6.39127e4	1707.38354	97.8819
2	27.223	BB	0.7919	1383.01050	23.59256	2.1181

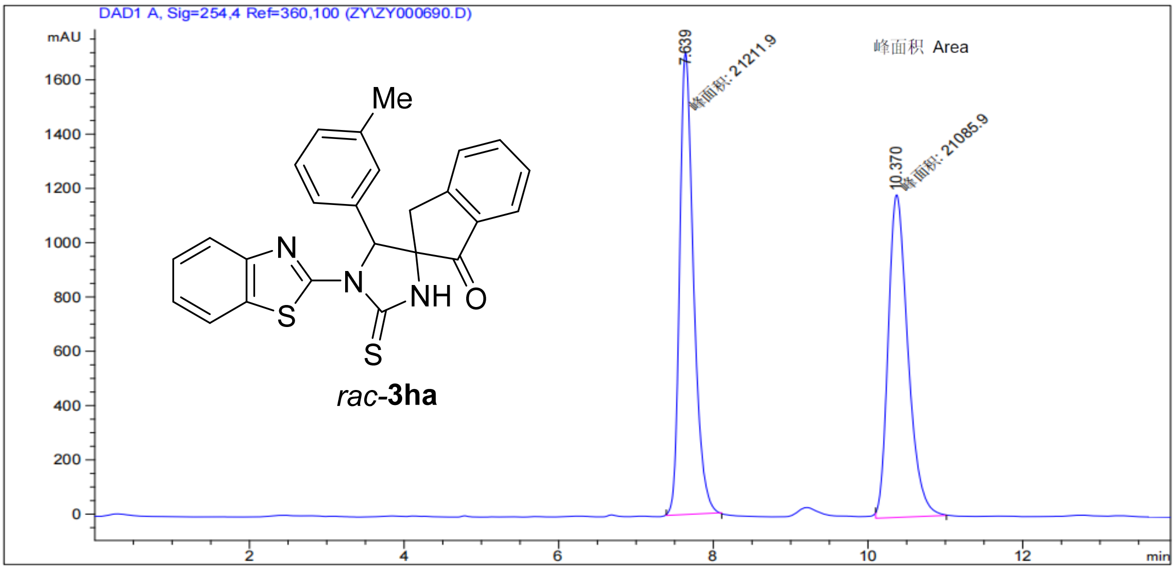

Peak #	RetTime [min]	Type	Width [min]	Area [mAU*s]	Height [mAU]	Area %
1	7.639	MM	0.2078	2.12119e4	1701.44788	50.1489
2	10.370	MM	0.2956	2.10859e4	1188.69678	49.8511

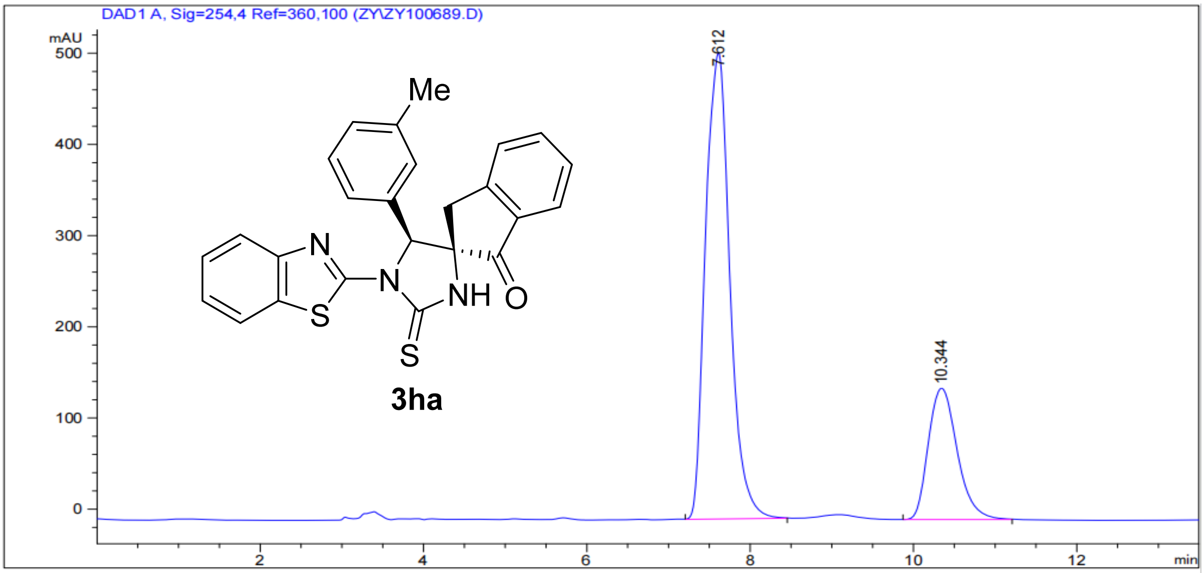

Peak #	RetTime [min]	Type	Width [min]	Area [mAU*s]	Height [mAU]	Area %
1	7.612	BB	0.3320	1.06005e4	511.10309	74.9837
2	10.344	BB	0.3865	3536.56055	144.01538	25.0163

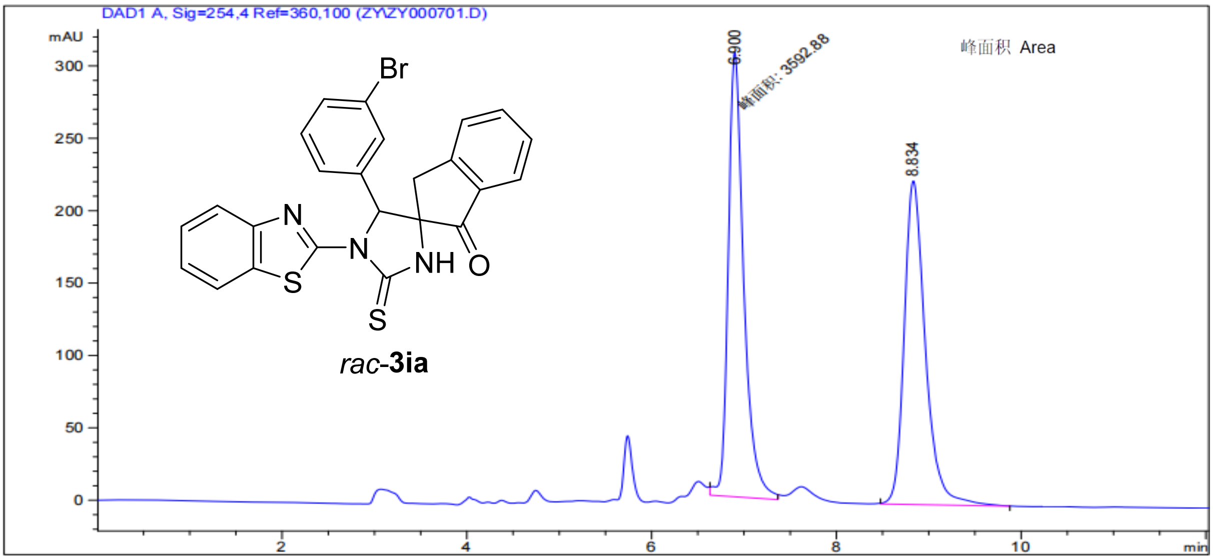

rac-**3ia**

Peak #	RetTime [min]	Type	Width [min]	Area [mAU*s]	Height [mAU]	Area %
1	6.900	MM	0.1949	3592.87500	307.19977	50.2773
2	8.834	BB	0.2395	3553.24097	223.49901	49.7227

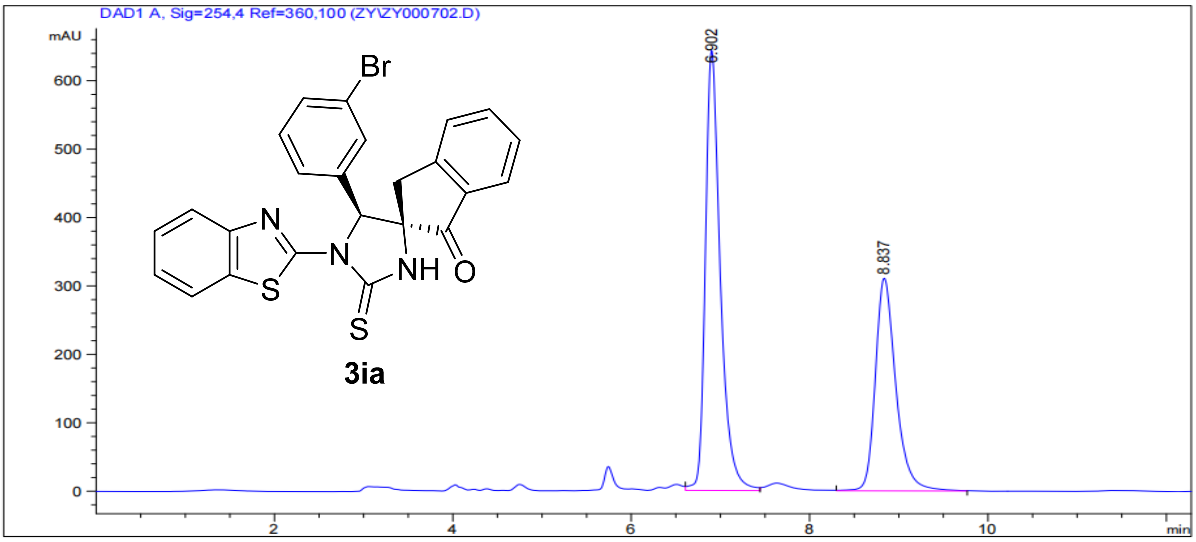

3ia

Peak #	RetTime [min]	Type	Width [min]	Area [mAU*s]	Height [mAU]	Area %
1	6.902	VV	0.1740	7499.89990	643.15515	60.1199
2	8.837	VB	0.2427	4975.00781	310.89423	39.8801

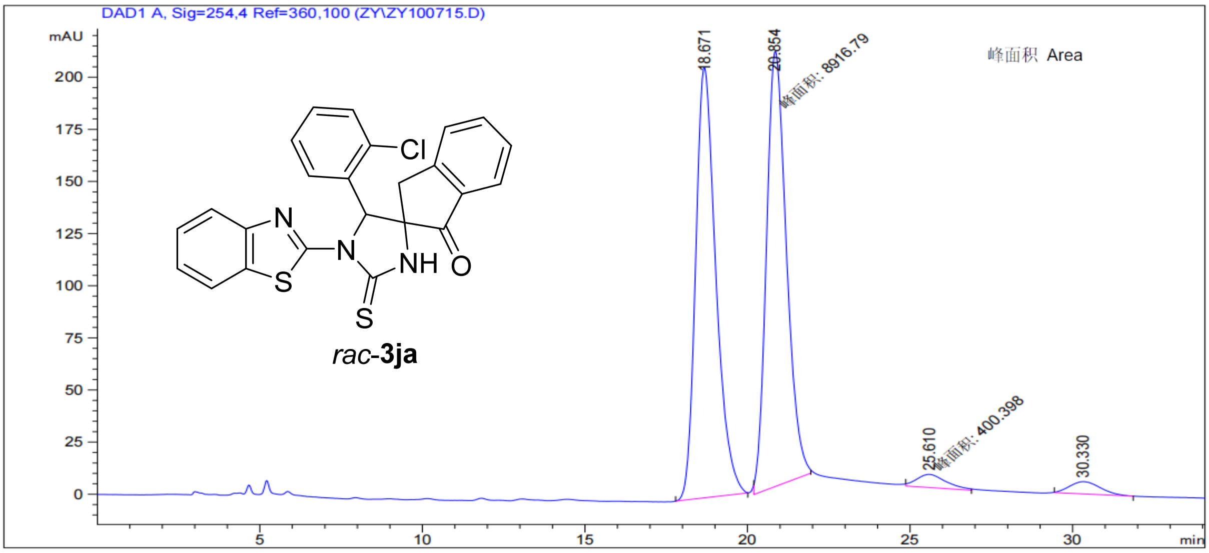

Peak #	RetTime [min]	Type	Width [min]	Area [mAU*s]	Height [mAU]	Area %
1	18.671	BB	0.6560	8917.63281	206.42331	47.8942
2	20.854	MM	0.7121	8916.78613	208.68503	47.8896
3	25.610	MM	1.0576	400.39819	6.30967	2.1504
4	30.330	BB	0.7710	384.62735	5.88133	2.0657

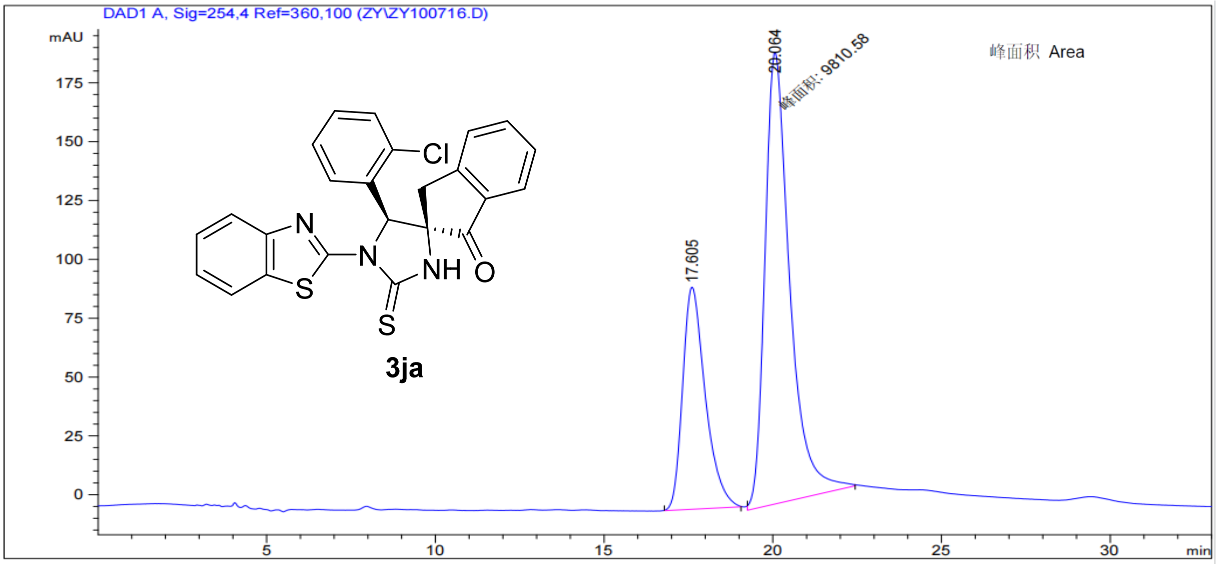

Peak #	RetTime [min]	Type	Width [min]	Area [mAU*s]	Height [mAU]	Area %
1	17.605	BB	0.7032	4409.46875	94.35020	31.0088
2	20.064	MM	0.8520	9810.58008	191.90762	68.9912

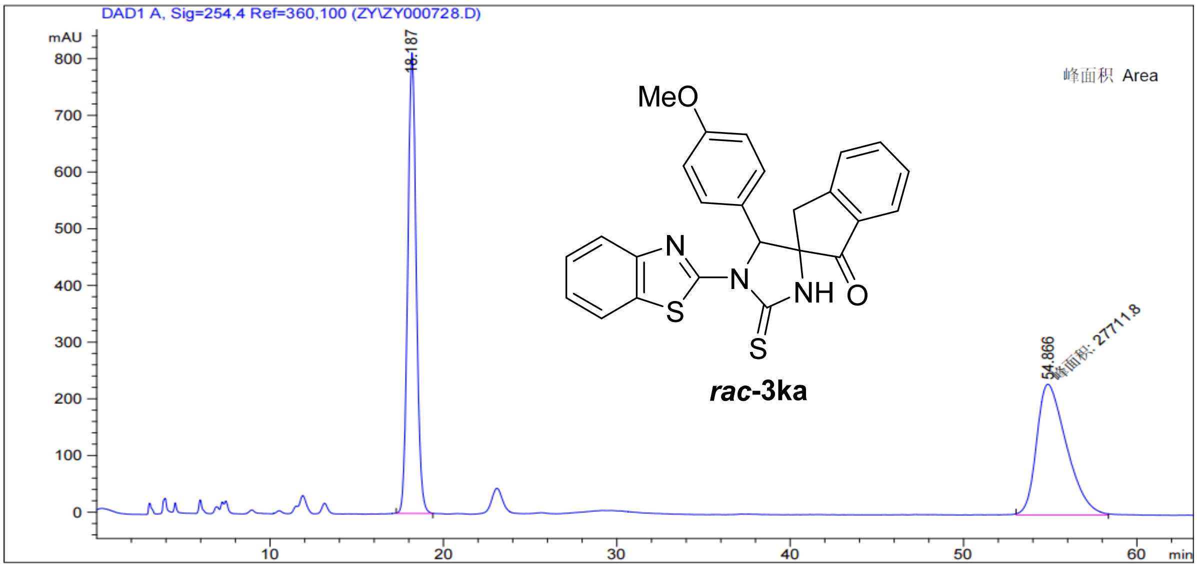

Peak #	RetTime [min]	Type	Width [min]	Area [mAU*s]	Height [mAU]	Area %
1	18.187	BB	0.5279	2.77222e4	812.77924	50.0094
2	54.866	MM	2.0020	2.77118e4	230.70424	49.9906

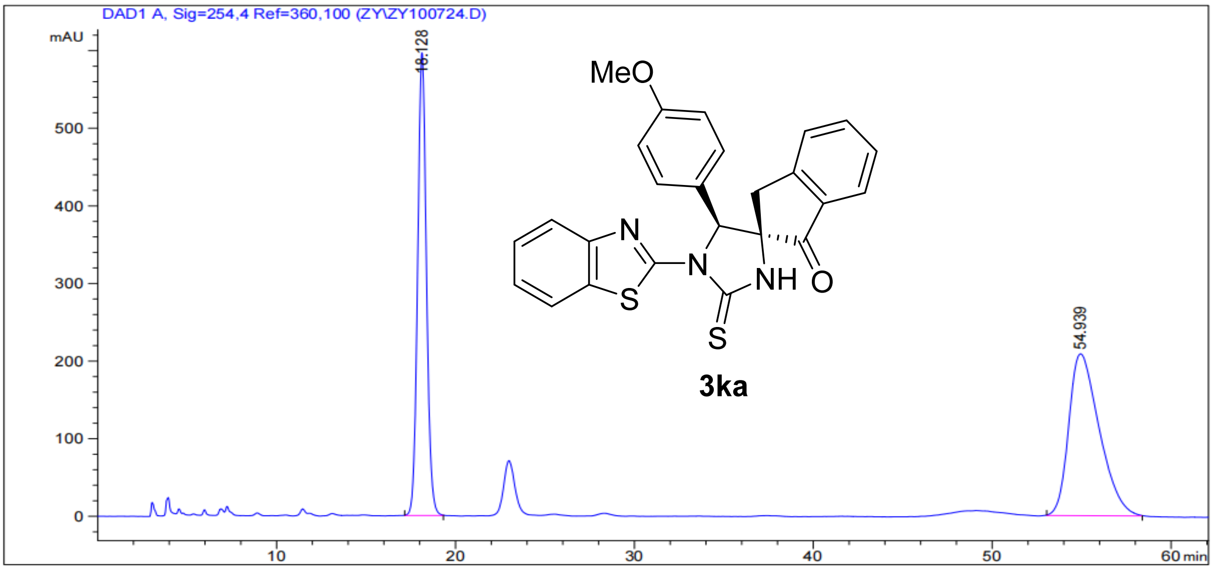

Peak #	RetTime [min]	Type	Width [min]	Area [mAU*s]	Height [mAU]	Area %
1	18.128	BB	0.5261	2.03466e4	596.27802	45.1930
2	54.939	BB	1.6909	2.46750e4	208.47401	54.8070

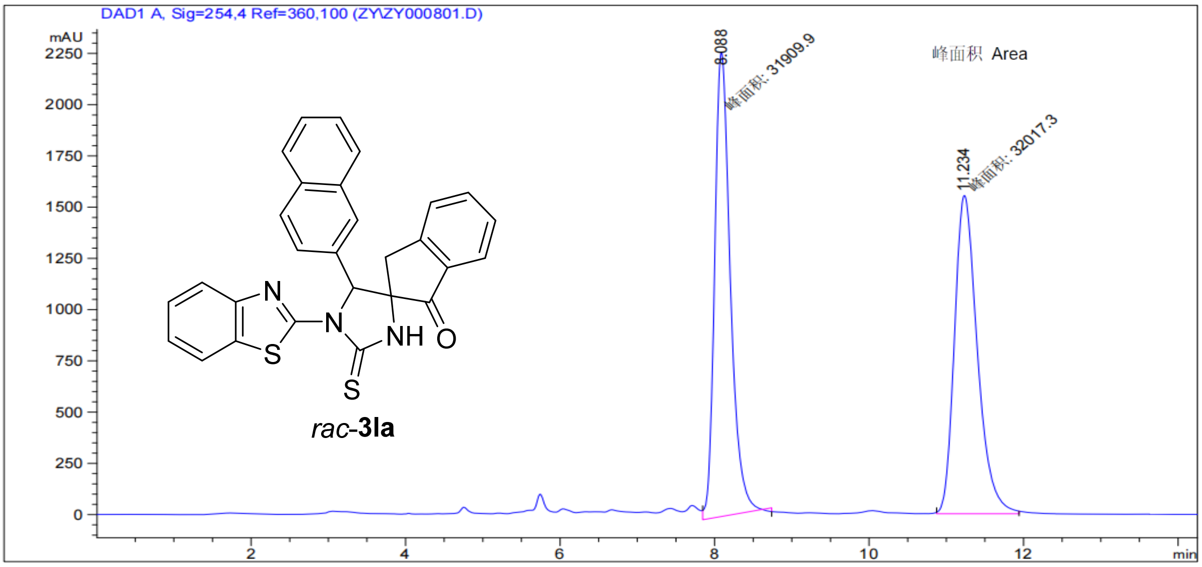

Peak #	RetTime [min]	Type	Width [min]	Area [mAU*s]	Height [mAU]	Area %
1	8.088	MM	0.2347	3.19099e4	2266.16089	49.9160
2	11.234	MM	0.3436	3.20173e4	1553.08350	50.0840

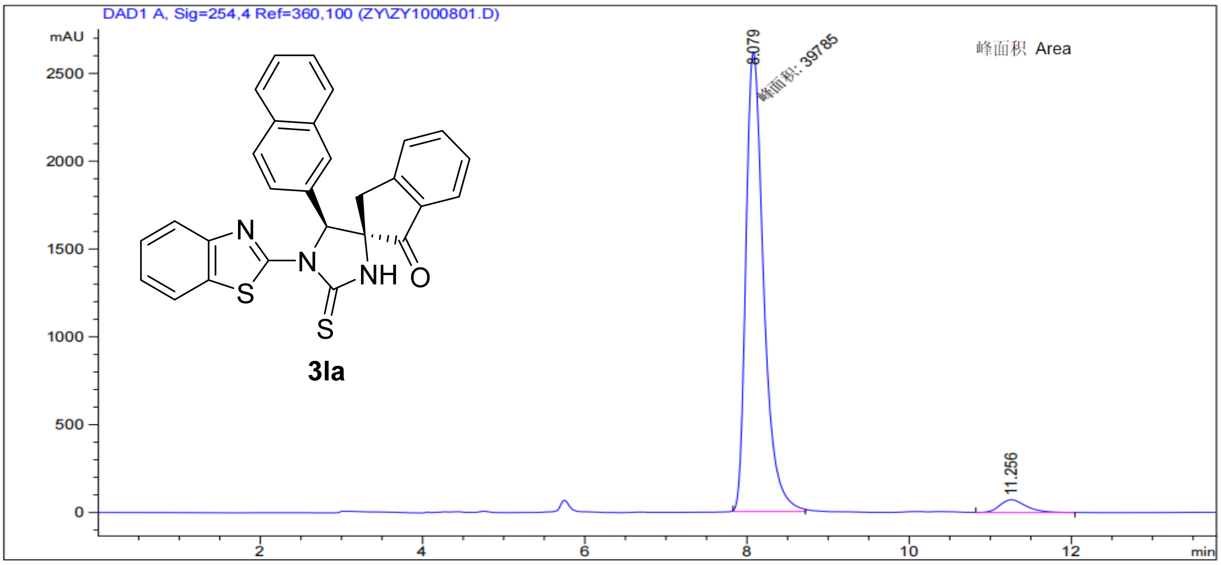

Peak #	RetTime [min]	Type	Width [min]	Area [mAU*s]	Height [mAU]	Area %
1	8.079	MM	0.2535	3.97850e4	2615.97339	96.0204
2	11.256	VB	0.3440	1648.92615	72.87273	3.9796

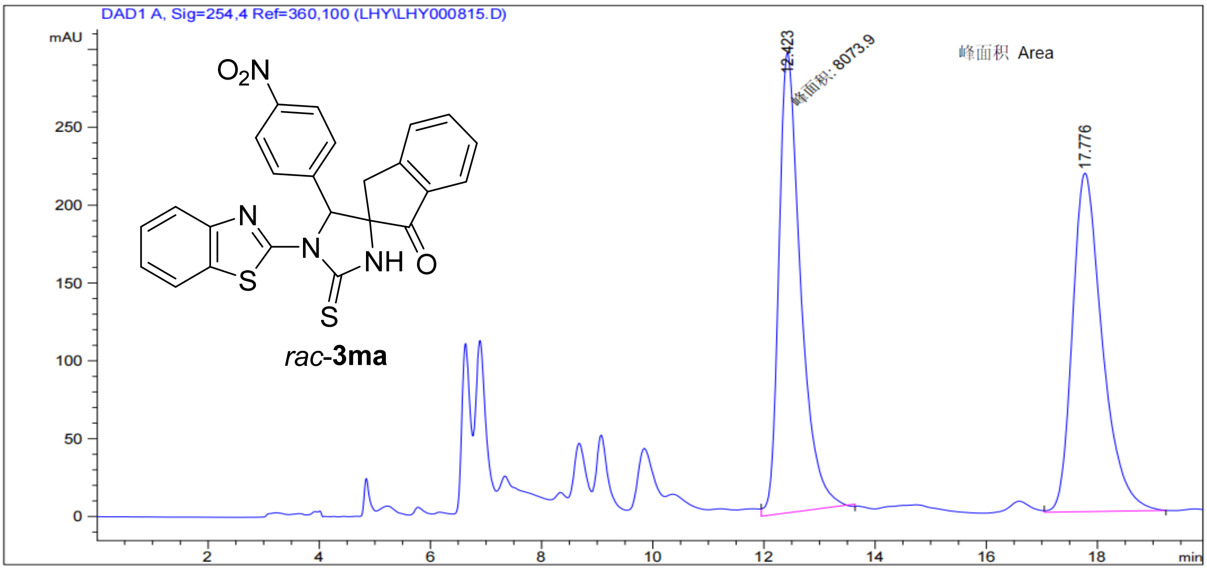

Peak #	RetTime [min]	Type	Width [min]	Area [mAU*s]	Height [mAU]	Area %
1	12.423	MM	0.4553	8073.89697	295.55994	49.8712
2	17.776	VB	0.5633	8115.60791	217.49779	50.1288

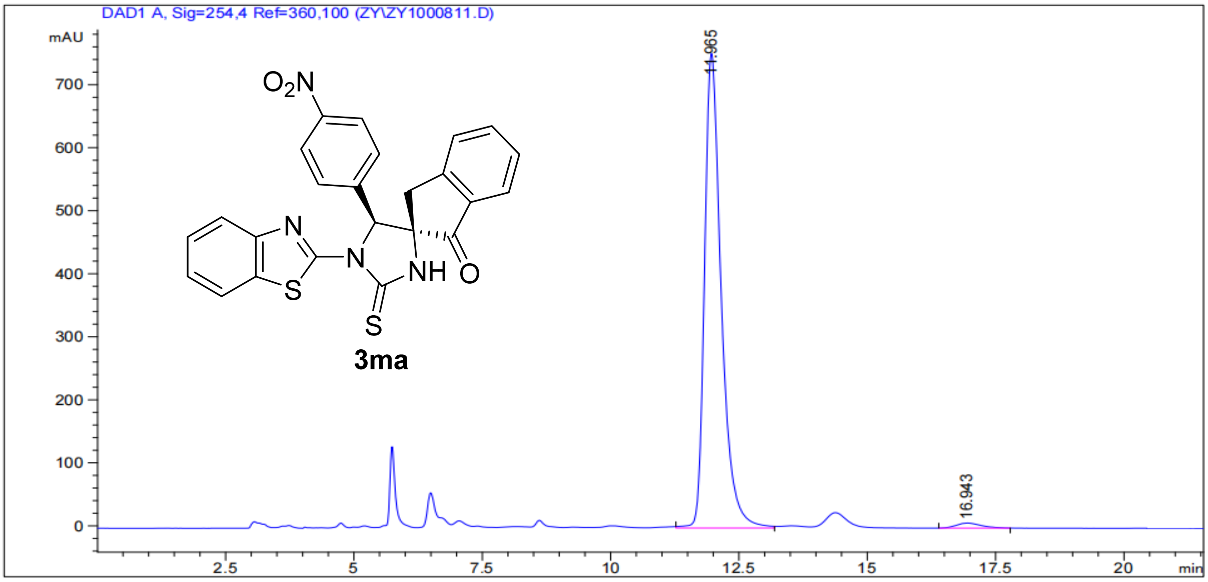

Peak #	RetTime [min]	Type	Width [min]	Area [mAU*s]	Height [mAU]	Area %
1	11.965	BB	0.3423	1.70581e4	752.83167	98.3994
2	16.943	BB	0.5034	277.47382	8.06404	1.6006

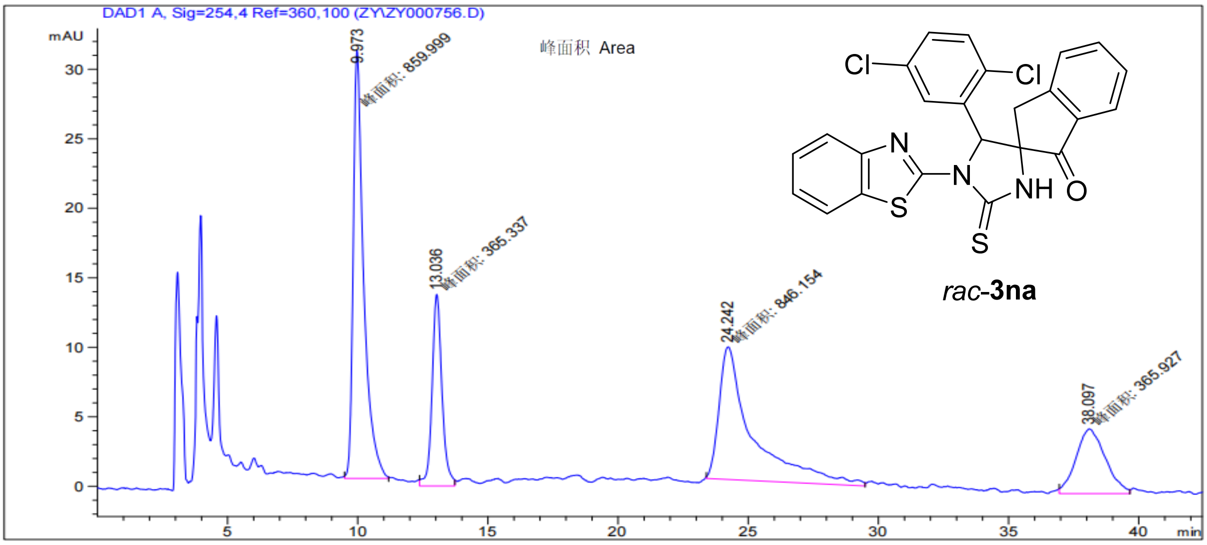

Peak #	RetTime [min]	Type	Width [min]	Area [mAU*s]	Height [mAU]	Area %
1	9.973	MM	0.4659	859.99908	30.76487	35.2832
2	13.036	MM	0.4415	365.33719	13.79301	14.9887
3	24.242	MM	1.4790	846.15424	9.53539	34.7152
4	38.097	MM	1.3068	365.92697	4.66690	15.0129

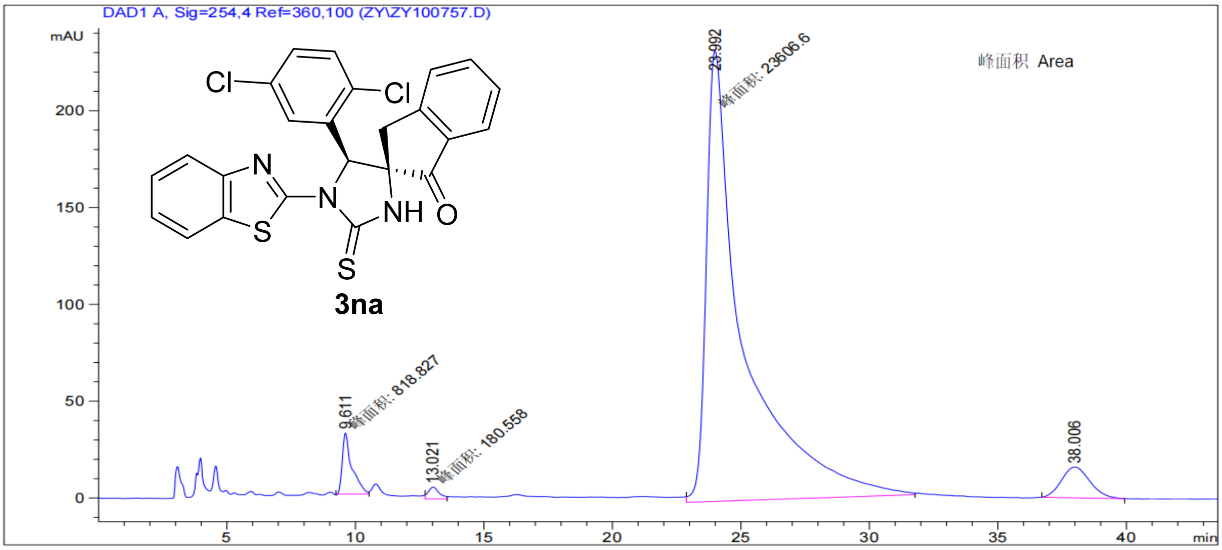

Peak #	RetTime [min]	Type	Width [min]	Area [mAU*s]	Height [mAU]	Area %
1	9.611	MM	0.4341	818.82690	31.43702	3.1700
2	13.021	MM	0.4956	180.55811	6.07166	0.6990
3	23.992	MM	1.6909	2.36066e4	232.68864	91.3908
4	38.006	BB	0.9161	1224.41577	15.90485	4.7402
